# Supplementary material for: Allopolyploid origin and diversification of the Hawaiian endemic mints
Source: Nat Commun. 2024 Apr 10;15:3109. doi: 10.1038/s41467-024-47247-y (PMC11006916; doi:10.1038/s41467-024-47247-y)
Supplement: Supplementary file 1 — Supplementary Information [file 41467_2024_47247_MOESM1_ESM.pdf]

Allopolyploid origin and diversification of the Hawaiian endemic mints

Crystal M. Tomlin[1], Sitaram Rajaraman[2,3], Jeanne Theresa Sebesta[1], Anne-Cathrine Scheen[4], Mika Bendiksby[5], Yee Wen Low[6], Jarkko Salojärvi[2,3], Todd P. Michael[7], Victor A. Albert[1,*], Charlotte Lindqvist[1,*]

[1]Department of Biological Sciences, University at Buffalo, New York, USA.
[2]School of Biological Sciences, Nanyang Technological University, Singapore.
[3]Organismal and Evolutionary Biology Research Programme, Faculty of Biological and Environmental Sciences, University of Helsinki, Helsinki, Finland.
[4]Stavanger Botanic Garden, City of Stavanger, Norway.
[5]Natural History Museum, University of Oslo, Oslo, Norway.
[6]Singapore Botanic Gardens, National Parks Board, Singapore.
[7]The Plant Molecular and Cellular Biology Laboratory, Salk Institute for Biological Studies, La Jolla, California, USA.
[*]Correspondence to Charlotte Lindqvist (cl243@buffalo.edu) or Victor A. Albert (vaalbert@buffalo.edu).

SUPPLEMENTARY INFORMATION:

Supplementary Figures 1 – 32

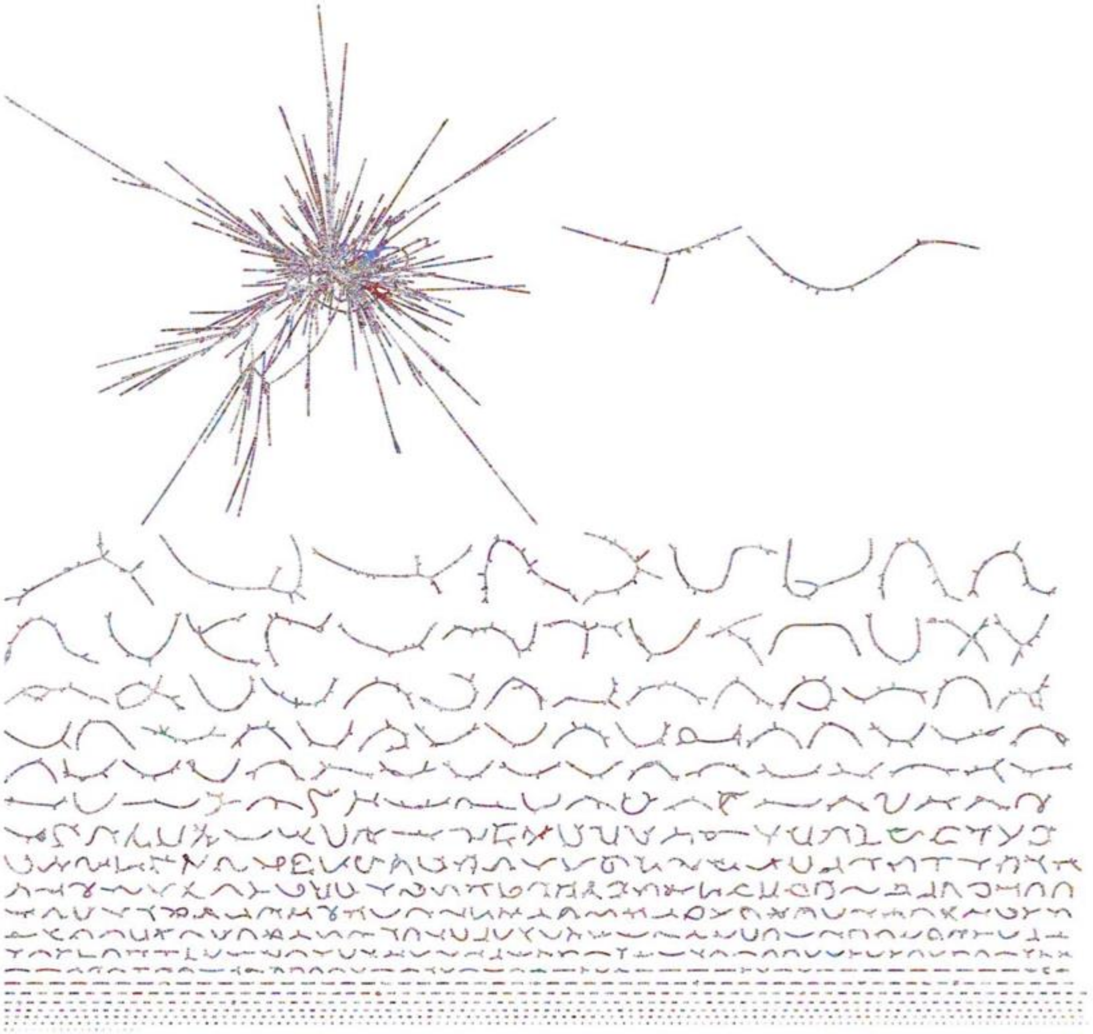

Supplementary Fig. 1: Bandage graph with RepeatModeler2 consensus TE library used as a BLAST search against the raw assembly, with BLAST hits colored in rainbow colors.

Supplementary Fig. 2

a

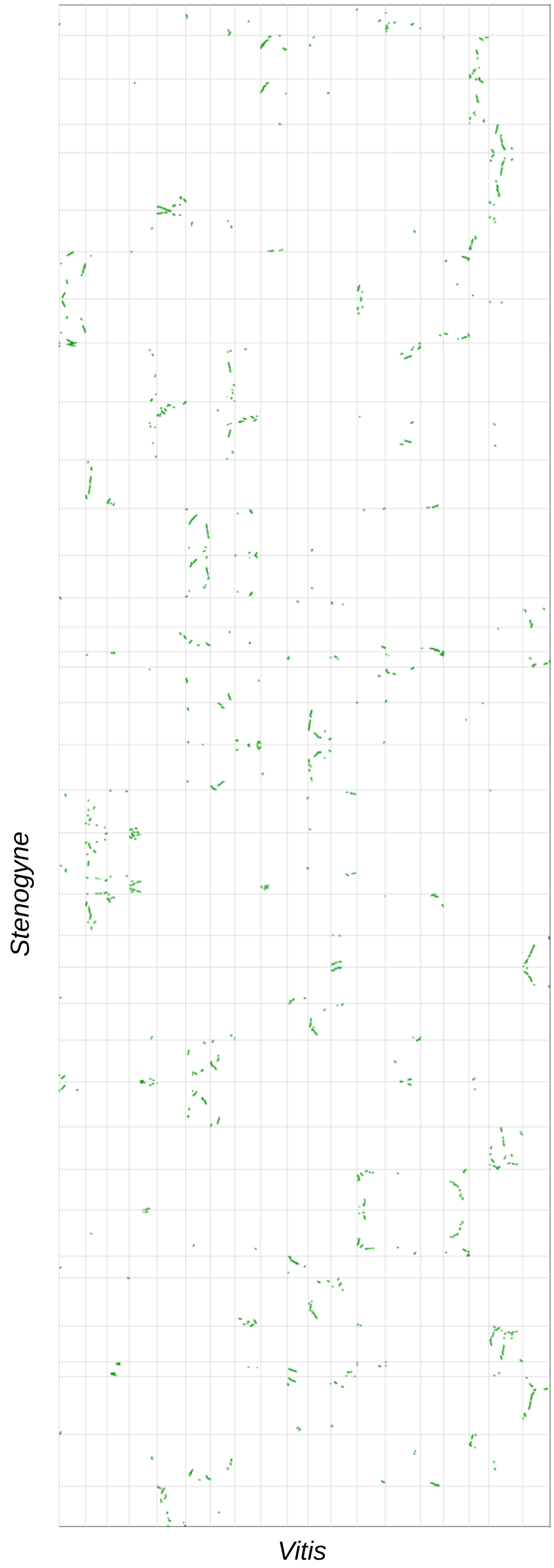

b

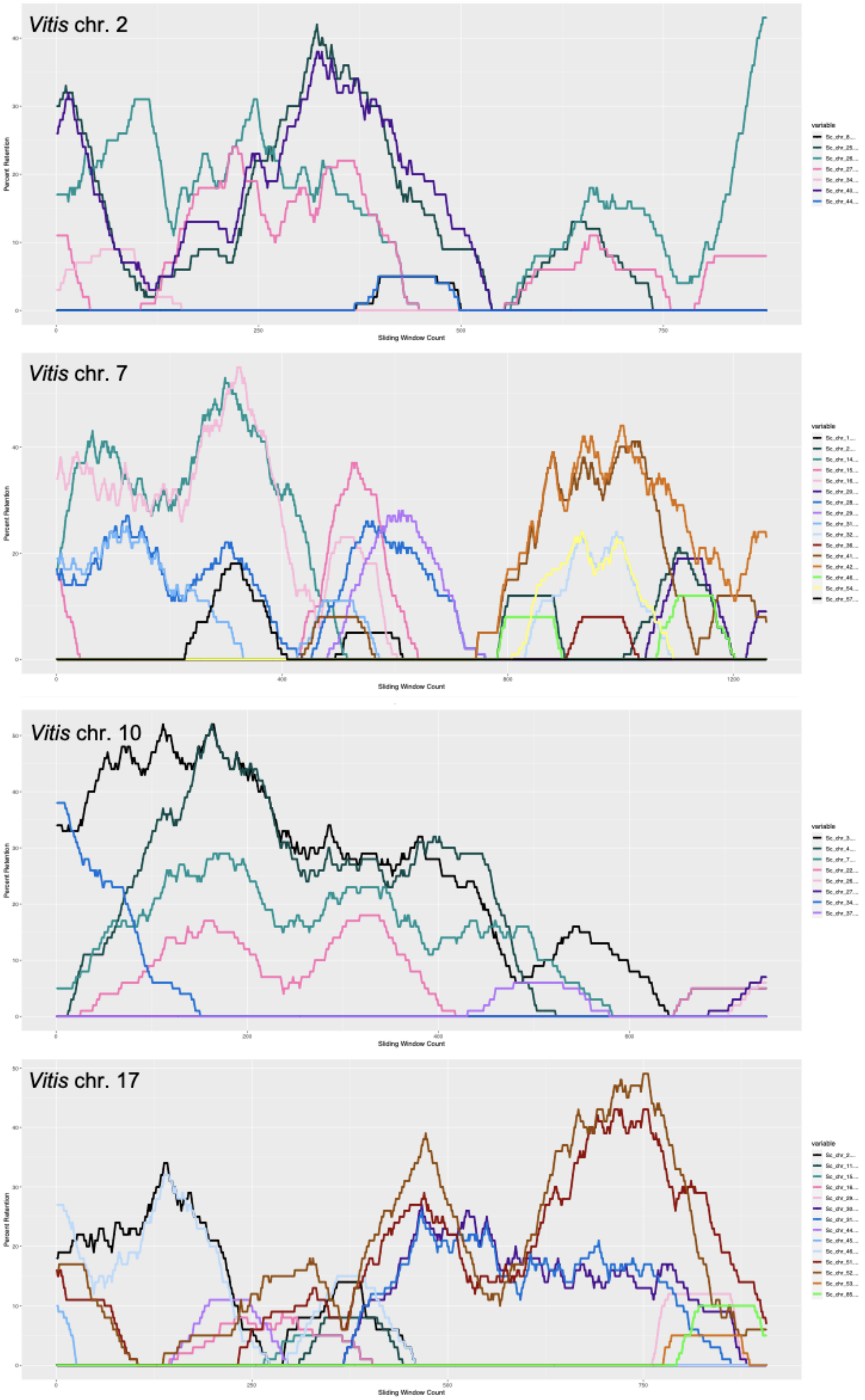

Supplementary Fig. 2: a CoGe SynMap of *Stenogyne* vs. *Vitis*. Examples of 4:1 syntenic regions are highlighted in light green. **b** Exemplar CoGe FractBias plots of *Stenogyne* mapped to *Vitis* chromosomes (chromosome numbers 2, 7, 10, and 17 shown). Colored lines represent different scaffolds of the *Stenogyne calaminthoides* genome assembly.

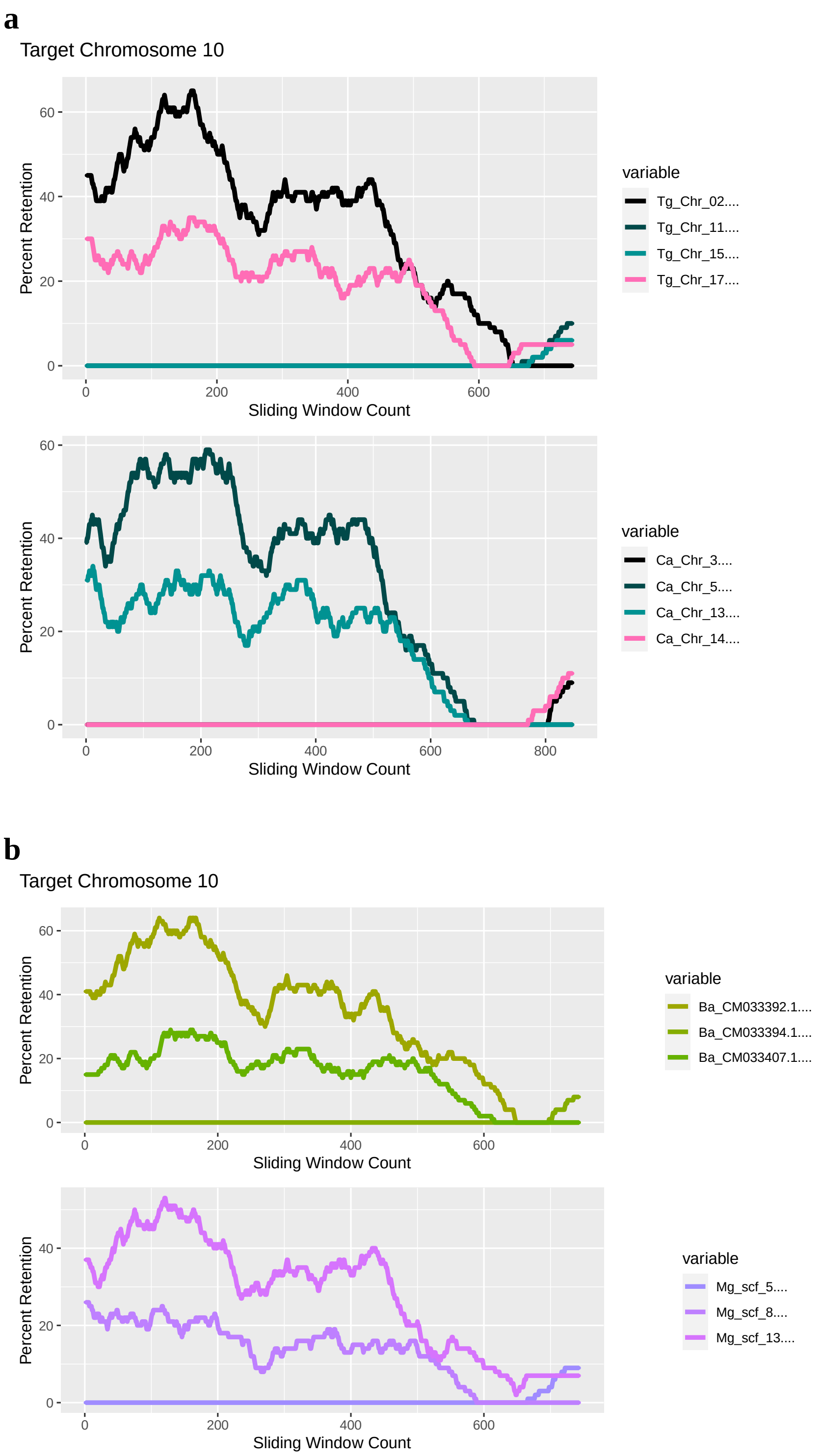

Supplementary Fig. 3: Fractionation bias shown for four species in Lamiales mapped to *Vitis*. **a** CoGe FractBias plots of *Tectona* (top) and *Callicarpa* (bottom) mapped to *Vitis* chromosome 10 **b** CoGe FractBias plots of *Buddleja* (top) and *Mimulus* (bottom) mapped to *Vitis* chromosome 10. Colored lines represent different scaffolds in the query genome assemblies.

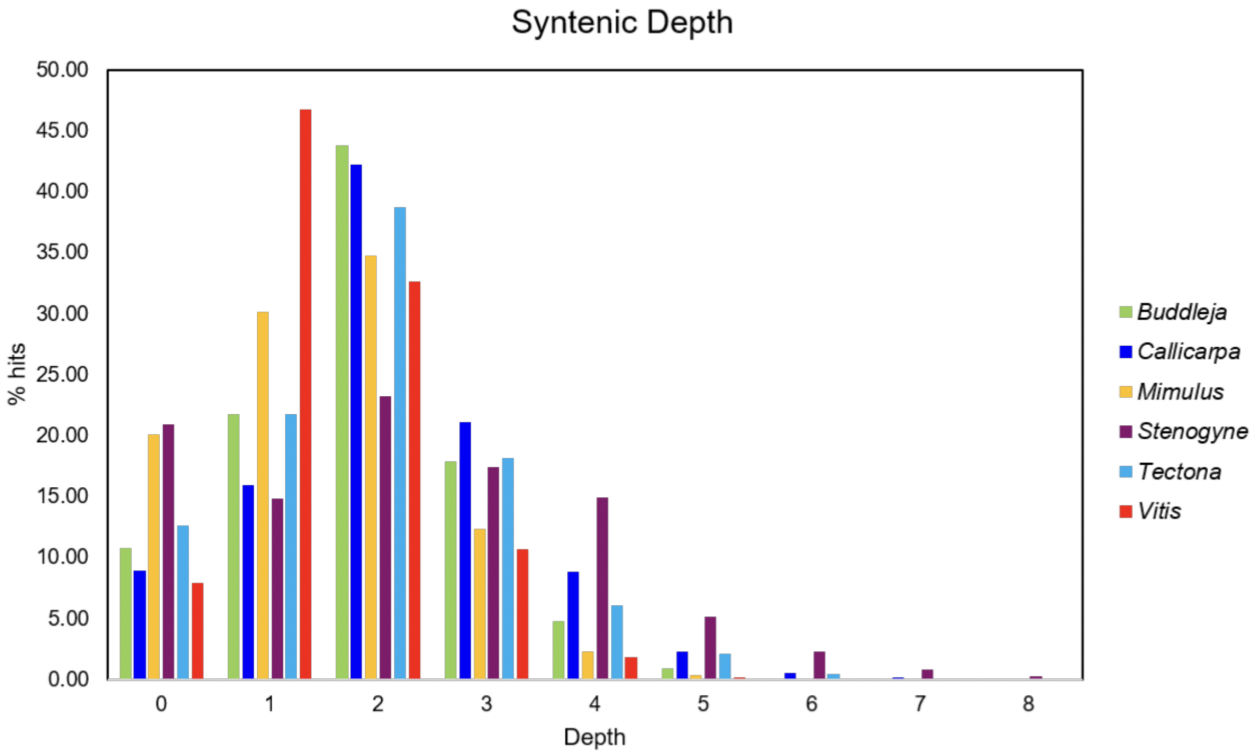

Supplementary Fig. 4: Syntenic depth analysis using *Arabidopsis* TFL1 (AT5G03840) as the seed, as calculated in the CoGe SynFind platform.

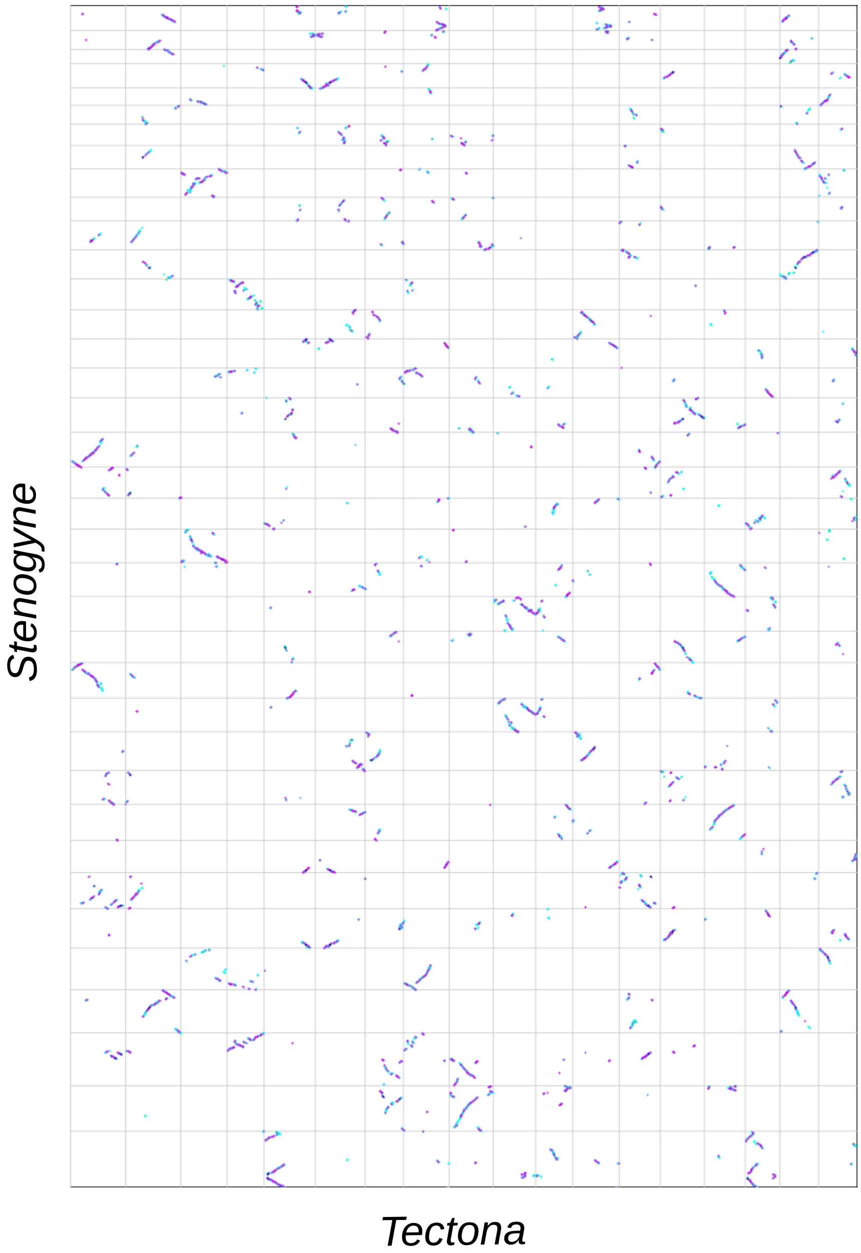

Supplementary Fig. 5: SynMap of *Stenogyne* vs. *Tectona* with 2:1 syntenic regions highlighted.

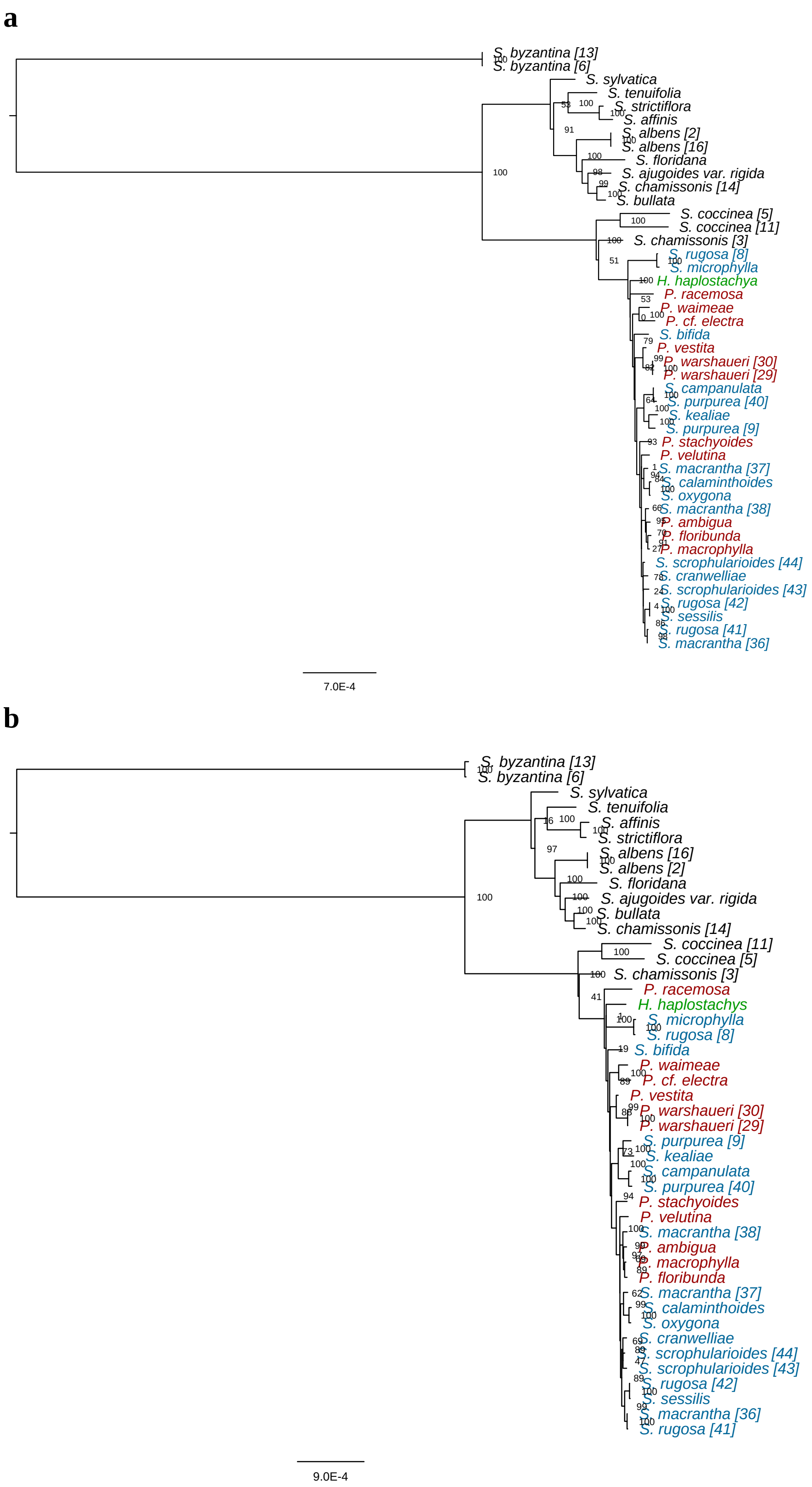

Supplementary Fig. 6: Phylogenies based on chloroplast data. **a** *de novo* assembled plastids using NOVOplasty. **b** Plastids assembled by mapping to a reference chloroplast genome.

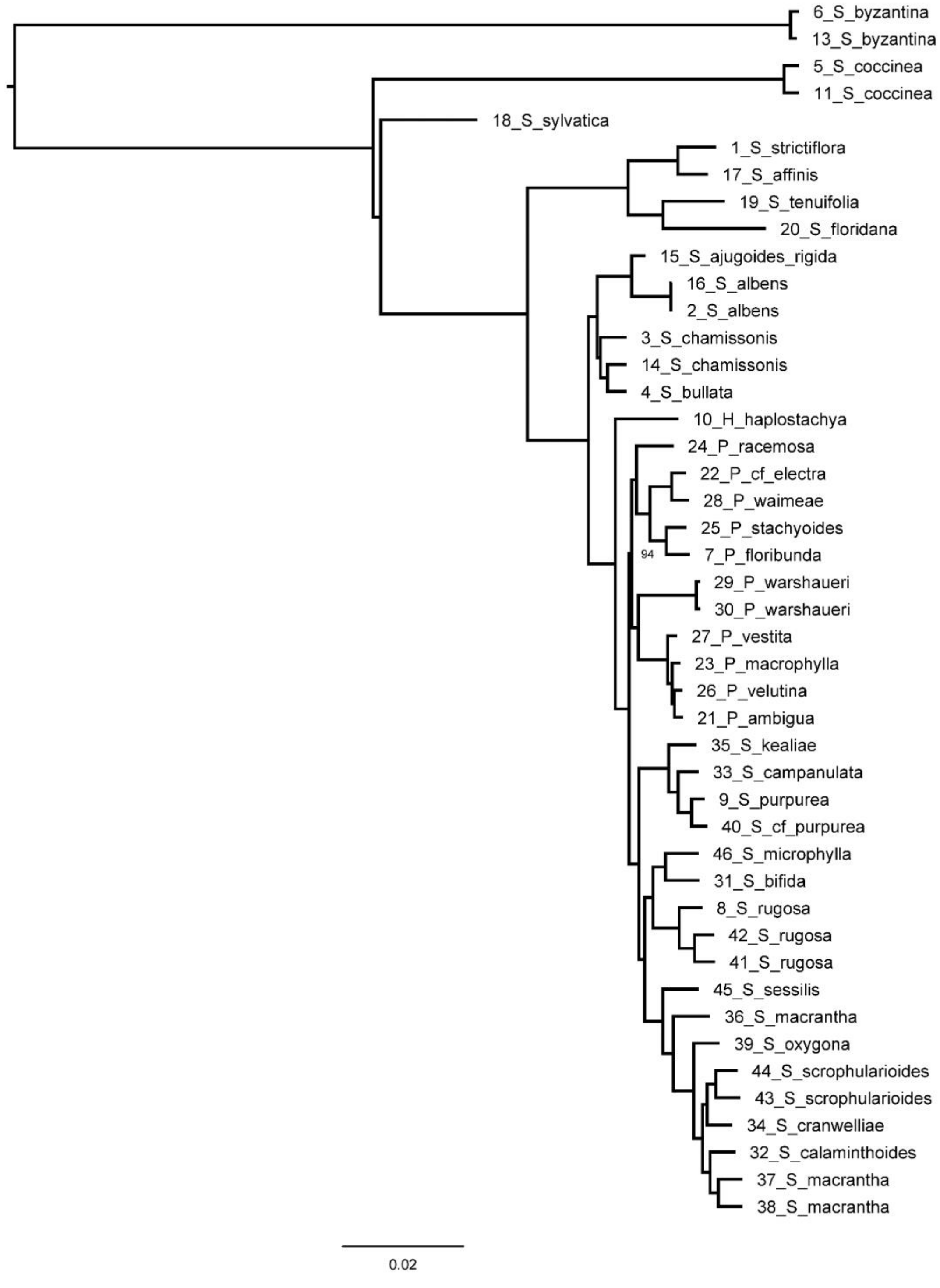

Supplementary Fig. 7: SNP phylogeny for dataset DS4. Only one bootstrap value appears below 100%, that of the very short internal branch supporting monophyly of *Phyllostegia*.

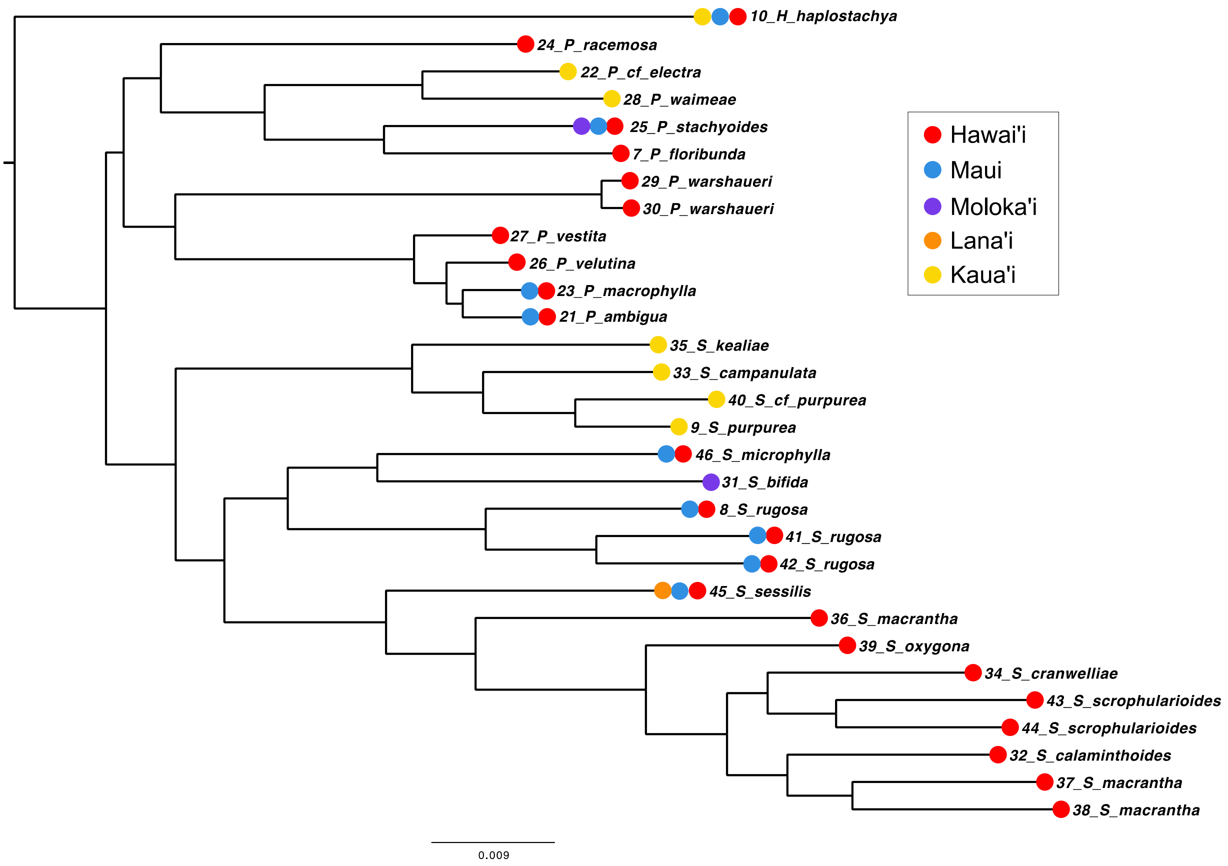

Supplementary Fig. 8: Native geographic ranges of Hawaiian mint taxa sampled for this study and shown on the SNP phylogeny of Hawaiian mints only (dataset DS4b).

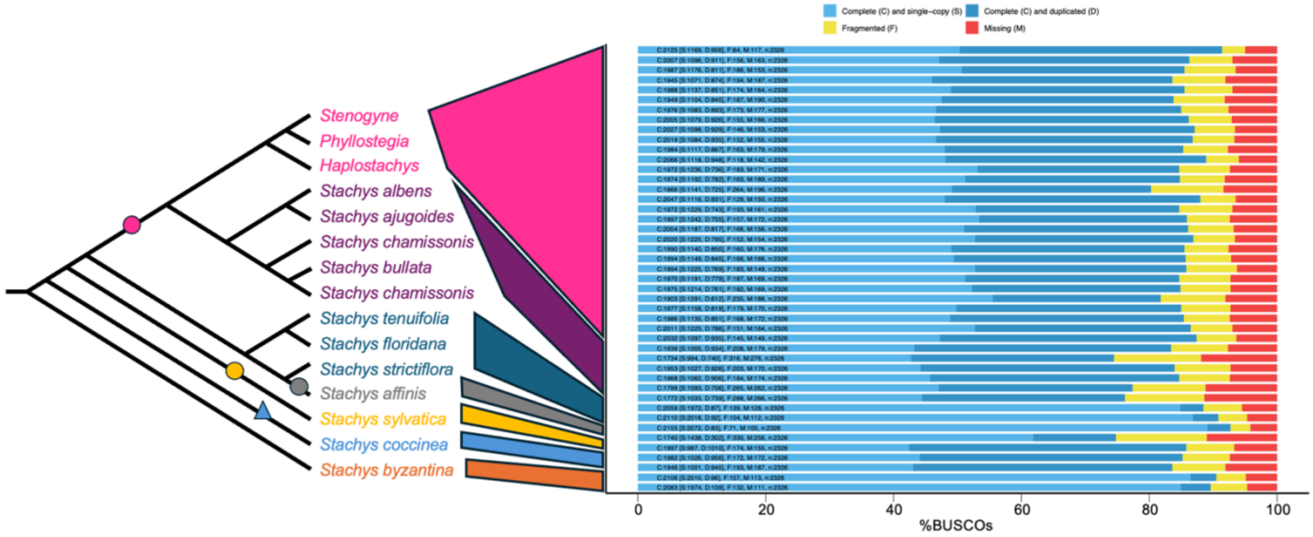

Supplementary Fig. 9: Stacked bar plot showing BUSCO single, duplicated, missing, and fragmented genes alongside a schematic of the phylogeny, with putative unique whole genome duplication events marked. See Supplementary Data 8 for details on BUSCOs.

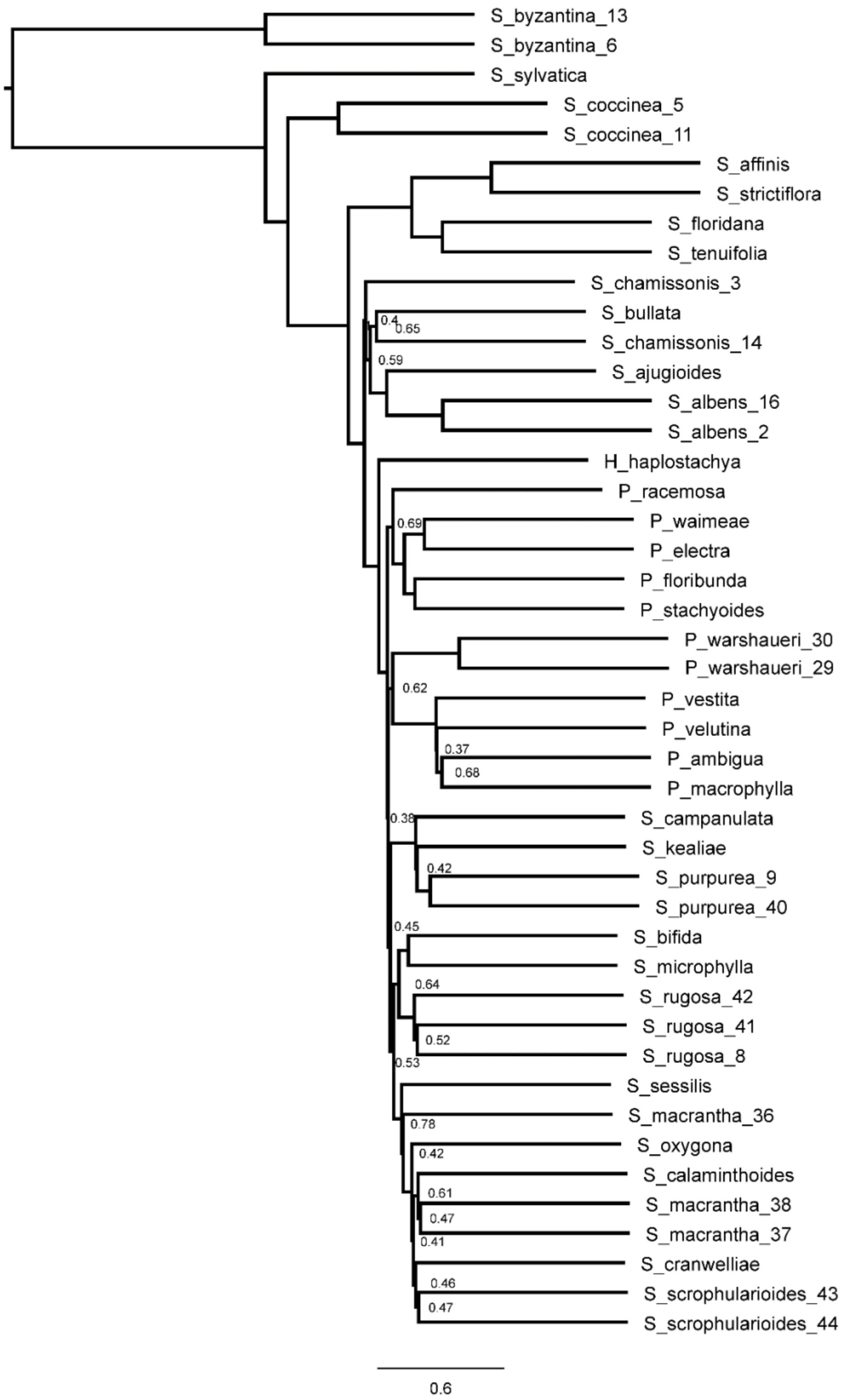

Supplementary Fig. 10: Coalescent BUSCO tree with local posterior probability, only localPP values below 0.8 are shown.

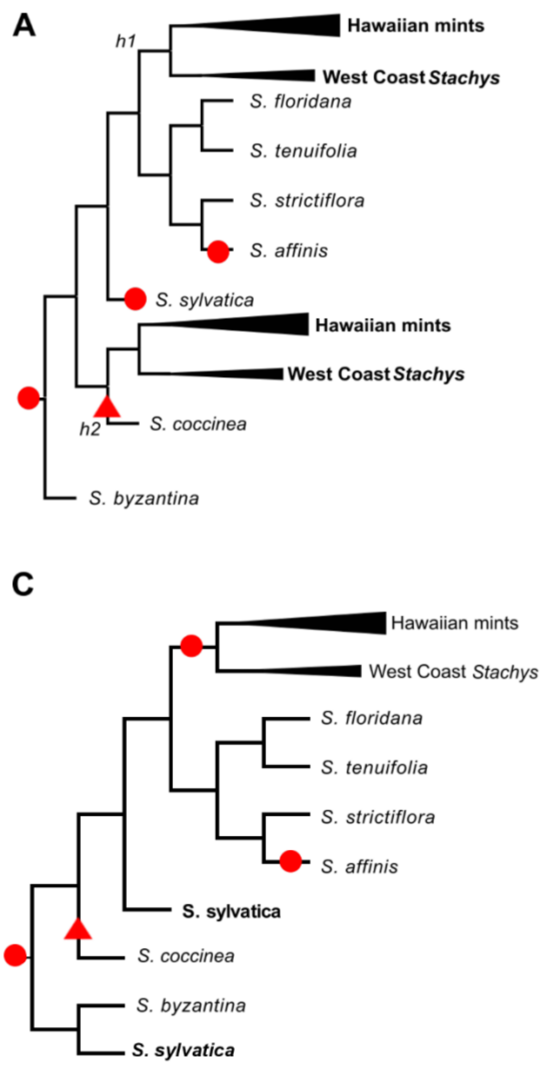

Supplementary Fig. 11: Summarized GRAMPA results for each polyploidy event among taxa samples with the putative polyploid taxa in bold and WGD events marked by a circle and WGT events marked by a triangle. **a** The WGD event at the base of West Coast *Stachys* and Hawaiian mints. **b** Polyploidy event only found in *Stachys affinis*. **c** Polyploidy event in *Stachys coccinea*. **d** Polyploidy event for *Stachys sylvatica*.

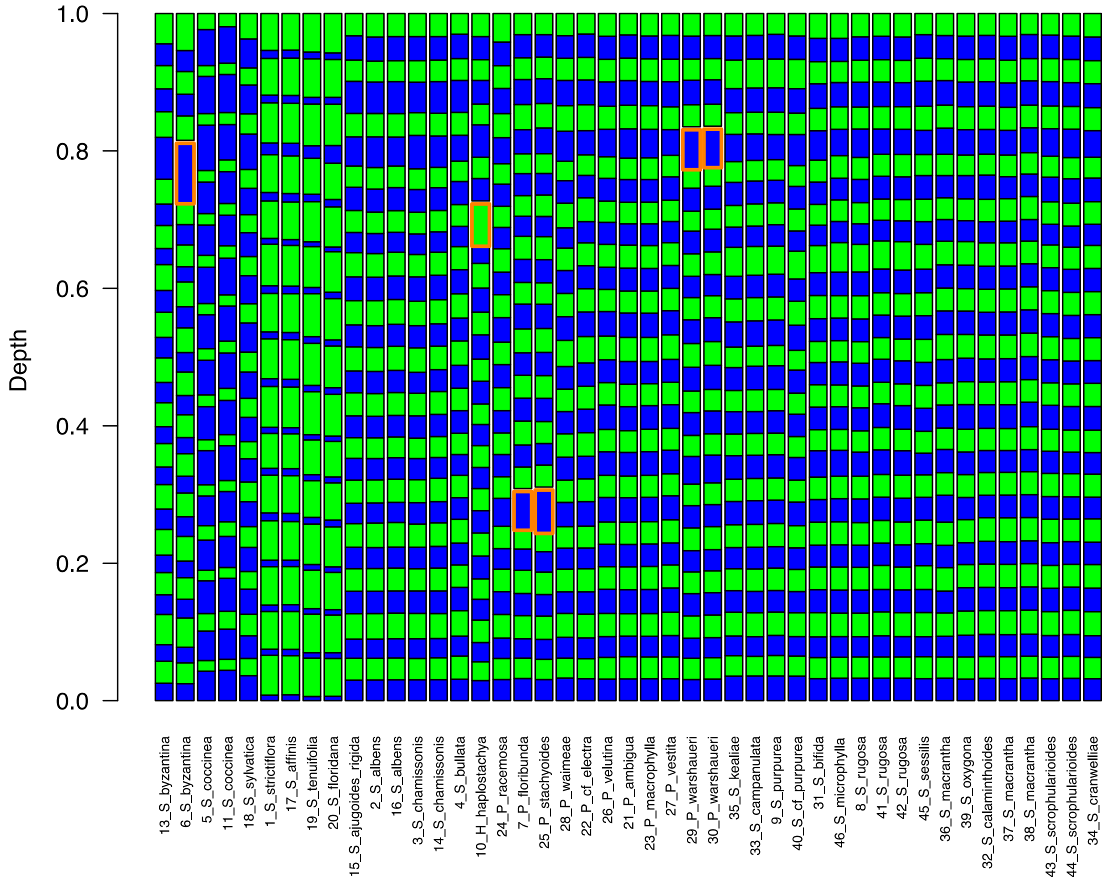

Supplementary Fig. S12: Percentage stacked bar chart showing Illumina mapping depth of each sample with chromosomes colored according to subgenome; blue represents the UC subgenome and green represents the ENAA subgenome. Chromosomes that appeared doubled have an orange outline: in *Stachys byzantina*, a UC chromosome was doubled (chr 37), in *Haplostachys haplostachya* there was doubling of an ENAA chromosome (chr 32), sister taxa *Phyllostegia. floribunda* and *P. stachyoides* shared doubling of an UC chromosome (chr 20), and the two *P. warshaueri* specimens shared doubling of a different UC chromosome (chr 37).

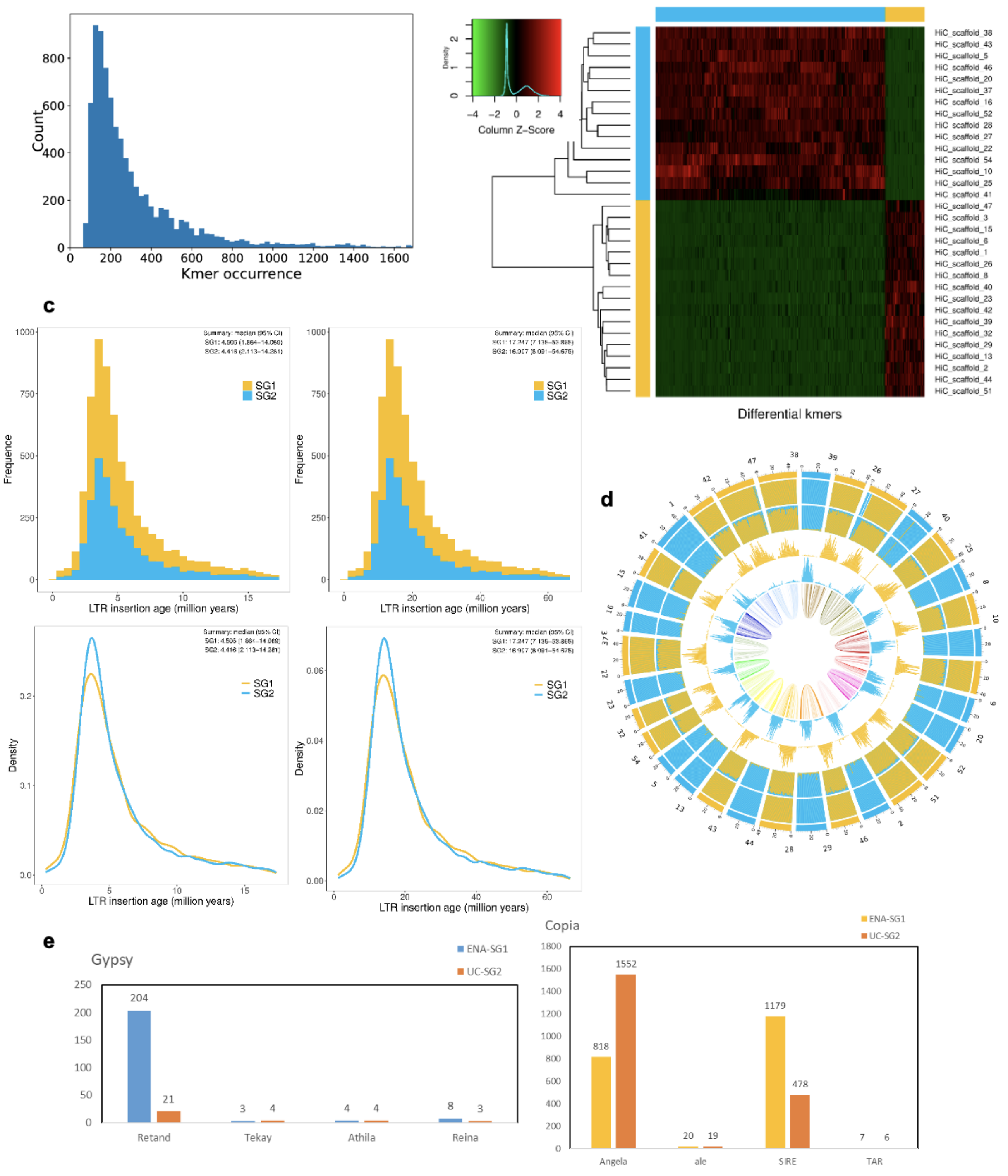

Supplementary Fig. 13: SubPhaser results, where SG1 represents the ENA subgenome and SG2 represents the UC subgenome. **a** kmer distribution in *Stenogyne calaminthoides*. **b** Matrix of differential kmers. **c** Histograms (top) and transformed density plots (bottom) for dated LTRs. Two different mutations rates were used (left to right): 6.7E-09 and 1.75E-09 mutations per site per year. **d** Subgenome Circos plot. **e** Represented LTR families in the Gypsy (left) and Copia (right) category.

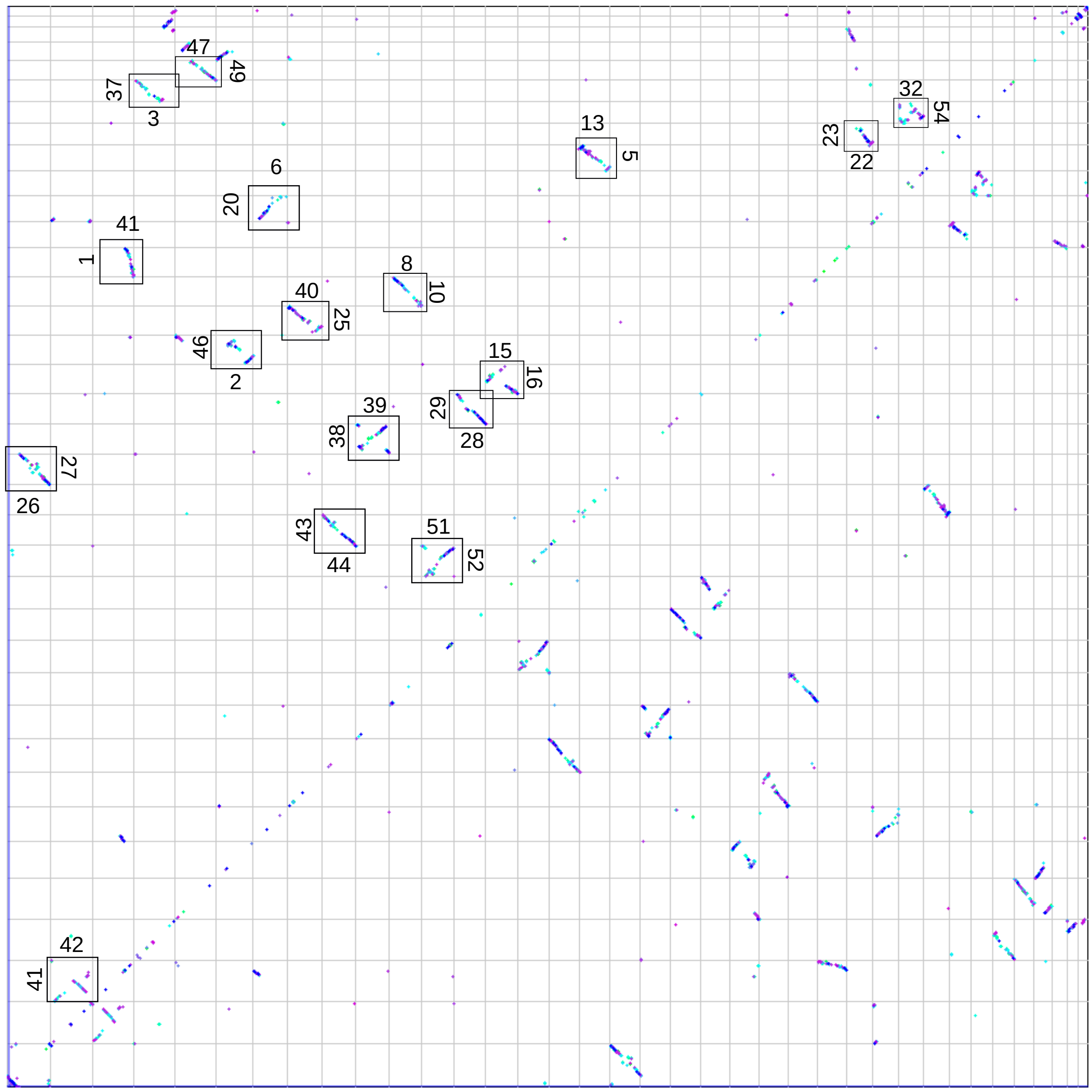

Supplementary Fig. 14: CoGe SynMap of *Stenogyne* self:self with syntenic pairs in boxes. The chromosome numbers are labelled, and grey boxes indicate pairs that were not used in further analysis.

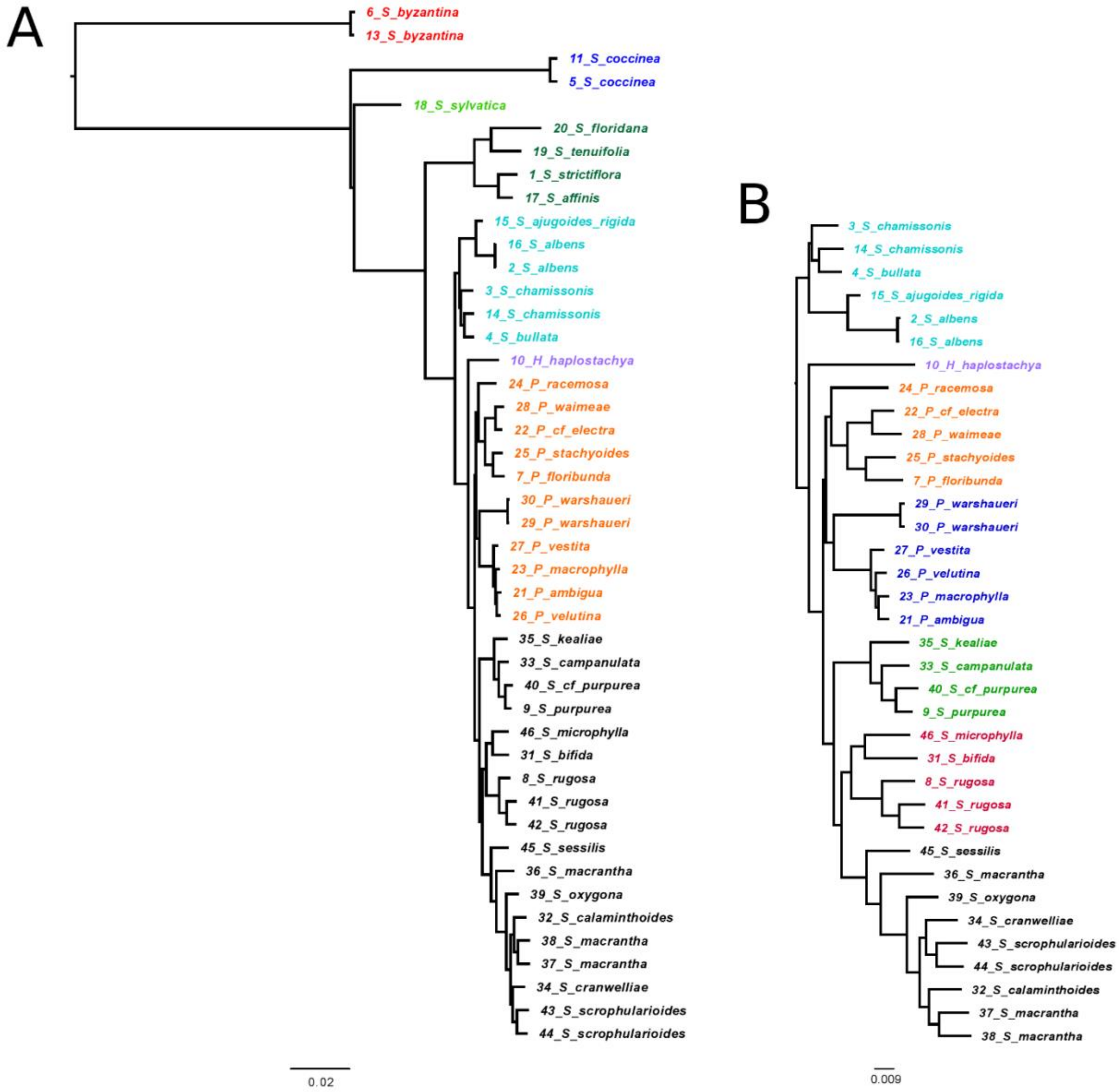

Supplementary Fig. 15: Monophyletic groupings used for *Twisst* analyses. **a** Groupings used for *Twisst* used for the entire dataset shown on the SNP tree of dataset DS4. **b** Groupings used for Hawaiian mints and their WNA *Stachys* relatives shown on the SNP tree based on dataset DS4a.

Fig. S16a Twisst ENAA 15 chrs

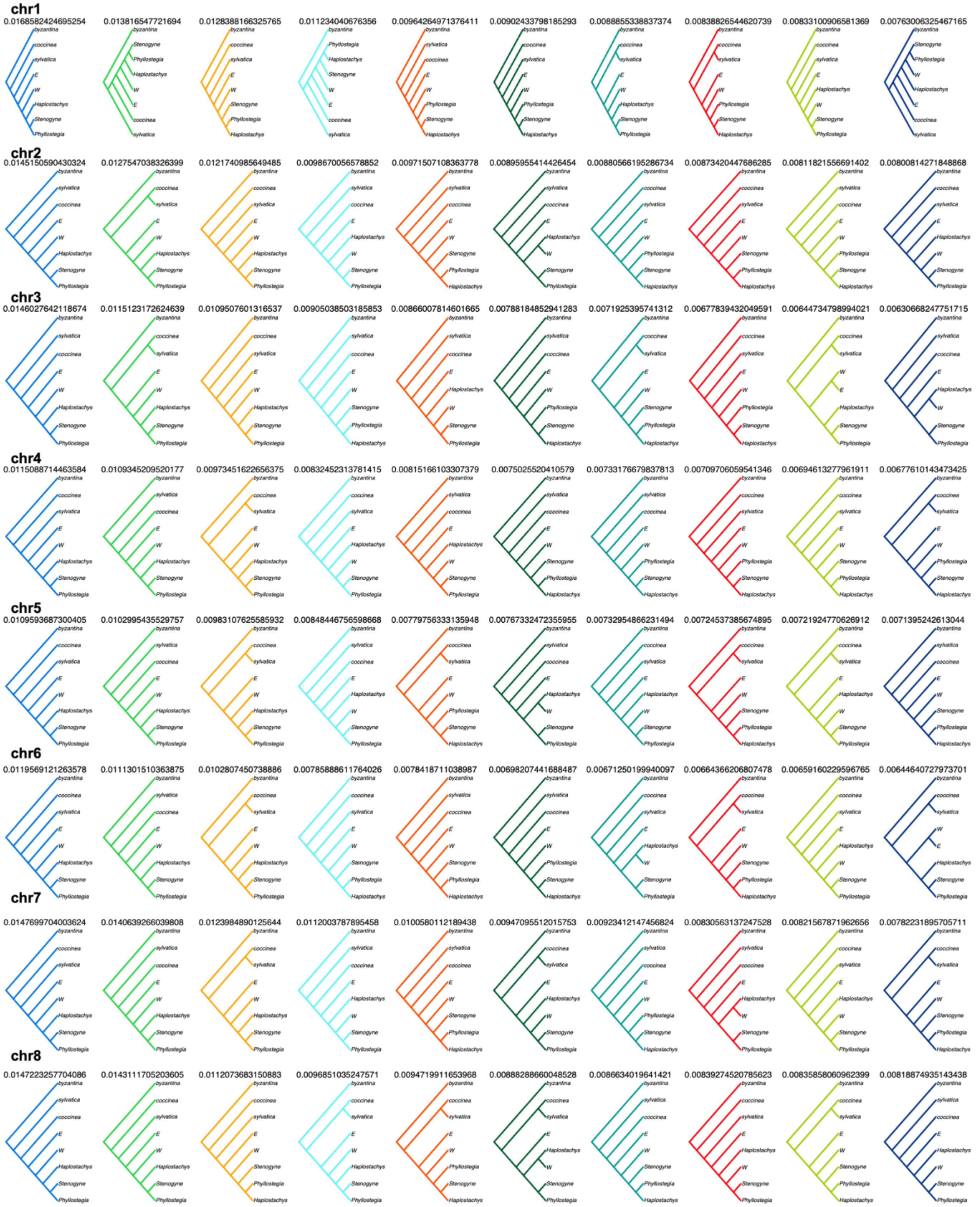

Fig. S16a

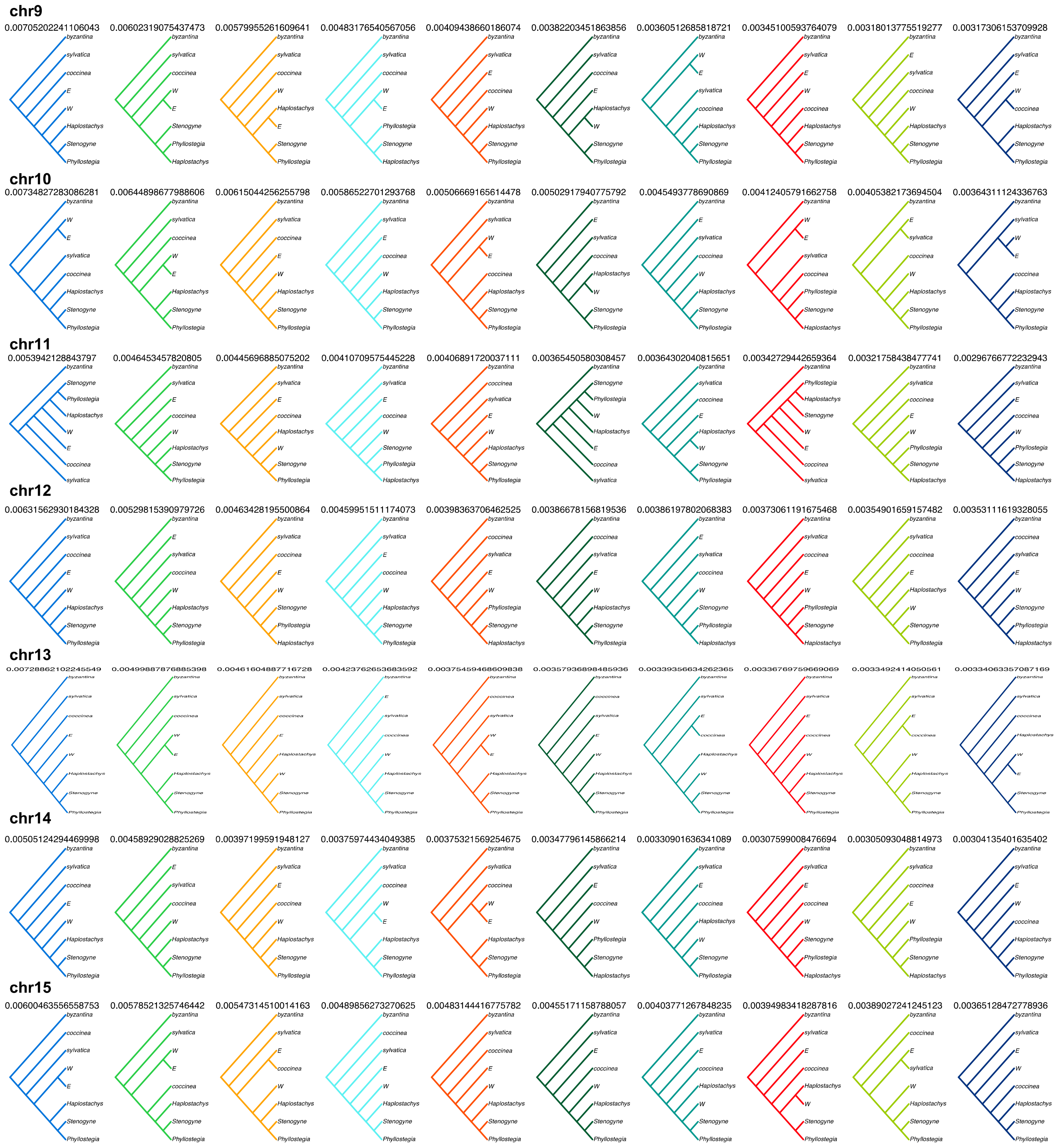
chr9
0.00705202241106043 0.00602319075437473 0.00579955261609641 0.00483176540567056 0.00409438660186074 0.00382203451863856 0.00360512685818721 0.00345100593764079 0.00318013775519277 0.00317306153709928
chr10
0.00734827283086281 0.00644898677988606 0.00615044256255798 0.00586522701293768 0.00506669165614478 0.00502917940775792 0.0045493778690869 0.00412405791662758 0.00405382173694504 0.00364311124336763
chr11
0.0053942128843797 0.0046453457820805 0.00445696885075202 0.00410709575445228 0.00406891720037111 0.00365450580308457 0.00364302040815651 0.00342729442659364 0.00321758438477741 0.00296766772232943
chr12
0.00631562930184328 0.00529815390979726 0.00463428195500864 0.00459951511174073 0.00398363706462525 0.00386678156819536 0.00386197802068383 0.00373061191675468 0.00354901659157482 0.00353111619328055
chr13
0.00728862102245549 0.00499887876885398 0.00461604887716728 0.00423762653683592 0.00375459468609838 0.00357936898485936 0.00339356634262365 0.00336769759669069 0.00334924140505661 0.00334063357087169
chr14
0.00505124294469998 0.00458929028825269 0.00397199591948127 0.00375974434049385 0.00375321569254675 0.00347796145866214 0.00330901636341089 0.00307599008476694 0.00305093048814973 0.00304135401635402
chr15
0.00600463556558753 0.00578521325746442 0.00547314510014163 0.00489856273270625 0.00483144416775782 0.00455171158788057 0.00403771267848235 0.00394983418287816 0.00389027241245123 0.00365128472778936
byzantina
sylvatica
coccinea
E
W
Haplostachys
Stenogyne
Phyllostegia

Fig. S16b Twisst UC 15 chrs

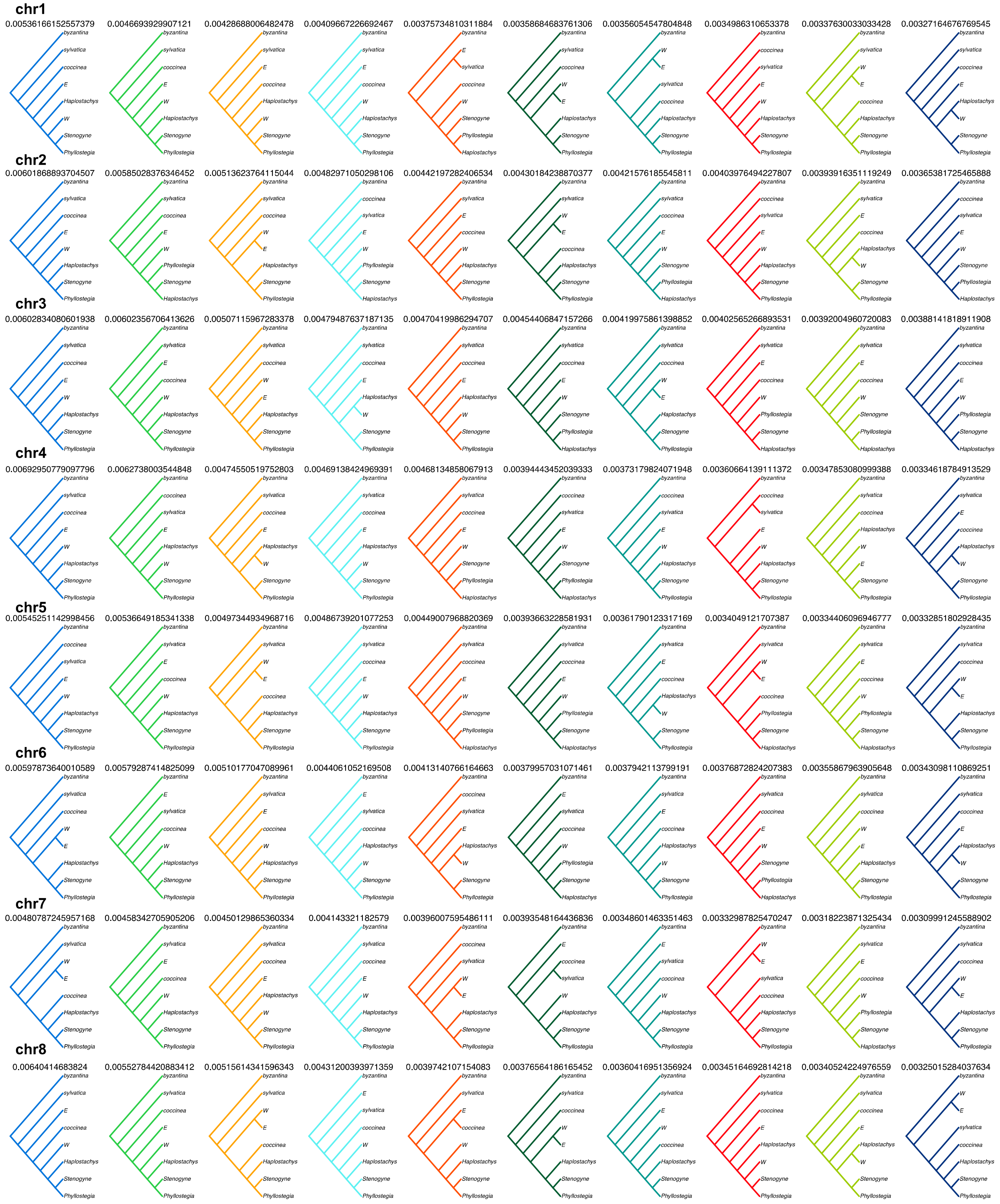

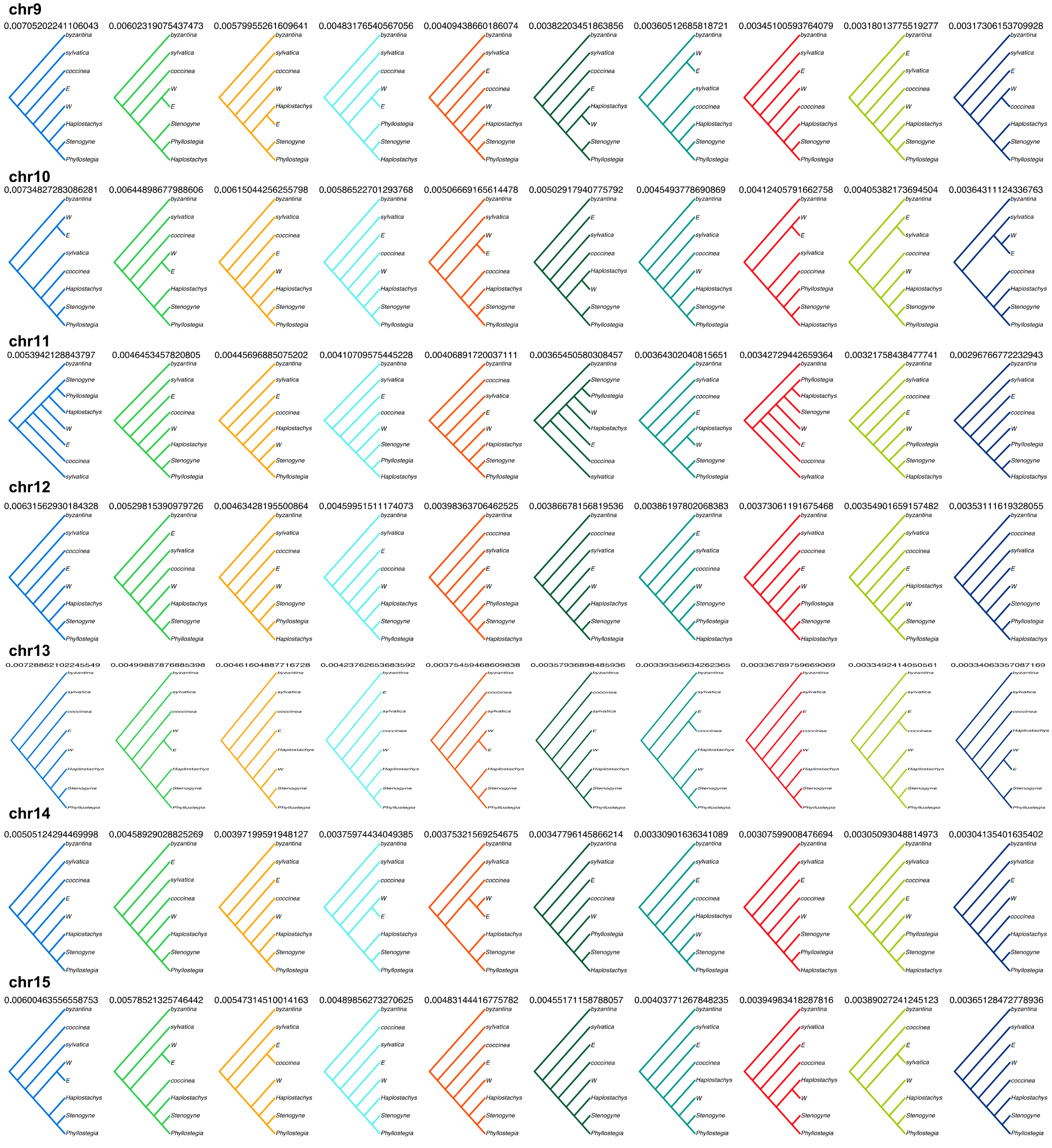

Supplementary Fig. 16: Twisst results plotted according to chromosome assigned to each of the ENAA and UC subgenomes. **a** ENAA chromosomes **b** UC chromosomes.

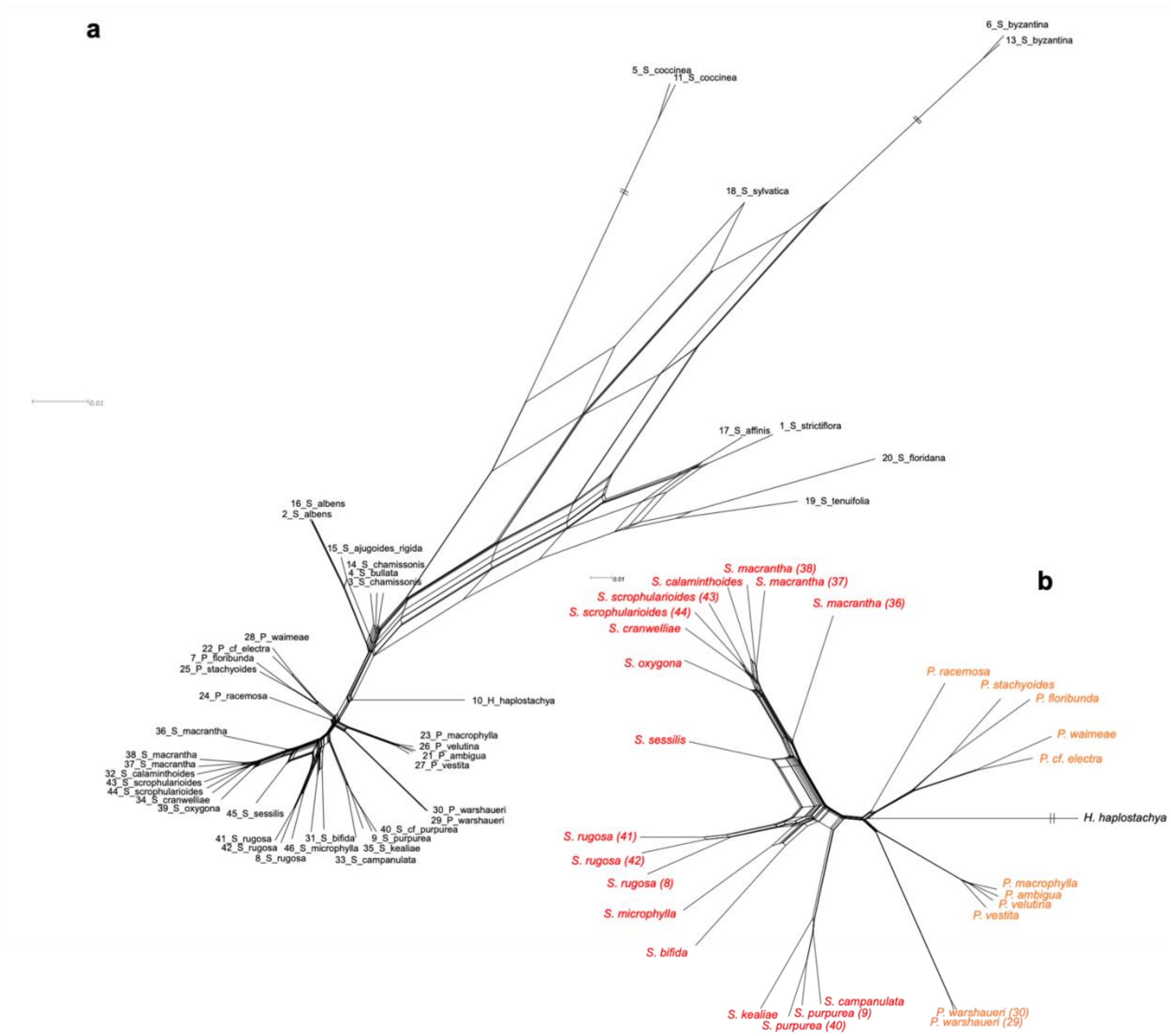

Supplementary Fig. 17: Phylogenetic network of the Hawaiian mints and relatives. **a** NeighborNet of all samples (dataset DS4). **b** NeighborNet of Hawaiian mints only (dataset DS4b).

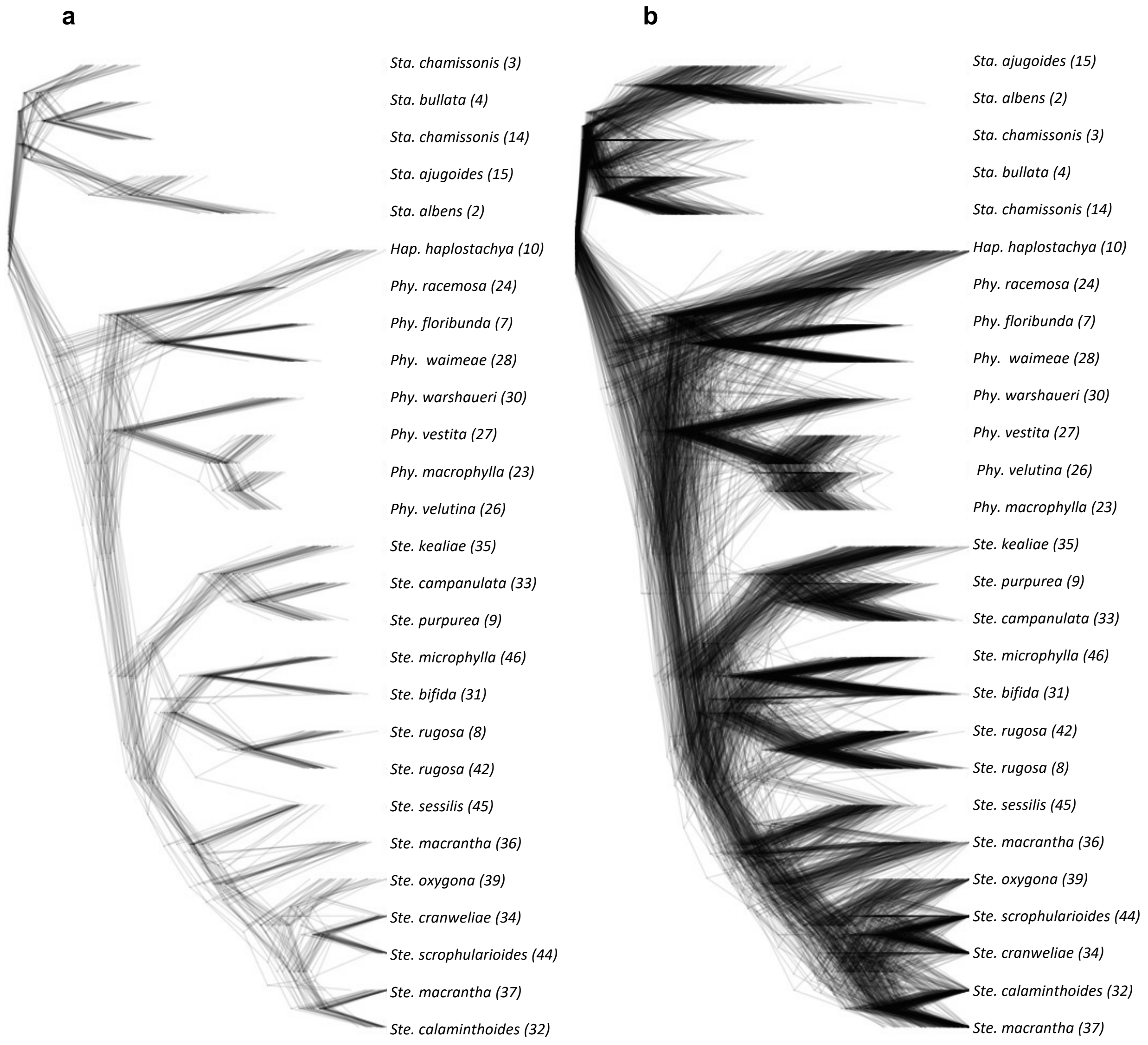

Supplementary Fig. 18: Phylogenetic incongruence displayed with DensiTree. **a** DensiTree based on chromosomes (left). **b** DensiTree based on 287 trees made from nonoverlapping windows of 25,000 SNPs (right).

Fig. S19a PCA DS4

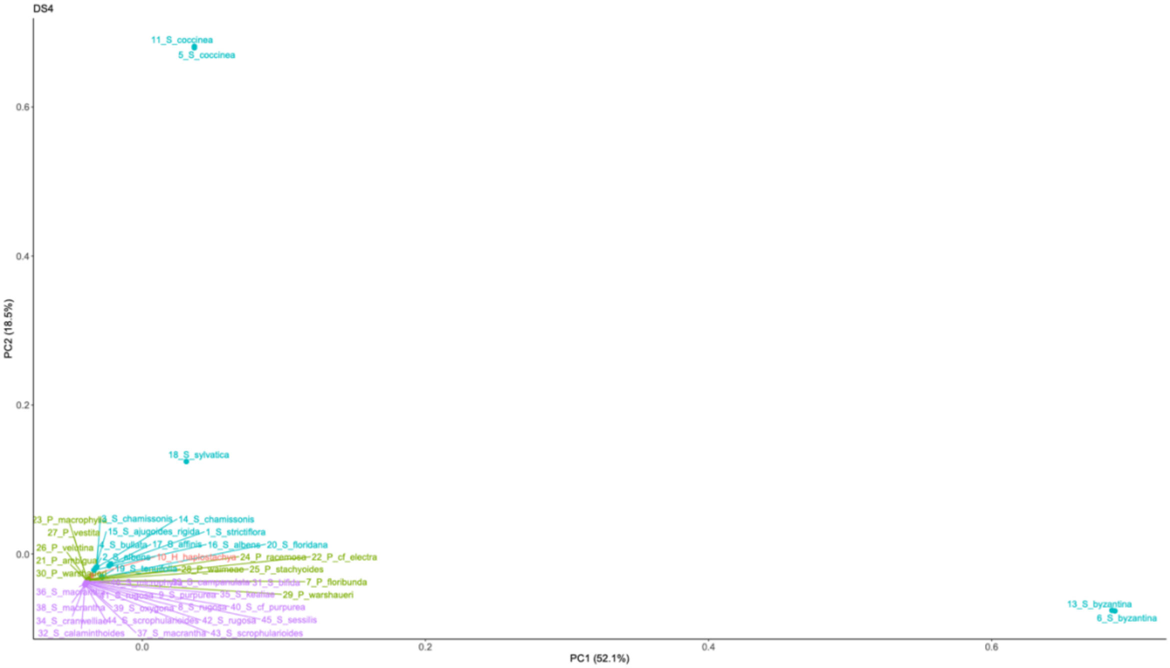

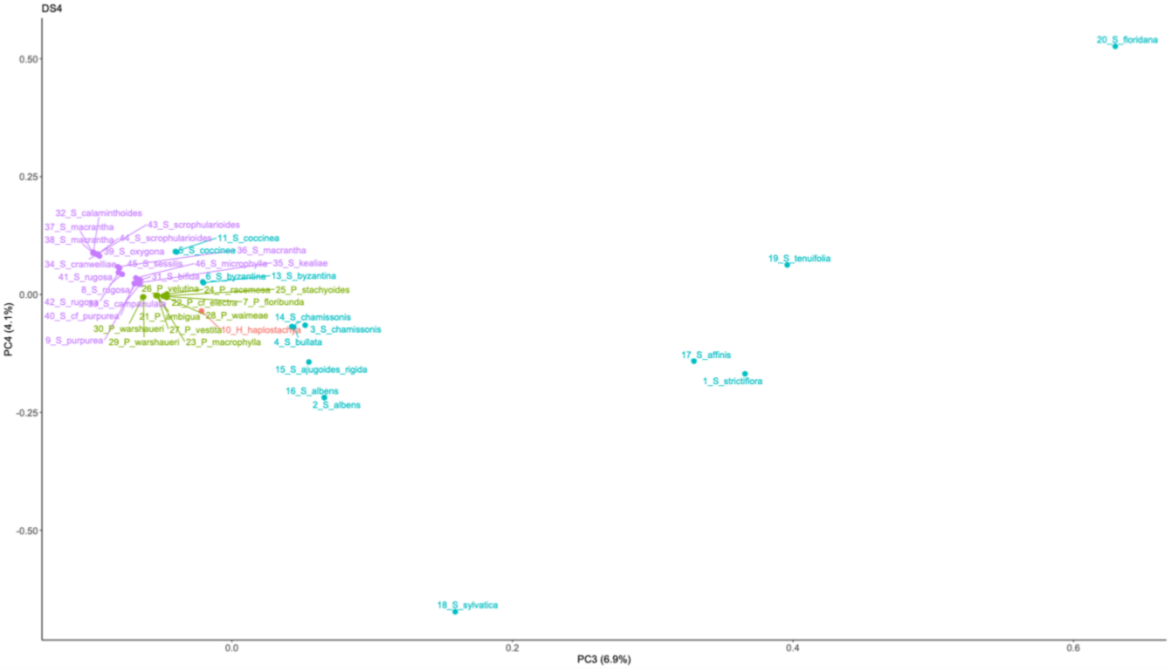

Fig. S19a

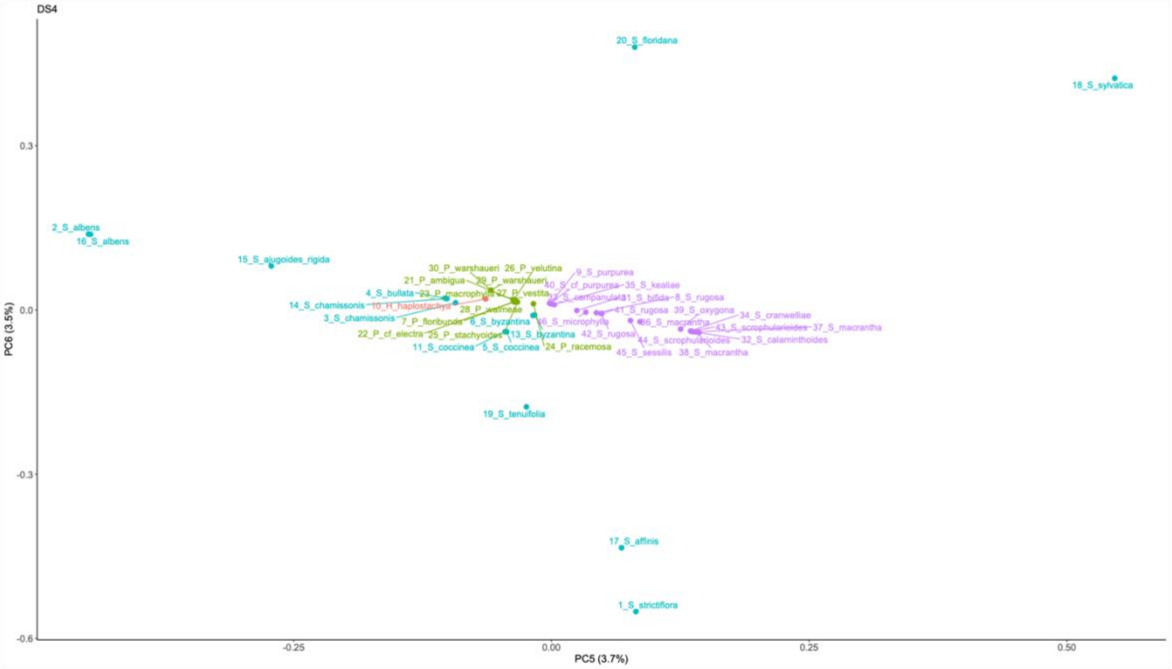
DS4
20_S_floridana
18_S_sylvatica
2_S_albens
16_S_albens
15_S_ajugoides_rigida
30_P_warshaueri
26_P_velutina
21_P_ambigua
29_P_warshaueri
4_S_bullata
23_P_macrophylla
27_P_vestita
14_S_chamissonis
10_H_haplostachya
28_P_waimeae
3_S_chamissonis
7_P_floribunda
6_S_byzantina
22_P_cf_electra
25_P_stachyoides
13_S_byzantina
11_S_coccinea
5_S_coccinea
24_P_racemosa
9_S_purpurea
40_S_cf_purpurea
35_S_kealiae
31_S_bifida
8_S_rugosa
41_S_rugosa
39_S_oxygona
34_S_cranwelliae
46_S_microphylla
36_S_macrantha
43_S_scrophularioides
37_S_macrantha
42_S_rugosa
44_S_scrophularioides
32_S_calaminthoides
45_S_sessilis
38_S_macrantha
19_S_tenuifolia
17_S_affinis
1_S_strictiflora
0.3
0.0
-0.3
-0.6
PC6 (3.5%)
-0.25
0.00
0.25
0.50
PC5 (3.7%)

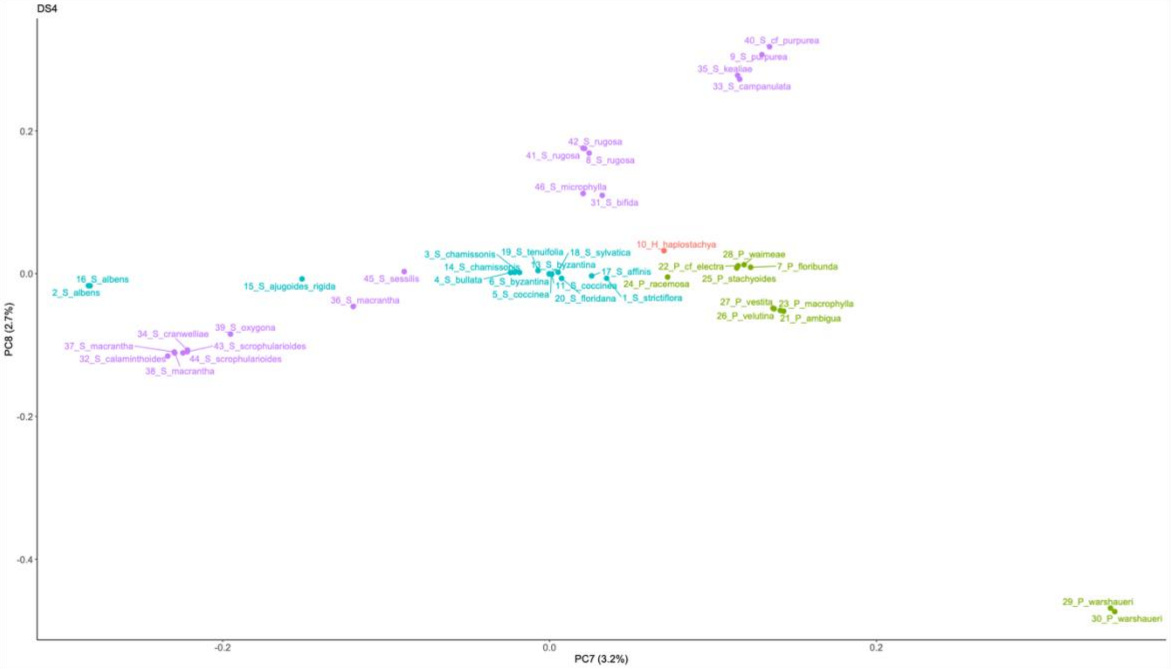
DS4
40_S_cf_purpurea
9_S_purpurea
35_S_kealiae
33_S_campanulata
42_S_rugosa
41_S_rugosa
8_S_rugosa
46_S_microphylla
31_S_bifida
10_H_haplostachya
3_S_chamissonis
19_S_tenuifolia
18_S_sylvatica
28_P_waimeae
14_S_chamissonis
13_S_byzantina
22_P_cf_electra
7_P_floribunda
17_S_affinis
25_P_stachyoides
16_S_albens
2_S_albens
15_S_ajugoides_rigida
45_S_sessilis
4_S_bullata
6_S_byzantina
11_S_coccinea
24_P_racemosa
5_S_coccinea
20_S_floridana
1_S_strictiflora
36_S_macrantha
27_P_vestita
23_P_macrophylla
26_P_velutina
21_P_ambigua
34_S_cranwelliae
39_S_oxygona
37_S_macrantha
43_S_scrophularioides
32_S_calaminthoides
44_S_scrophularioides
38_S_macrantha
29_P_warshaueri
30_P_warshaueri
0.2
0.0
-0.2
-0.4
PC8 (2.7%)
-0.2
0.0
0.2
PC7 (3.2%)

Fig. S19b PCA DS4c

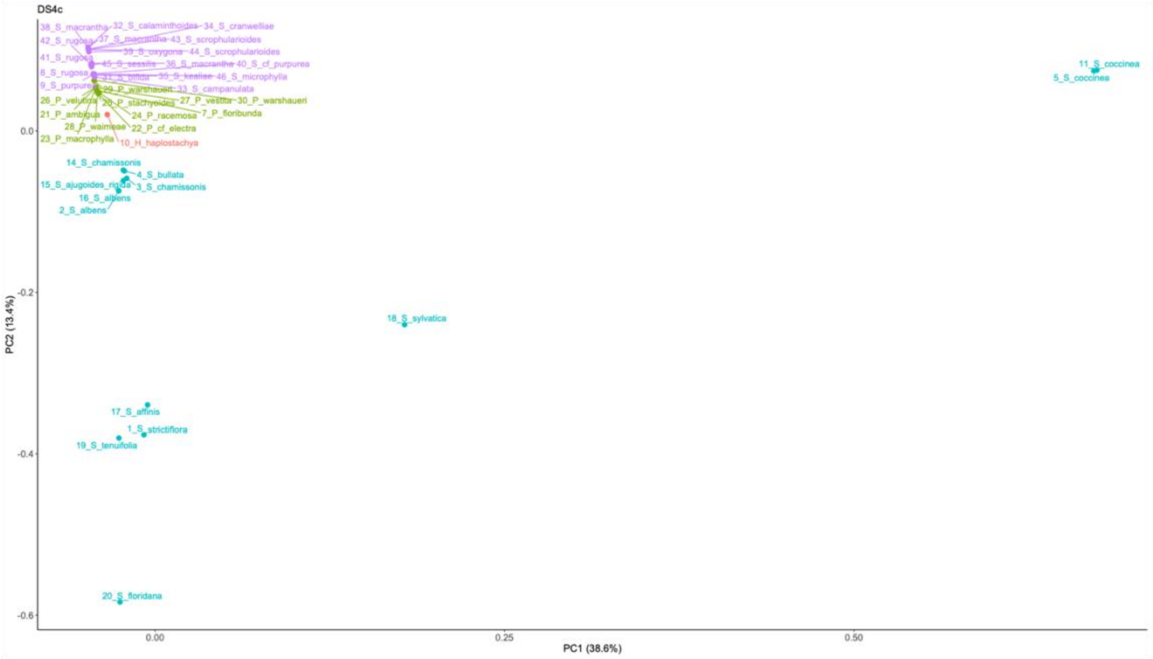

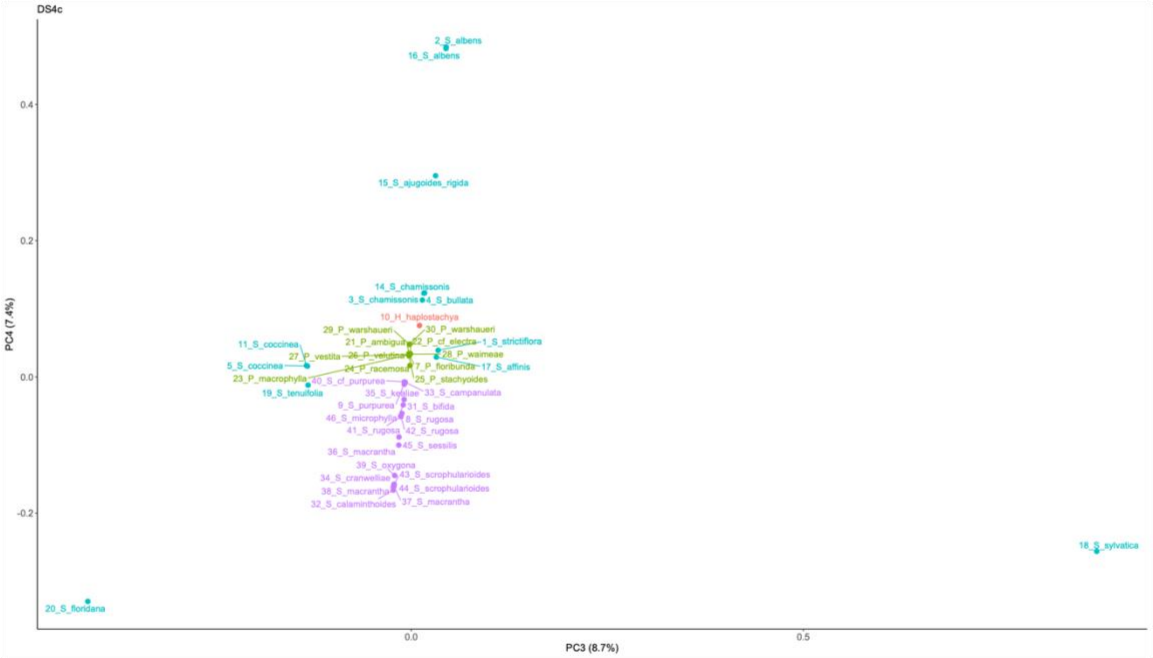

Fig. S19b

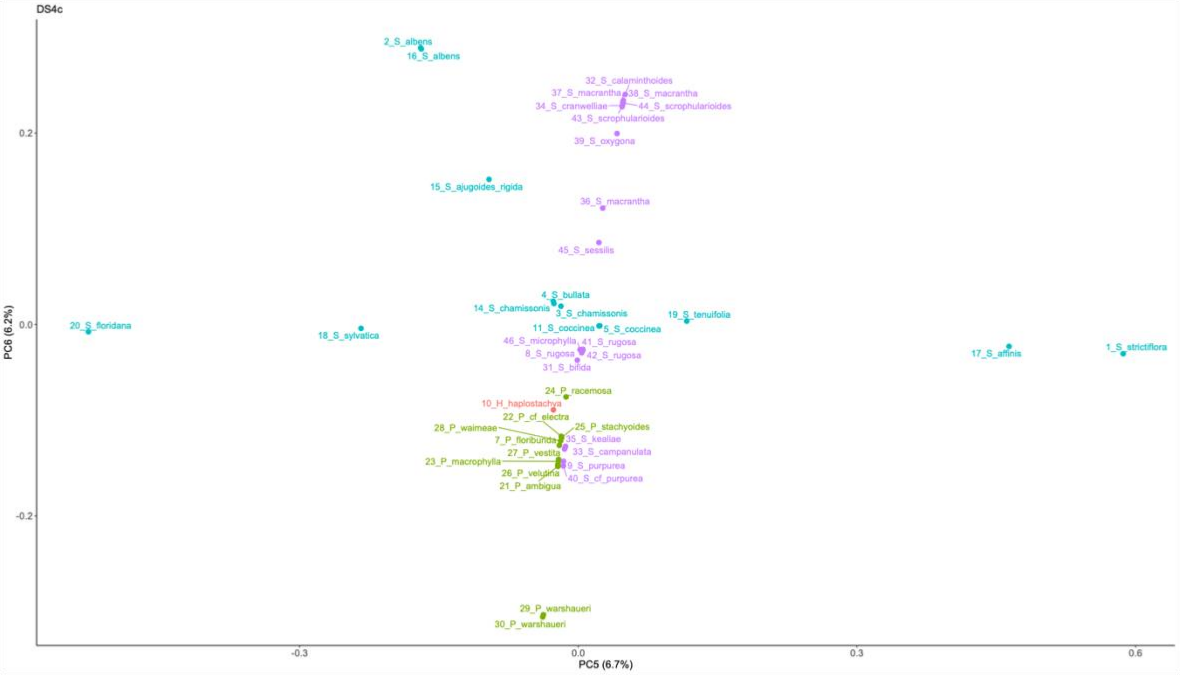
DS4c
PC6 (6.2%)
PC5 (6.7%)
0.2
0.0
-0.2
-0.3
0.0
0.3
0.6
2_S_albens
16_S_albens
32_S_calaminthoides
37_S_macrantha
38_S_macrantha
34_S_cranwelliae
44_S_scrophularioides
43_S_scrophularioides
39_S_oxygona
15_S_ajugoides_rigida
36_S_macrantha
45_S_sessilis
4_S_bullata
14_S_chamissonis
3_S_chamissonis
19_S_tenuifolia
20_S_floridana
18_S_sylvatica
11_S_coccinea
5_S_coccinea
46_S_microphylla
41_S_rugosa
8_S_rugosa
42_S_rugosa
31_S_bifida
17_S_affinis
1_S_strictiflora
24_P_racemosa
10_H_haplostachya
22_P_cf_electra
28_P_waimeae
25_P_stachyoides
7_P_floribunda
35_S_kealiae
27_P_vestita
33_S_campanulata
23_P_macrophylla
9_S_purpurea
26_P_velutina
40_S_cf_purpurea
21_P_ambigua
29_P_warshaueri
30_P_warshaueri

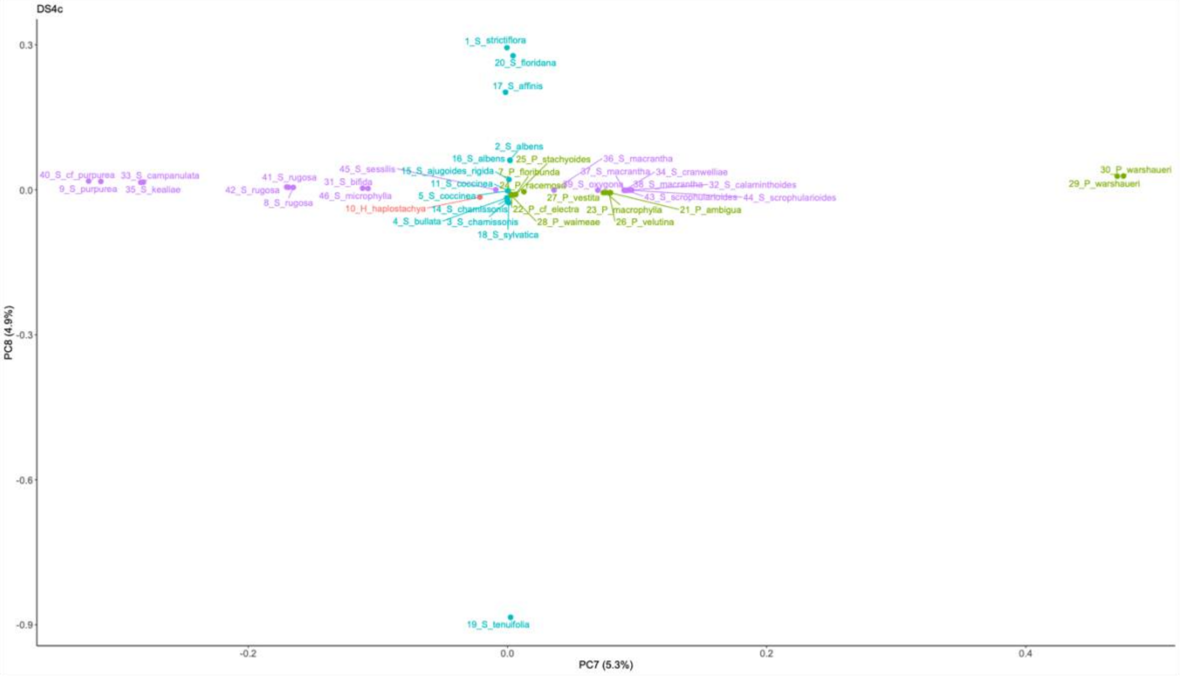
DS4c
PC8 (4.9%)
PC7 (5.3%)
0.3
0.0
-0.3
-0.6
-0.9
-0.2
0.0
0.2
0.4
1_S_strictiflora
20_S_floridana
17_S_affinis
2_S_albens
16_S_albens
25_P_stachyoides
36_S_macrantha
40_S_cf_purpurea
33_S_campanulata
9_S_purpurea
35_S_kealiae
41_S_rugosa
42_S_rugosa
8_S_rugosa
31_S_bifida
46_S_microphylla
45_S_sessilis
15_S_ajugoides_rigida
7_P_floribunda
37_S_macrantha
34_S_cranwelliae
11_S_coccinea
24_P_racemosa
39_S_oxygona
38_S_macrantha
32_S_calaminthoides
5_S_coccinea
27_P_vestita
43_S_scrophularioides
44_S_scrophularioides
10_H_haplostachya
14_S_chamissonis
22_P_cf_electra
23_P_macrophylla
21_P_ambigua
4_S_bullata
3_S_chamissonis
28_P_waimeae
26_P_velutina
18_S_sylvatica
30_P_warshaueri
29_P_warshaueri
19_S_tenuifolia

Fig. S19c PCA DS4b

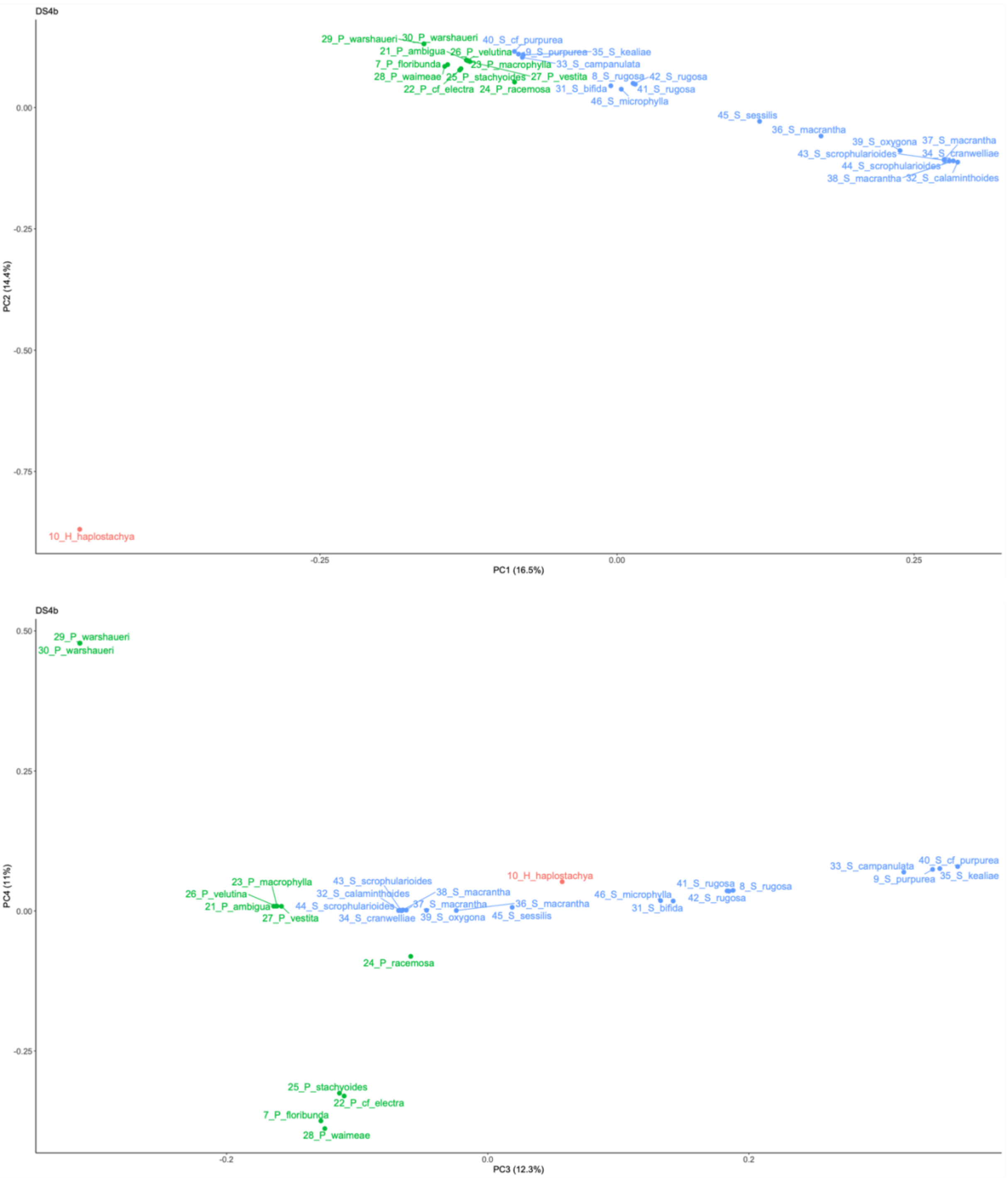

Fig. S19c

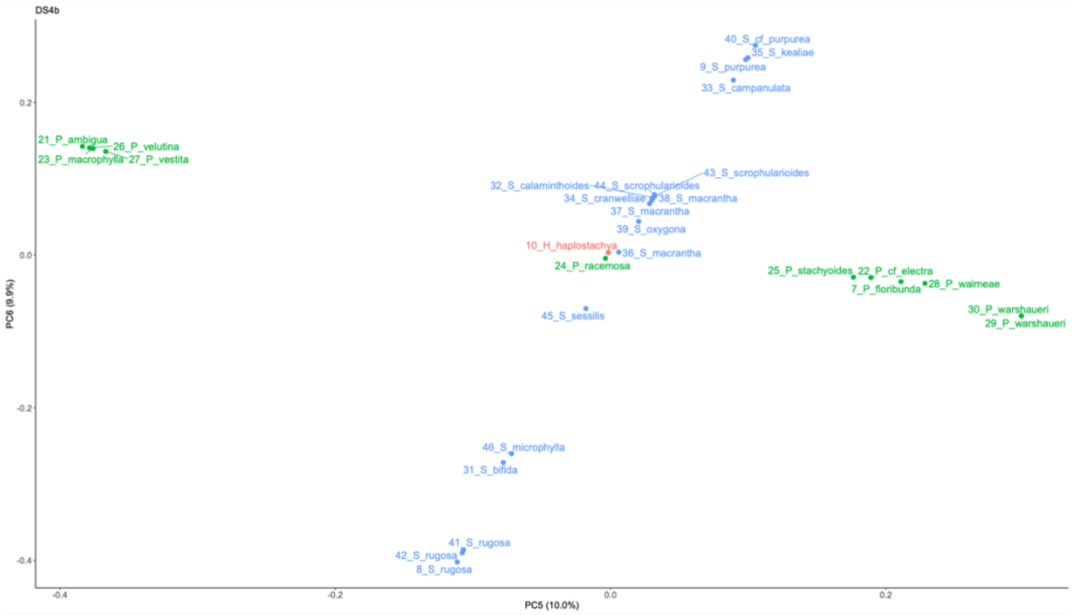

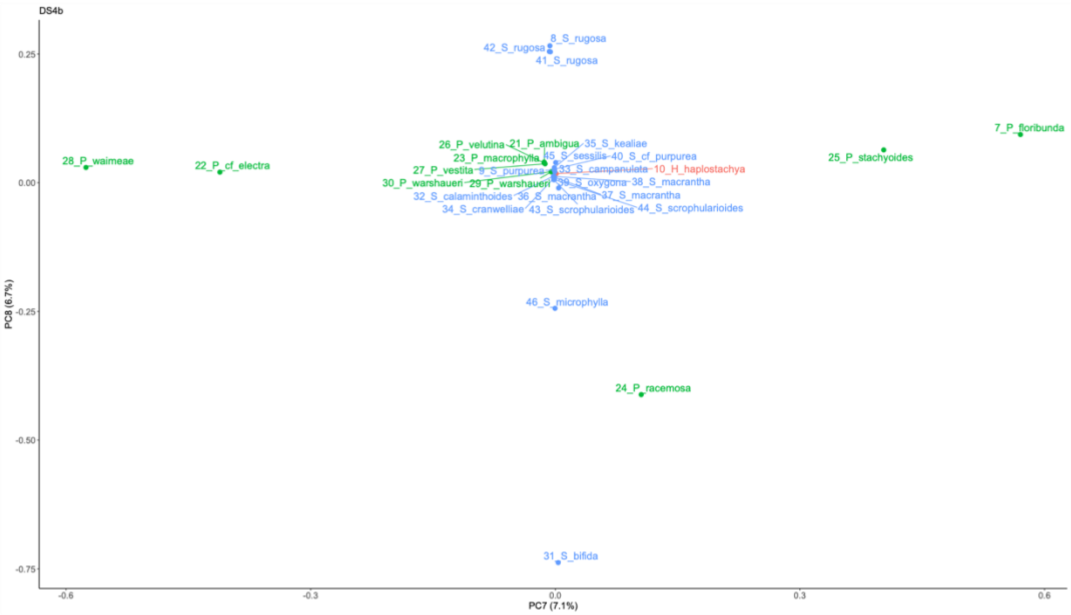

Supplementary Fig. 19: Principal component analysis based on SNP data. **a** PC1 through PC8 for the dataset including all individuals (dataset DS4). **b** PC1 through PC8 for all samples, excluding *Stachys byzantina* (dataset DS4c). **c** PC1 through PC8 for Hawaiian mint samples only (dataset DS4b).

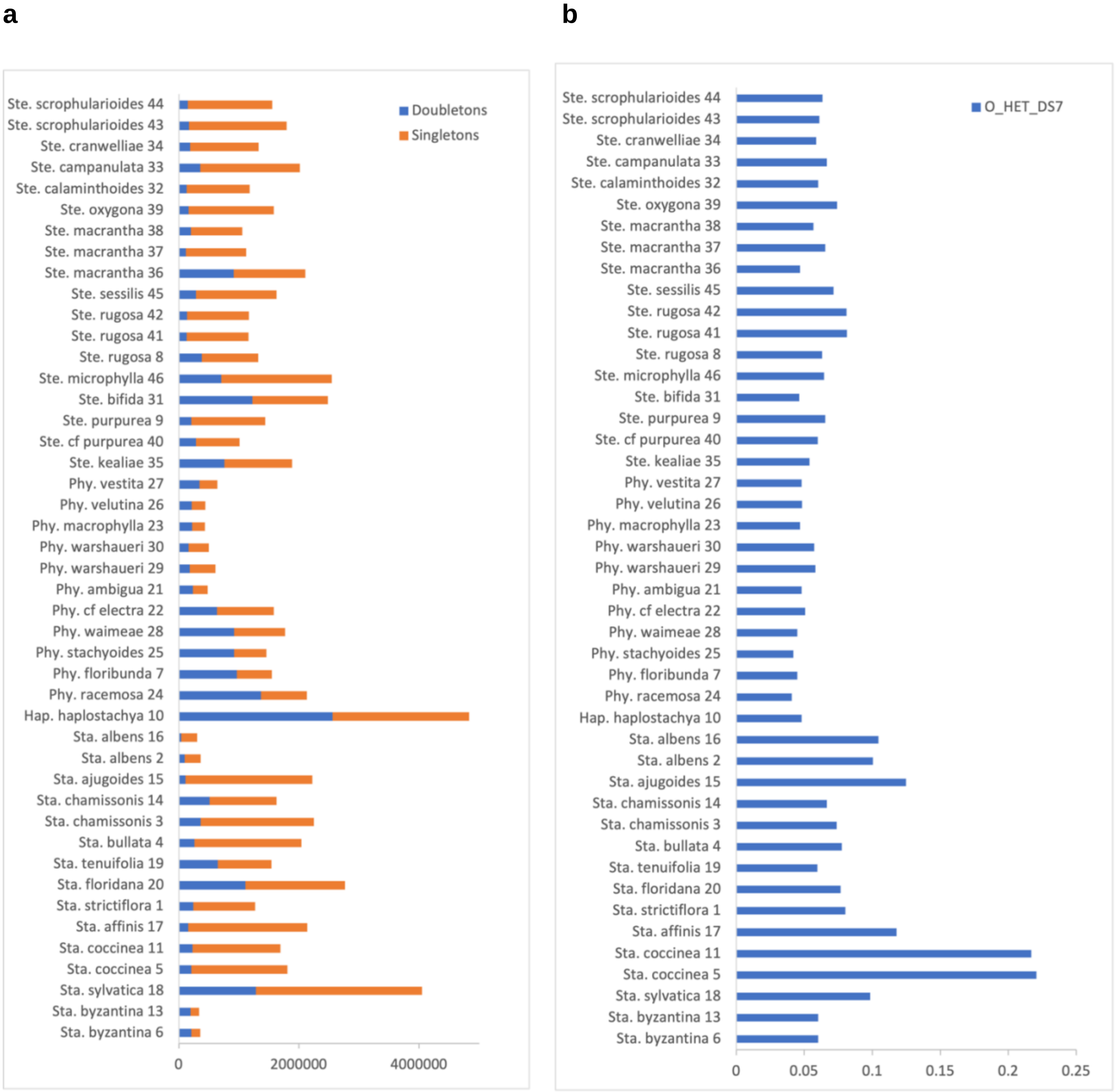

Supplementary Fig. 20: Genetic diversity among the Hawaiian mints and relatives. **a** Stacked barplot of number of singletons and doubletons as calculated by vcftools for each sample. **b** Heterozygosity based on SNP dataset DS7 (Supplementary Table 6).

Fig. S21

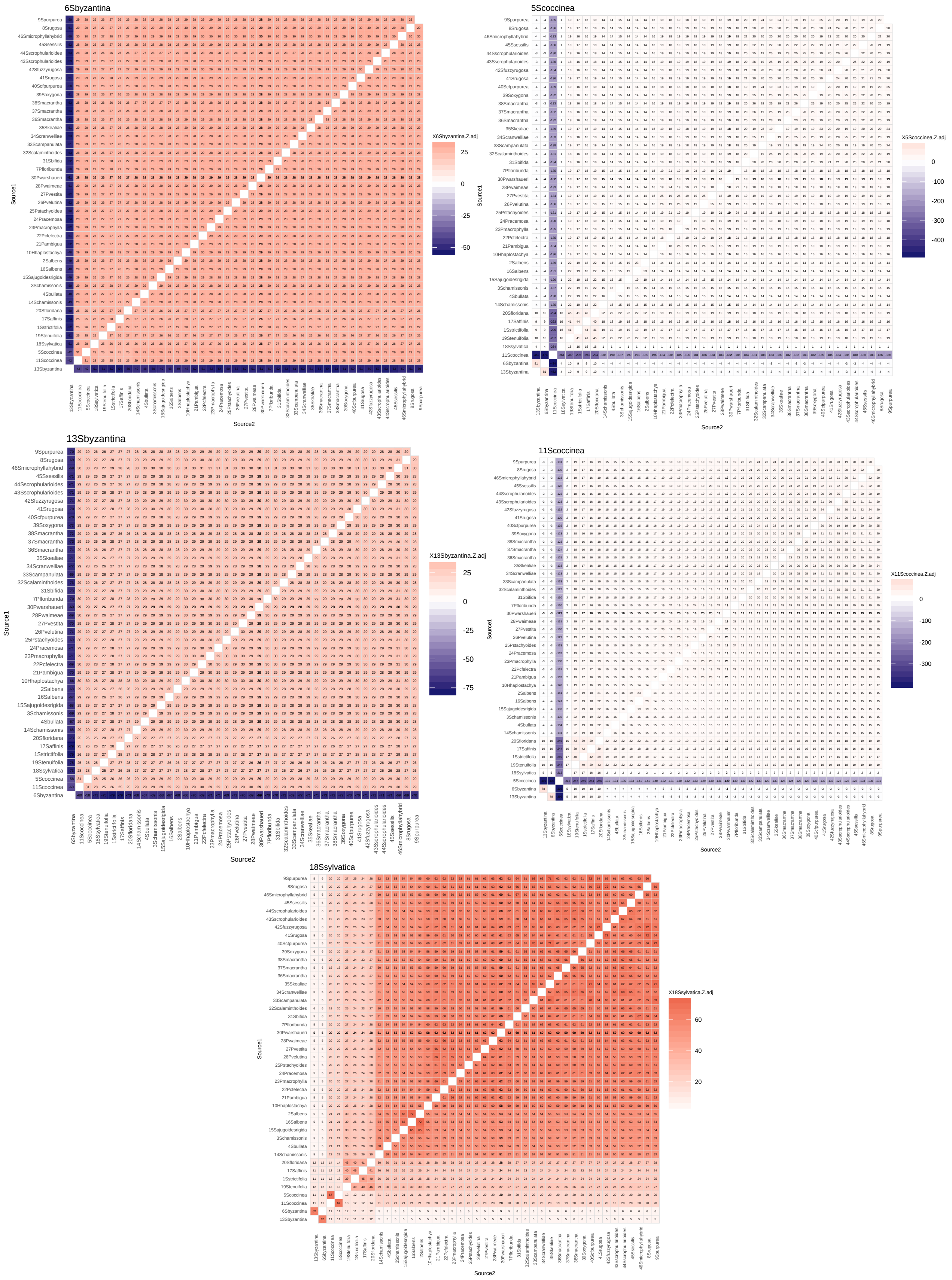
6Sbyzantina
X6Sbyzantina.Z.adj
25
0
-25
-50
Source1
Source2
5Scoccinea
X5Scoccinea.Z.adj
0
-100
-200
-300
-400
Source1
Source2
13Sbyzantina
X13Sbyzantina.Z.adj
25
0
-25
-50
-75
Source1
Source2
11Scoccinea
X11Scoccinea.Z.adj
0
-100
-200
-300
Source1
Source2
18Ssylvatica
X18Ssylvatica.Z.adj
60
40
20
Source1
Source2

Fig. S21

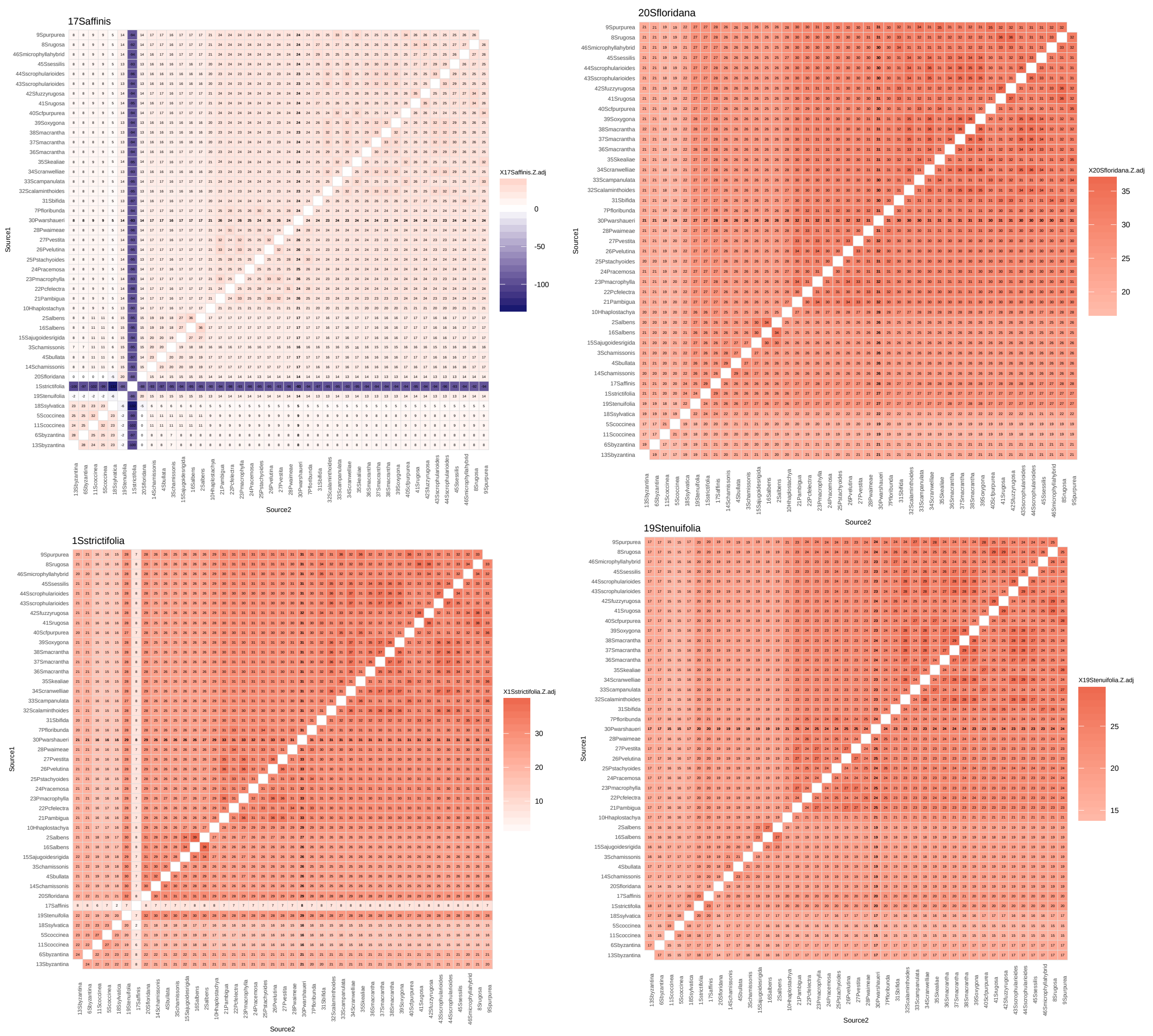

17Saffinis
X17Saffinis.Z.adj
0
-50
-100
Source1
Source2
20Sfloridana
X20Sfloridana.Z.adj
35
30
25
20
Source1
Source2
1Sstrictifolia
X1Sstrictifolia.Z.adj
30
20
10
Source1
Source2
19Stenuifolia
X19Stenuifolia.Z.adj
25
20
15
Source1
Source2

Fig. S21

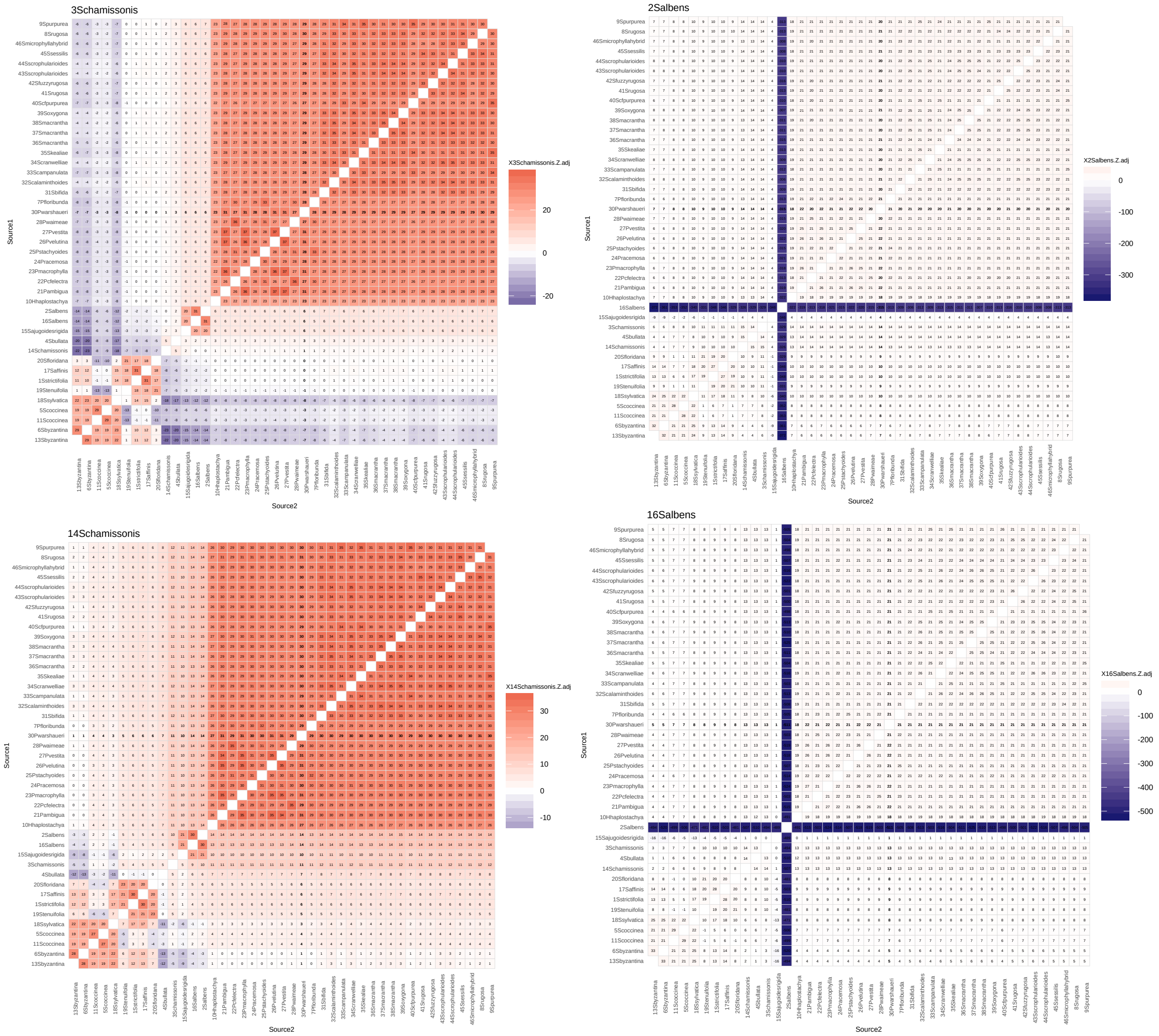
3Schamissonis
X3Schamissonis.Z.adj
20
0
-20
2Salbens
X2Salbens.Z.adj
0
-100
-200
-300
14Schamissonis
X14Schamissonis.Z.adj
30
20
10
0
-10
16Salbens
X16Salbens.Z.adj
0
-100
-200
-300
-400
-500
Source1
Source2

Fig. S21

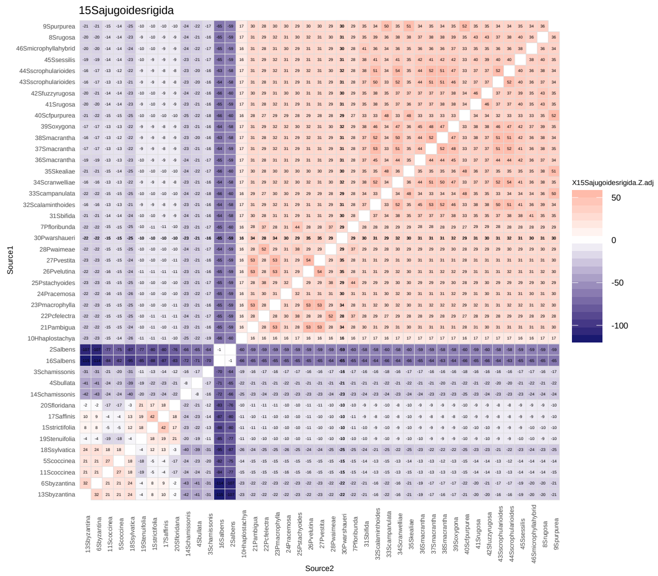
15Sajugoidesrigida
X15Sajugoidesrigida.Z.adj
Source1
Source2

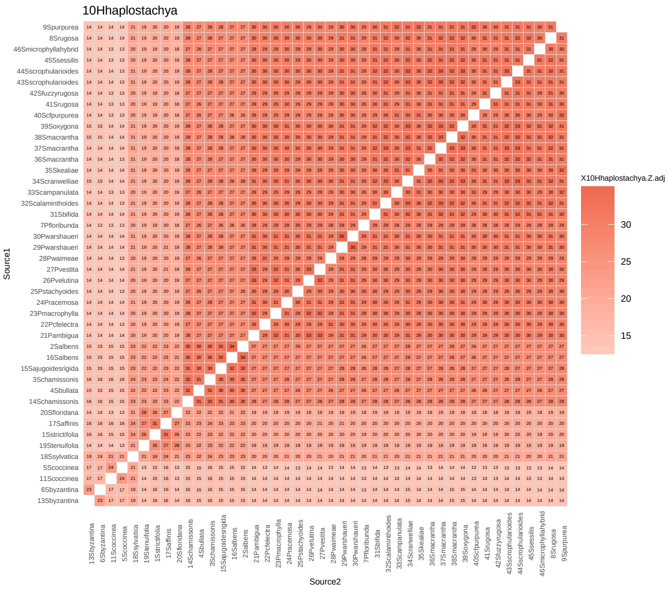
10Hhaplostachya
X10Hhaplostachya.Z.adj
Source1
Source2

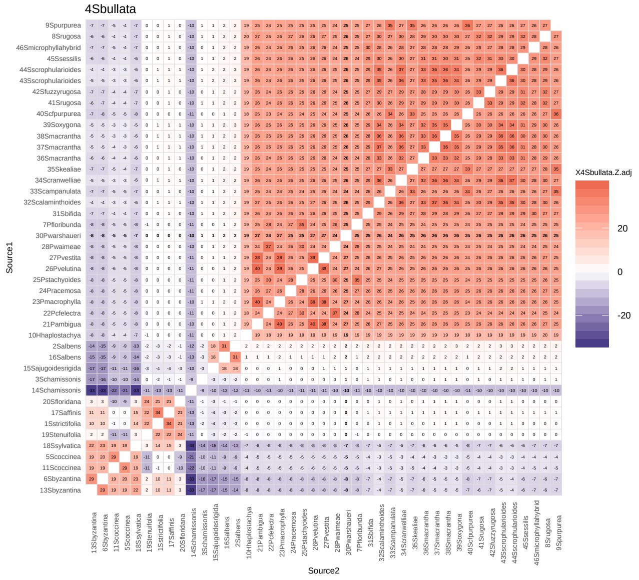
4Sbullata
X4Sbullata.Z.adj
Source1
Source2

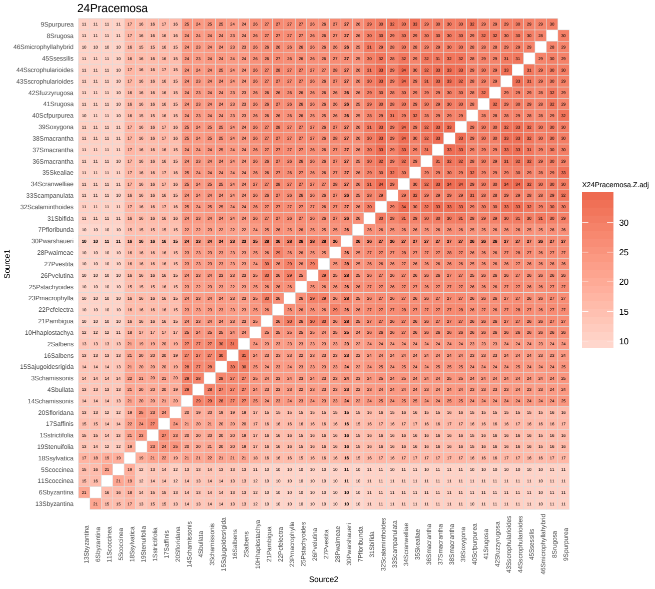
24Pracemosa
X24Pracemosa.Z.adj
Source1
Source2

Fig. S21

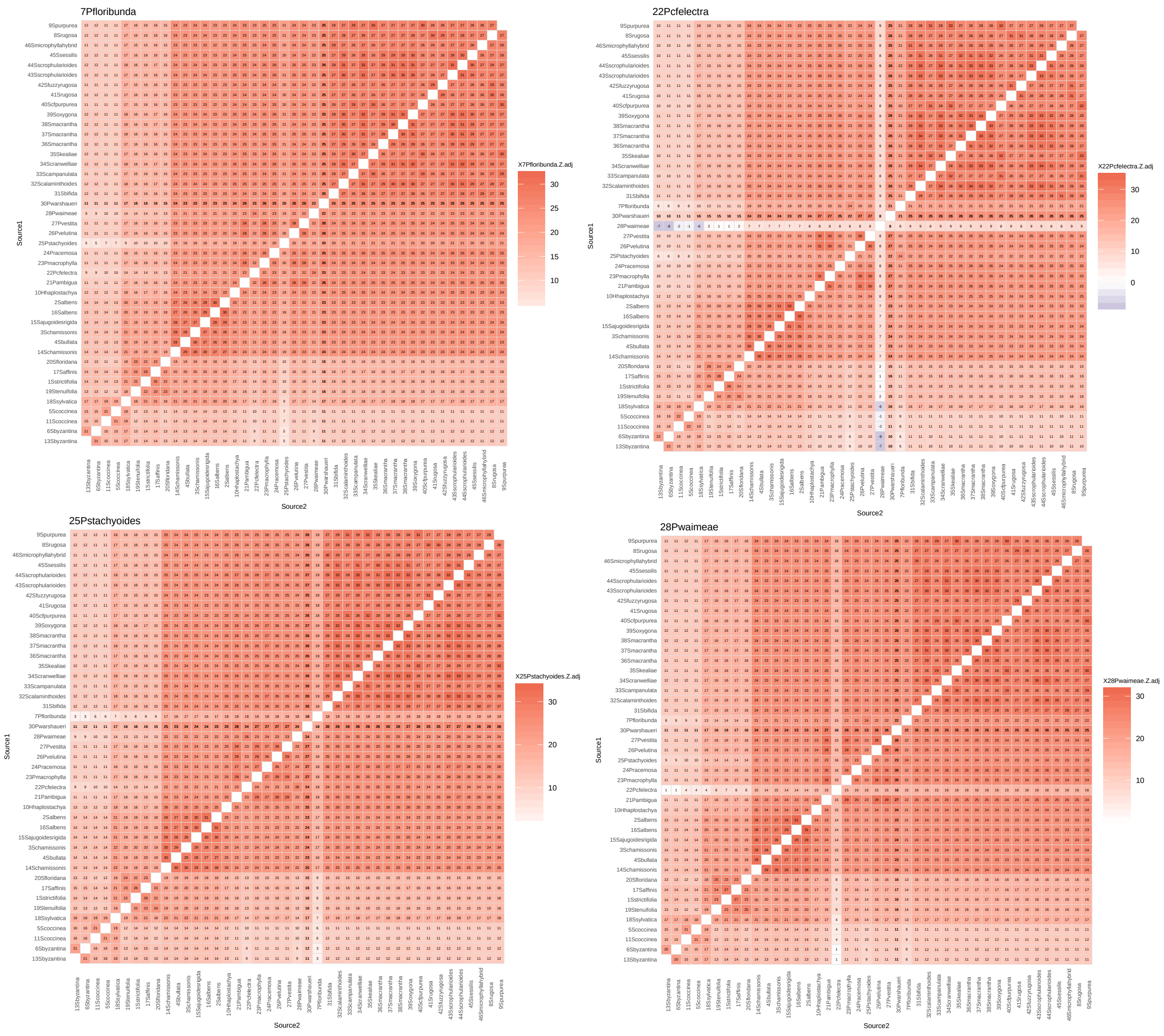

7Pfloribunda
X7Pfloribunda.Z.adj
22Pcfelectra
X22Pcfelectra.Z.adj
25Pstachyoides
X25Pstachyoides.Z.adj
28Pwaimeae
X28Pwaimeae.Z.adj
Source1
Source2

Fig. S21

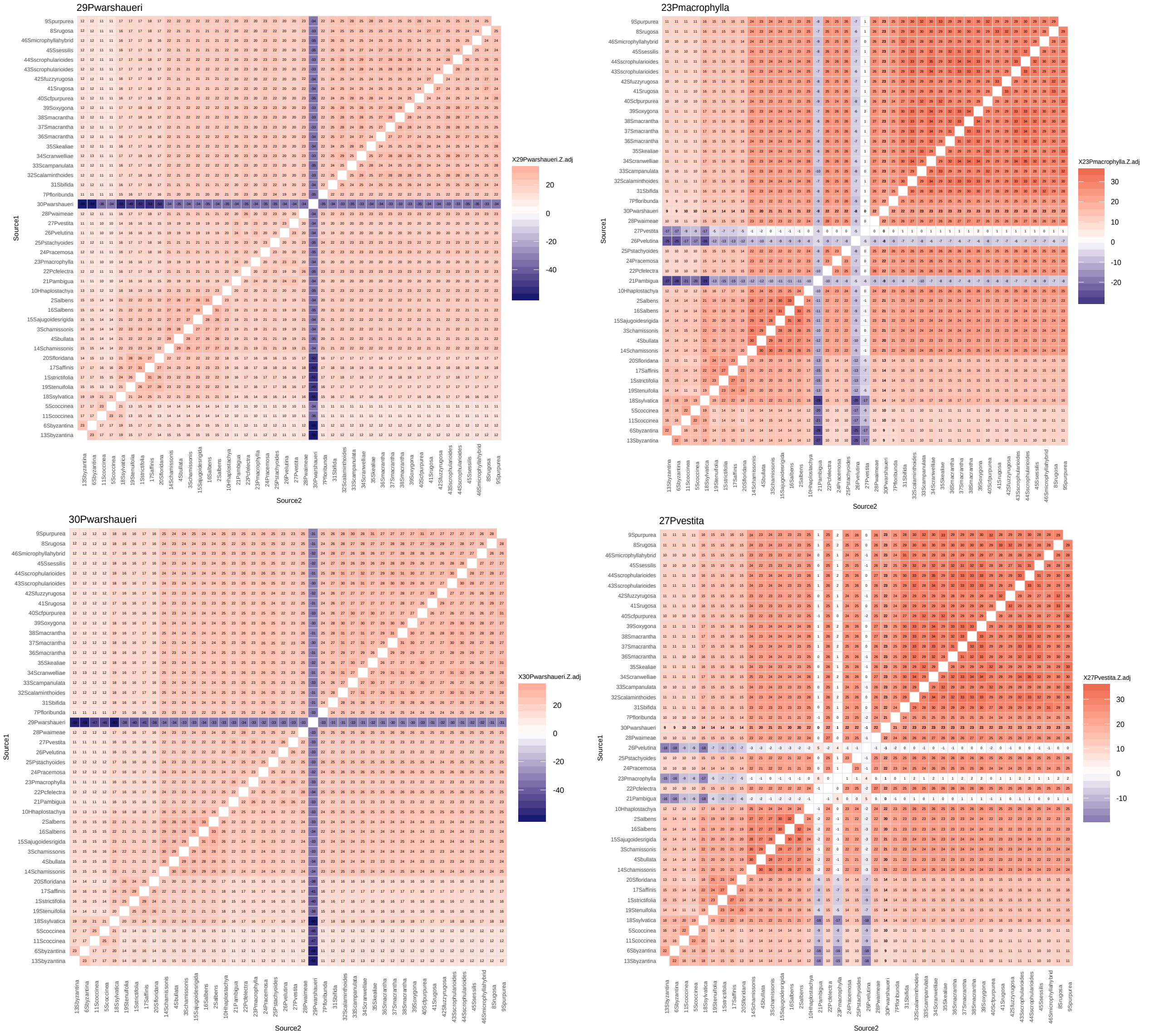

29Pwarshaueri
Source1
Source2
X29Pwarshaueri.Z.adj
23Pmacrophylla
X23Pmacrophylla.Z.adj
30Pwarshaueri
X30Pwarshaueri.Z.adj
27Pvestita
X27Pvestita.Z.adj

Fig. S21

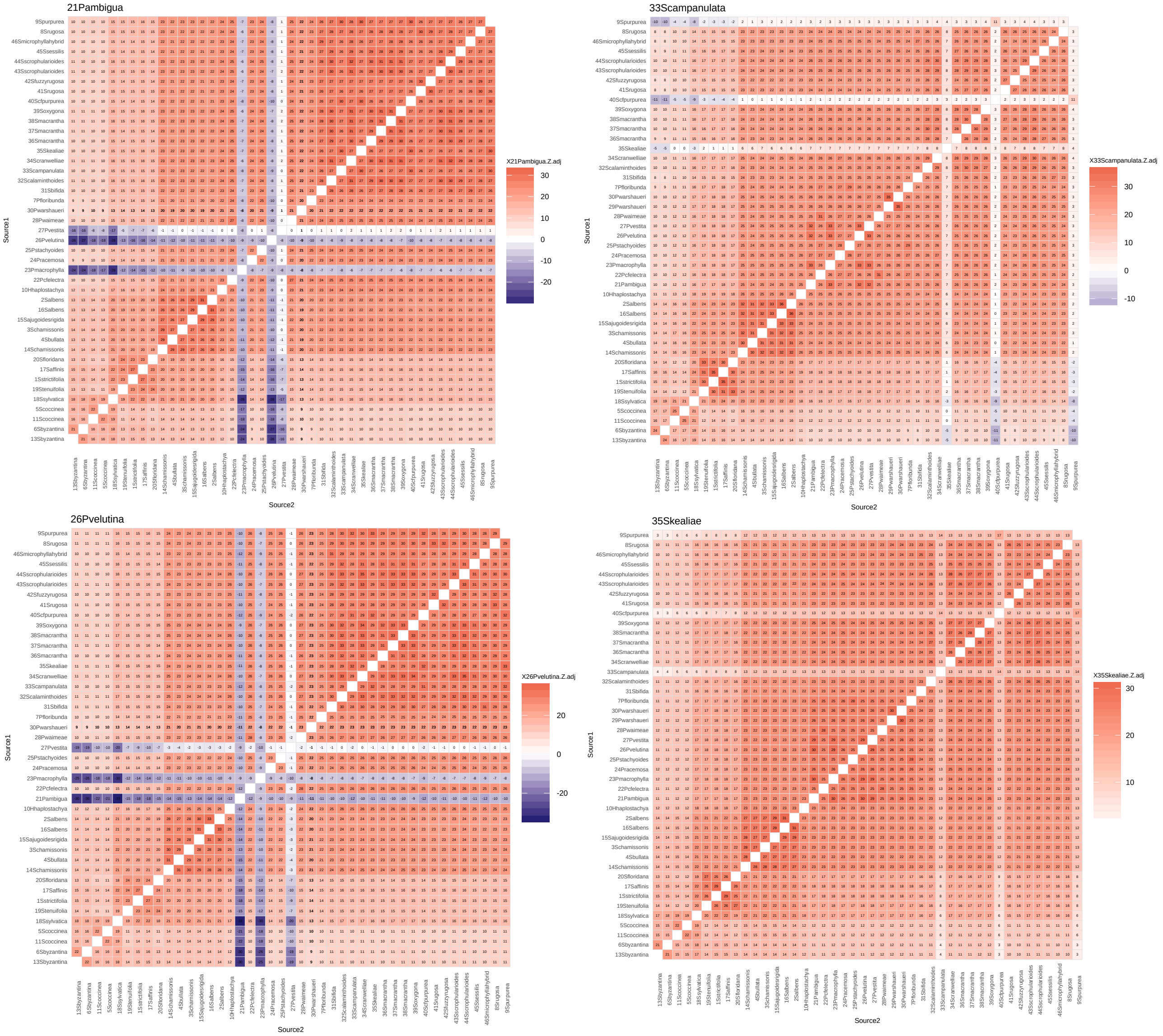

21Pambigua
X21Pambigua.Z.adj
33Scampanulata
X33Scampanulata.Z.adj
26Pvelutina
X26Pvelutina.Z.adj
35Skealiae
X35Skealiae.Z.adj
Source1
Source2

Fig. S21

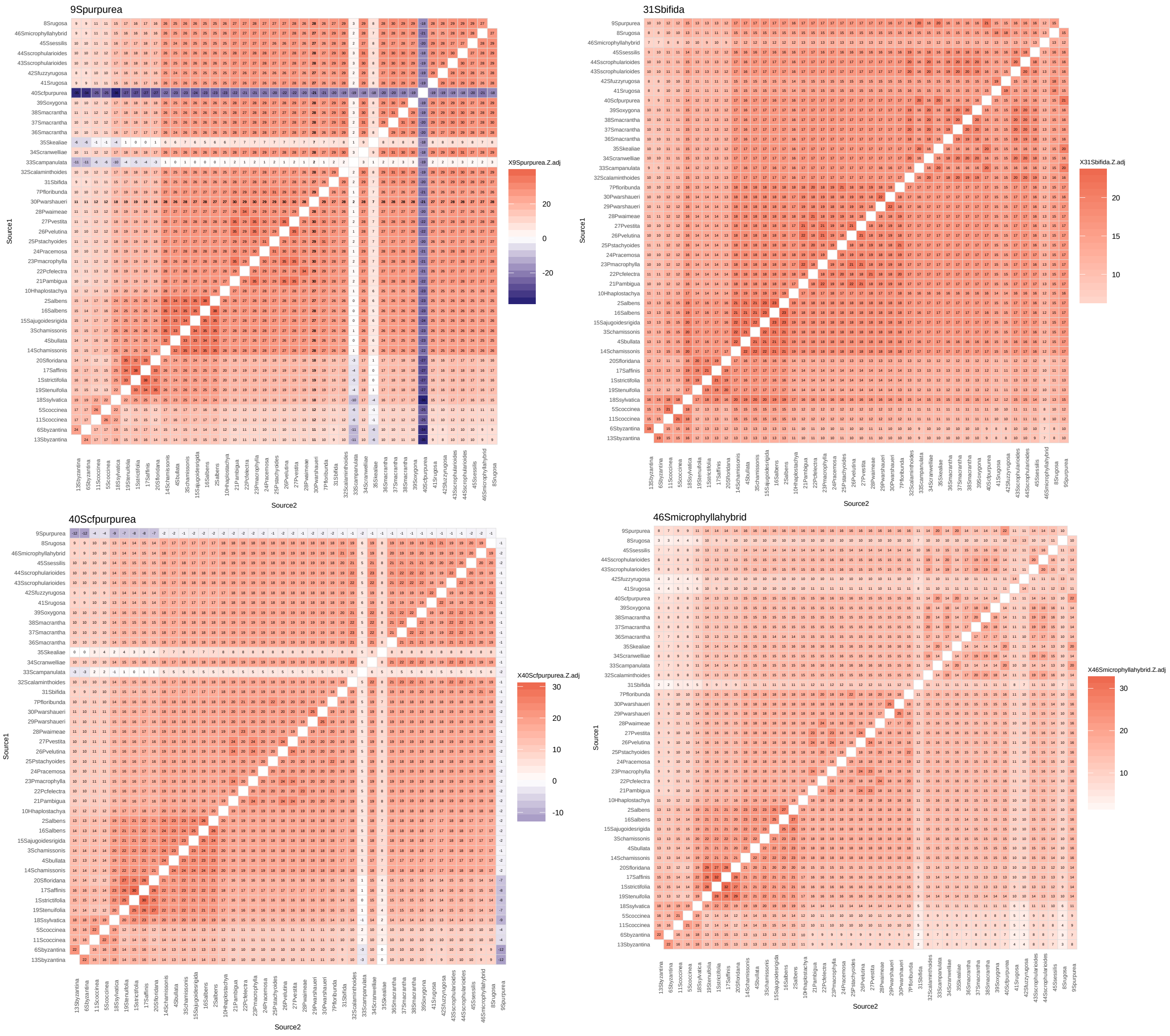

9Spurpurea
X9Spurpurea.Z.adj
31Sbifida
X31Sbifida.Z.adj
40Scfpurpurea
X40Scfpurpurea.Z.adj
46Smicrophyllahybrid
X46Smicrophyllahybrid.Z.adj
Source1
Source2

Fig. S21

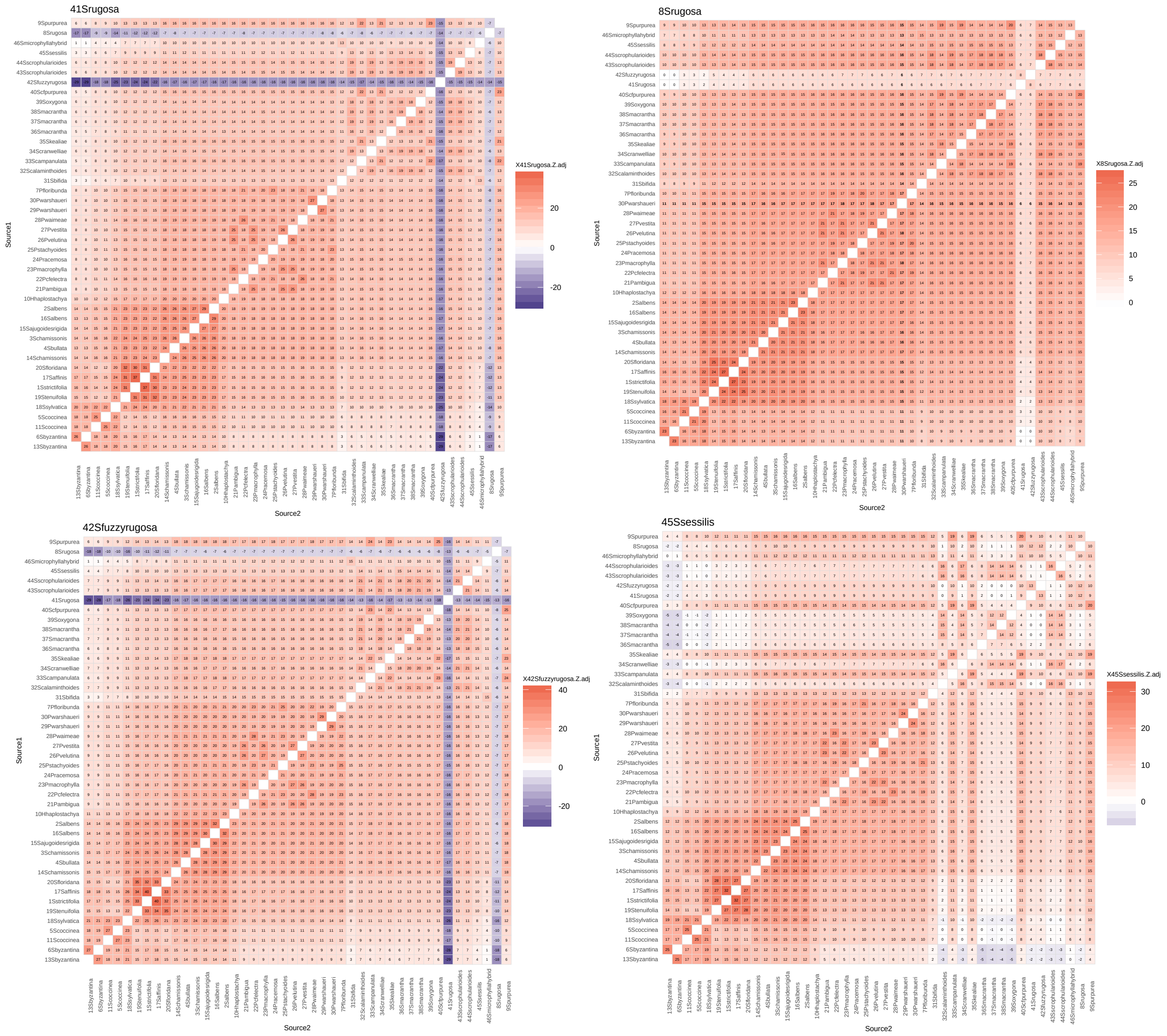

41Srugosa
8Srugosa
42Sfuzzyrugosa
45Ssessilis
Source1
Source2
X41Srugosa.Z.adj
X8Srugosa.Z.adj
X42Sfuzzyrugosa.Z.adj
X45Ssessilis.Z.adj

Fig. S21

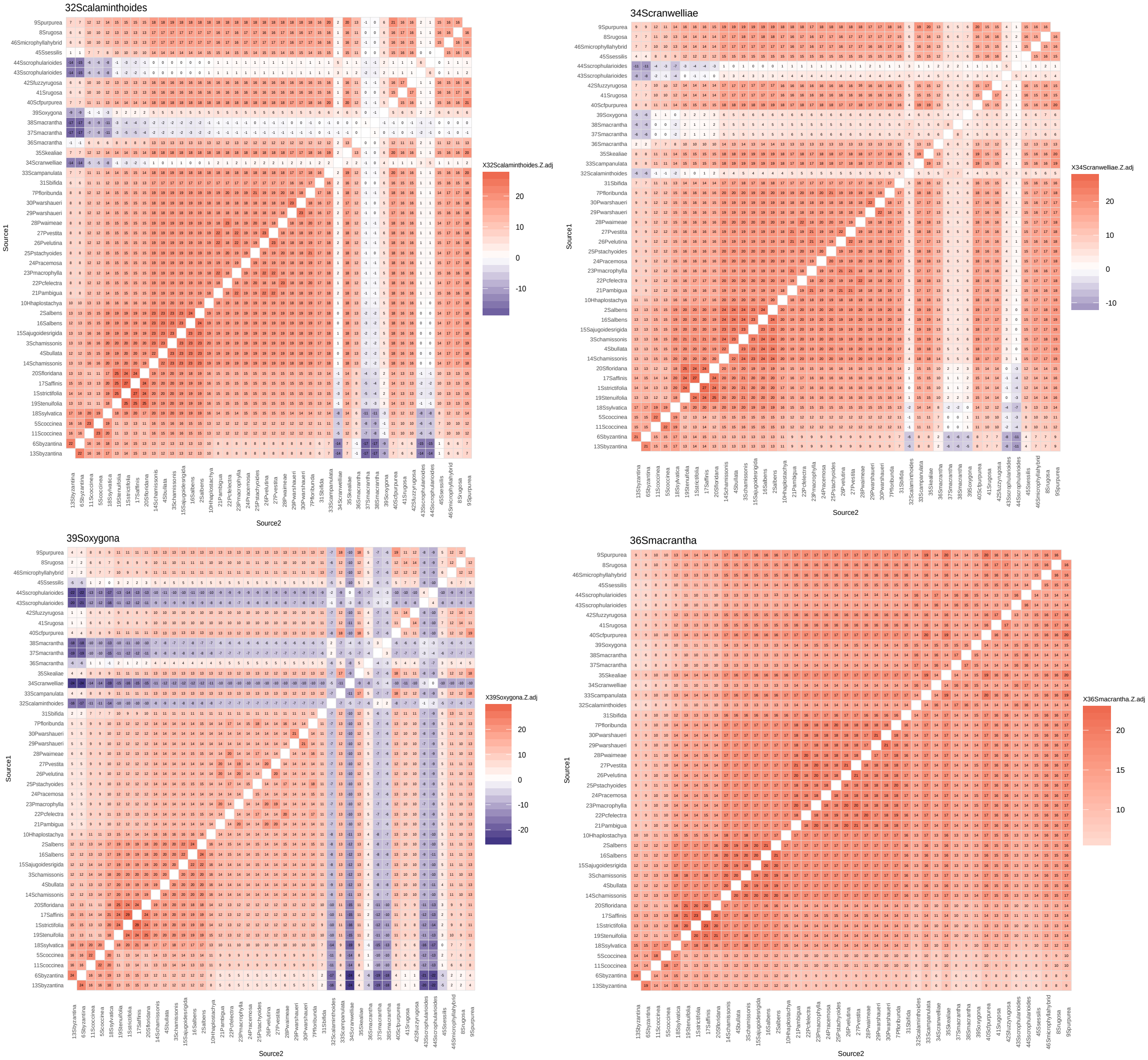
32Scalaminthoides
X32Scalaminthoides.Z.adj
34Scranwelliae
X34Scranwelliae.Z.adj
39Soxygona
X39Soxygona.Z.adj
36Smacrantha
X36Smacrantha.Z.adj
Source1
Source2

Fig. S21

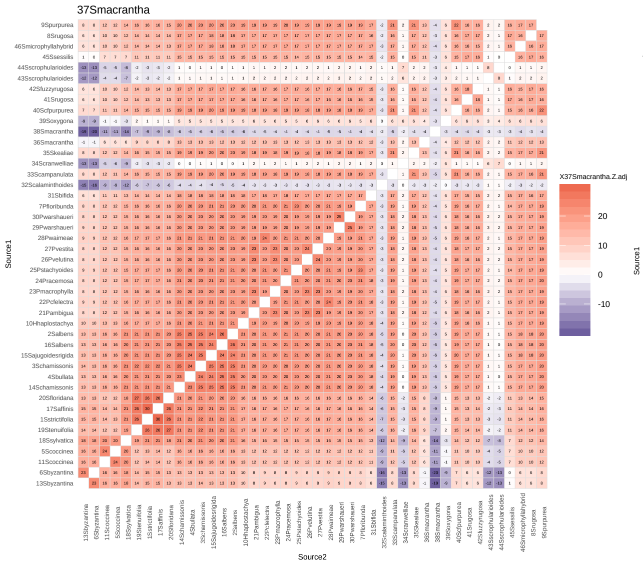

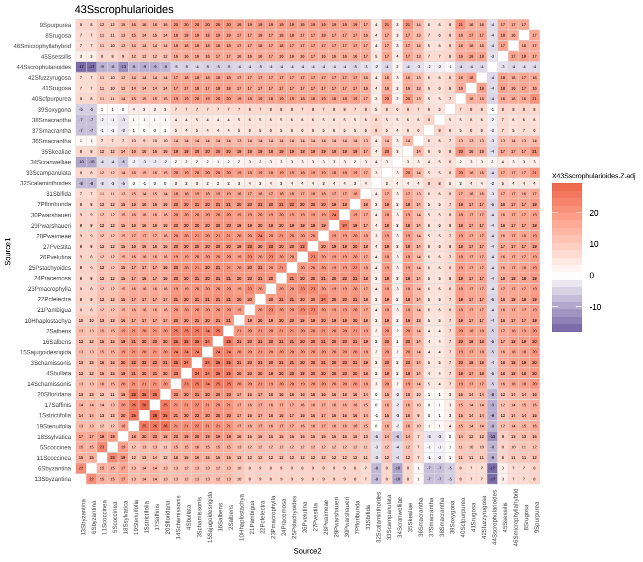

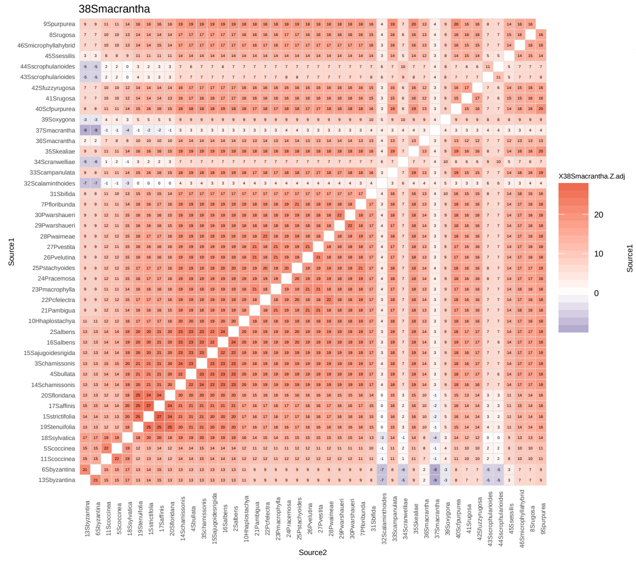

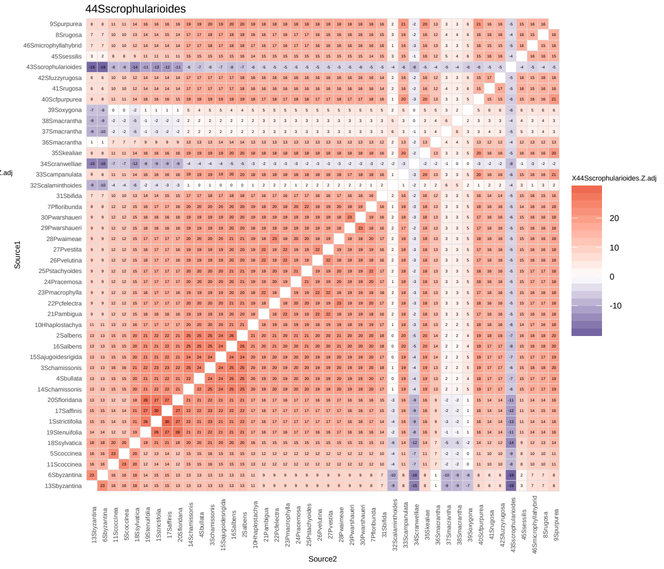

Supplementary Fig. 21: *f3*-statistics plots with all possible 3-way combinations of samples in this study and each sample configured as a target.

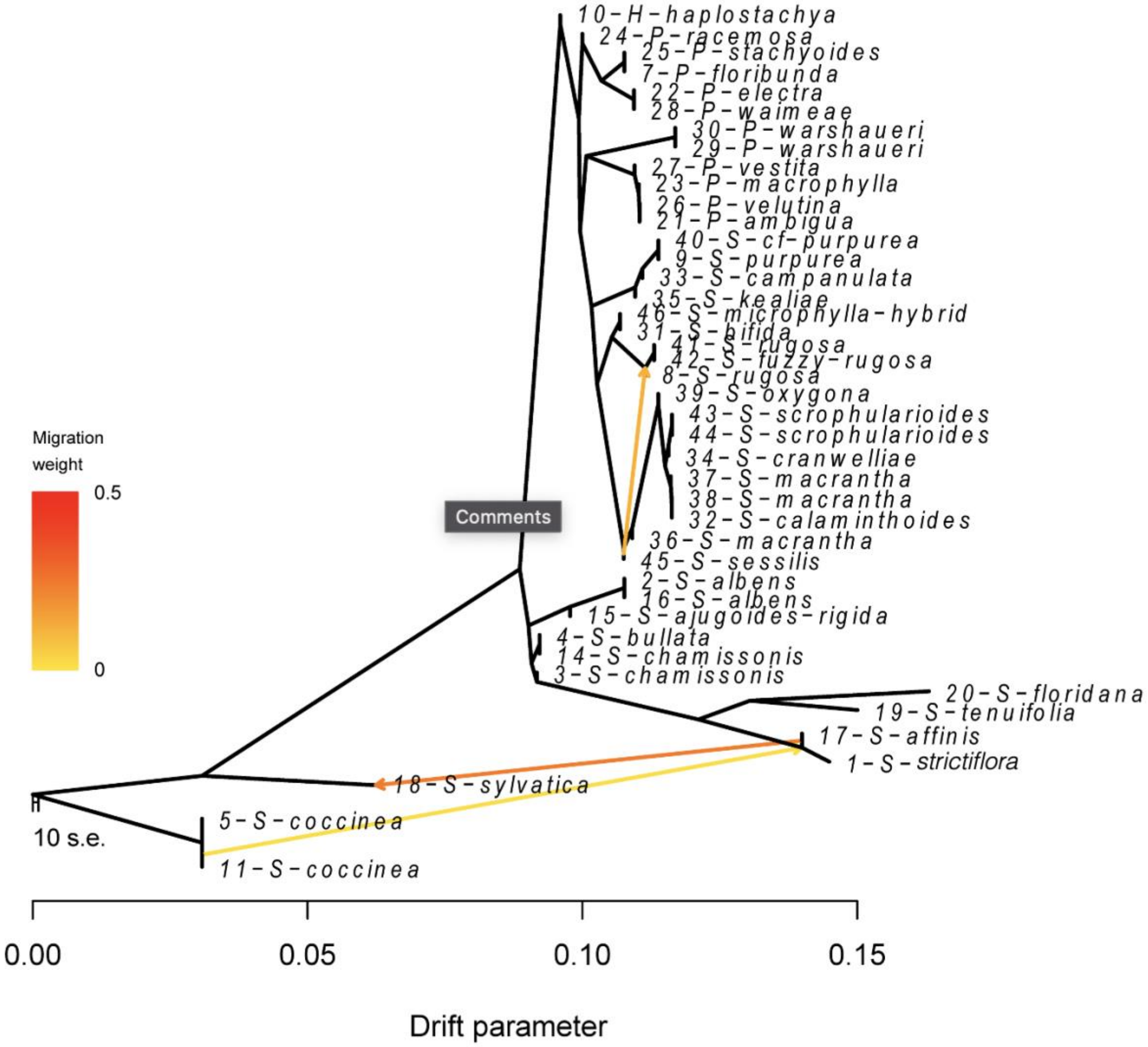

Supplementary Fig. 22: TreeMix of the dataset including all samples, except *Stachys byzantina* (dataset DS4c), showing three migration edges.

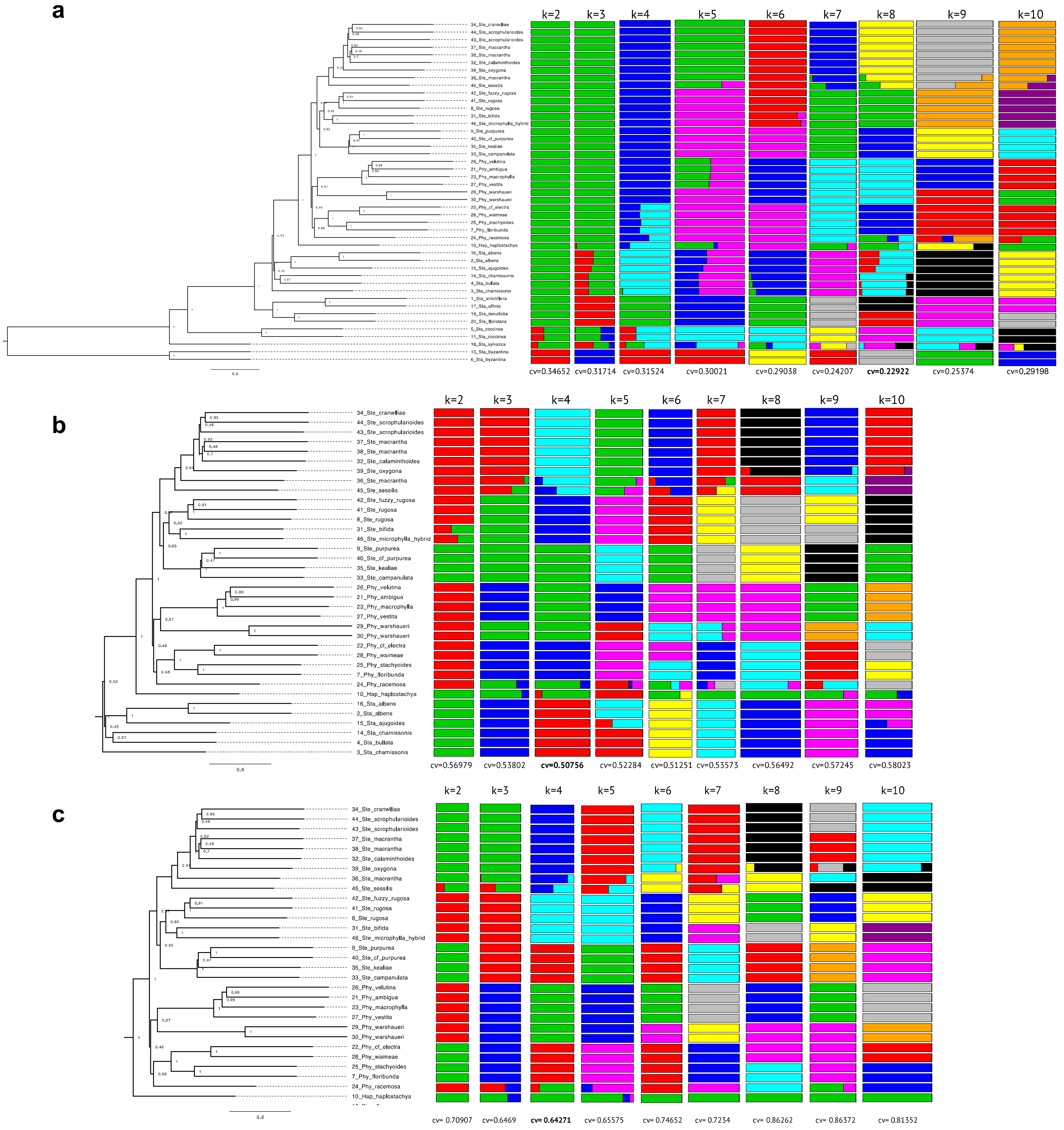

Supplementary Fig. 23: ADMIXTURE plots for $K = 2$ through $K = 10$ based on three datasets: **a** All samples (dataset DS4). **b** Hawaiian mints and WNA *Stachys* (dataset DS4a). **c** Hawaiian mints only (dataset DS4b). The results are displayed alongside the BUSCO tree and the cv values are reported under each K value, with the best cv in bold.

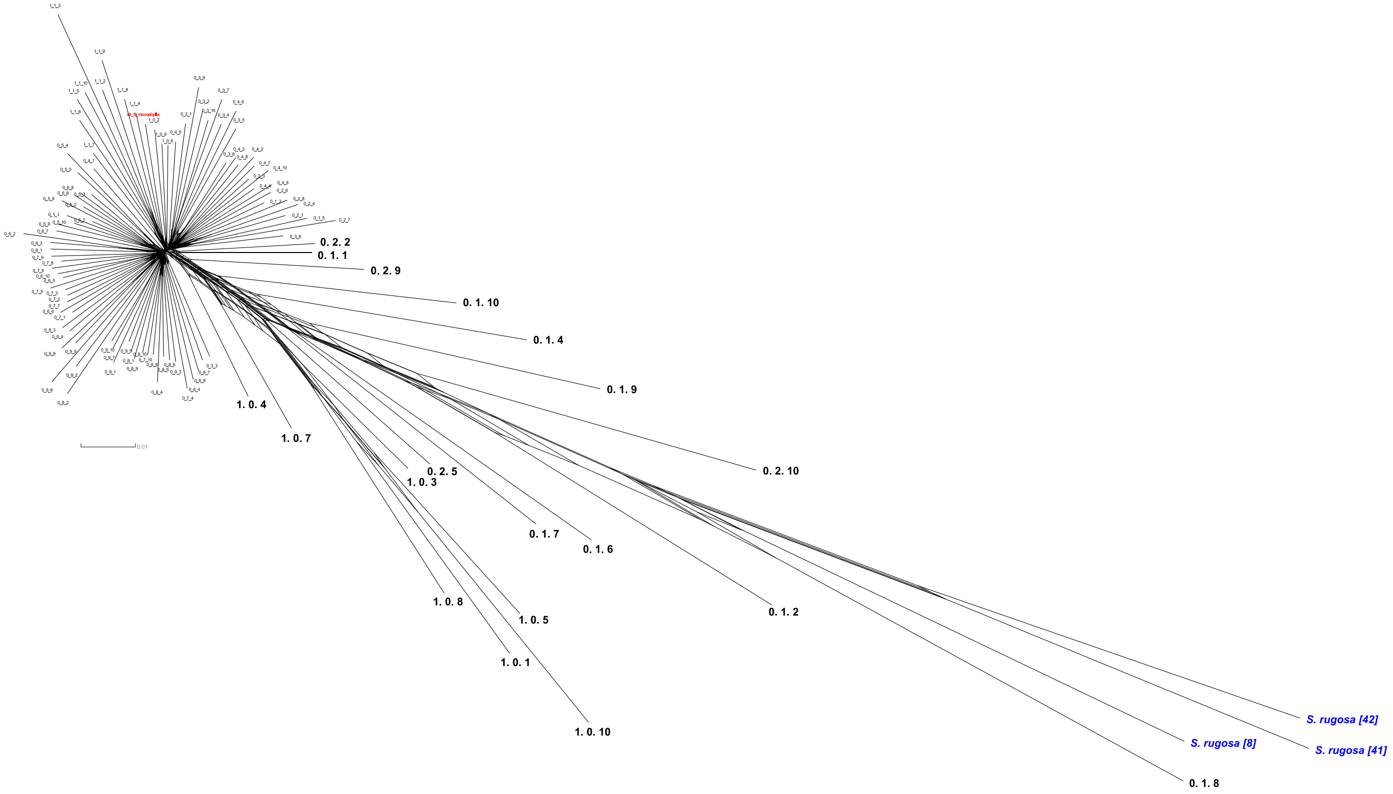

Supplementary Fig 24: NeighborNet based on SNP data from collected samples of *Stenogyne microphylla* and *S. rugosa* and their putative hybrids along the Kaaliali trail on Mauna Kea (dataset HM1). Included are three presumed unadmixed representatives of *S. rugosa* (in blue), and one ostensibly unadmixed *S. microphylla* (in red).

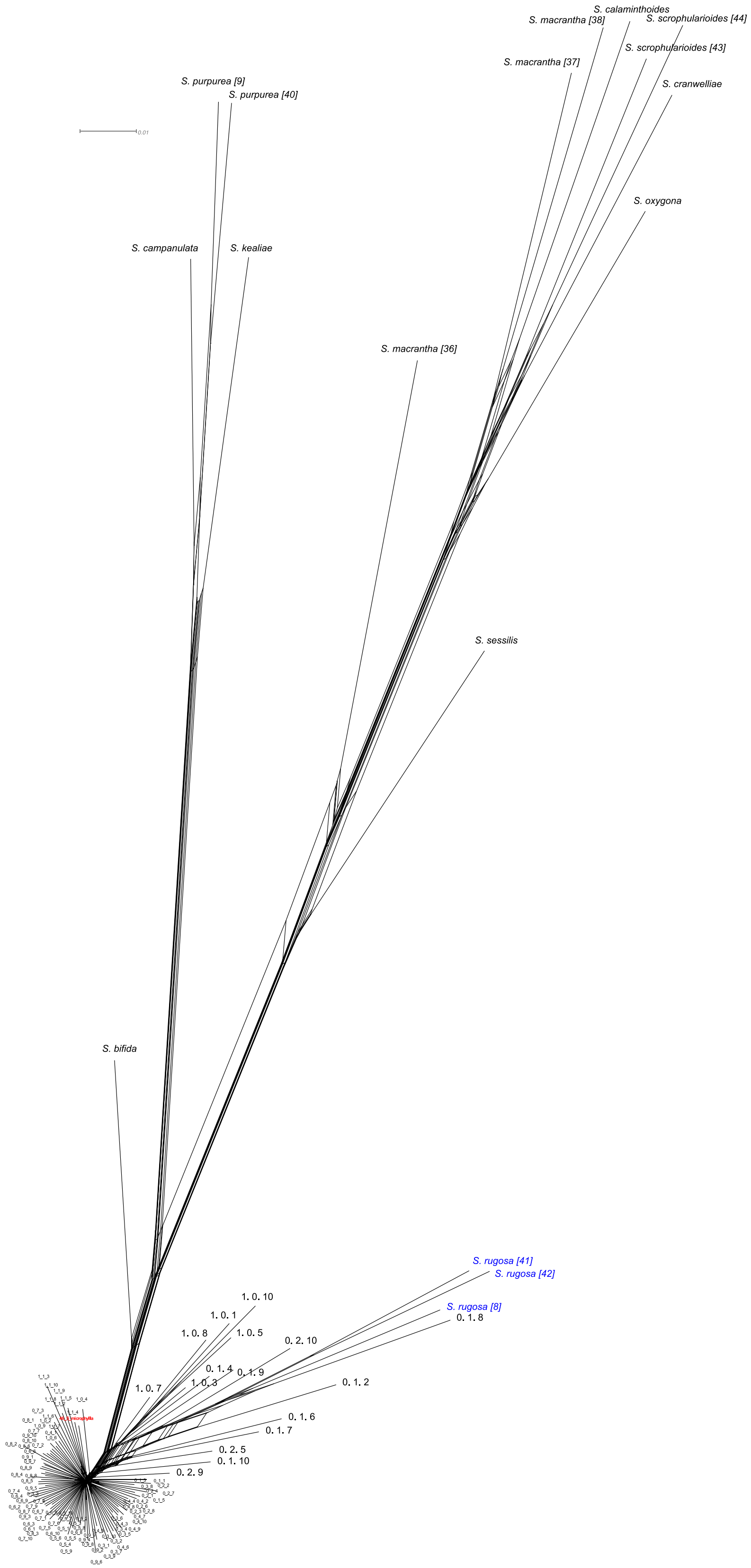

Supplementary Fig. 25: NeighborNet based on SNP data from all sequenced *Stenogyne* samples (dataset HM2).

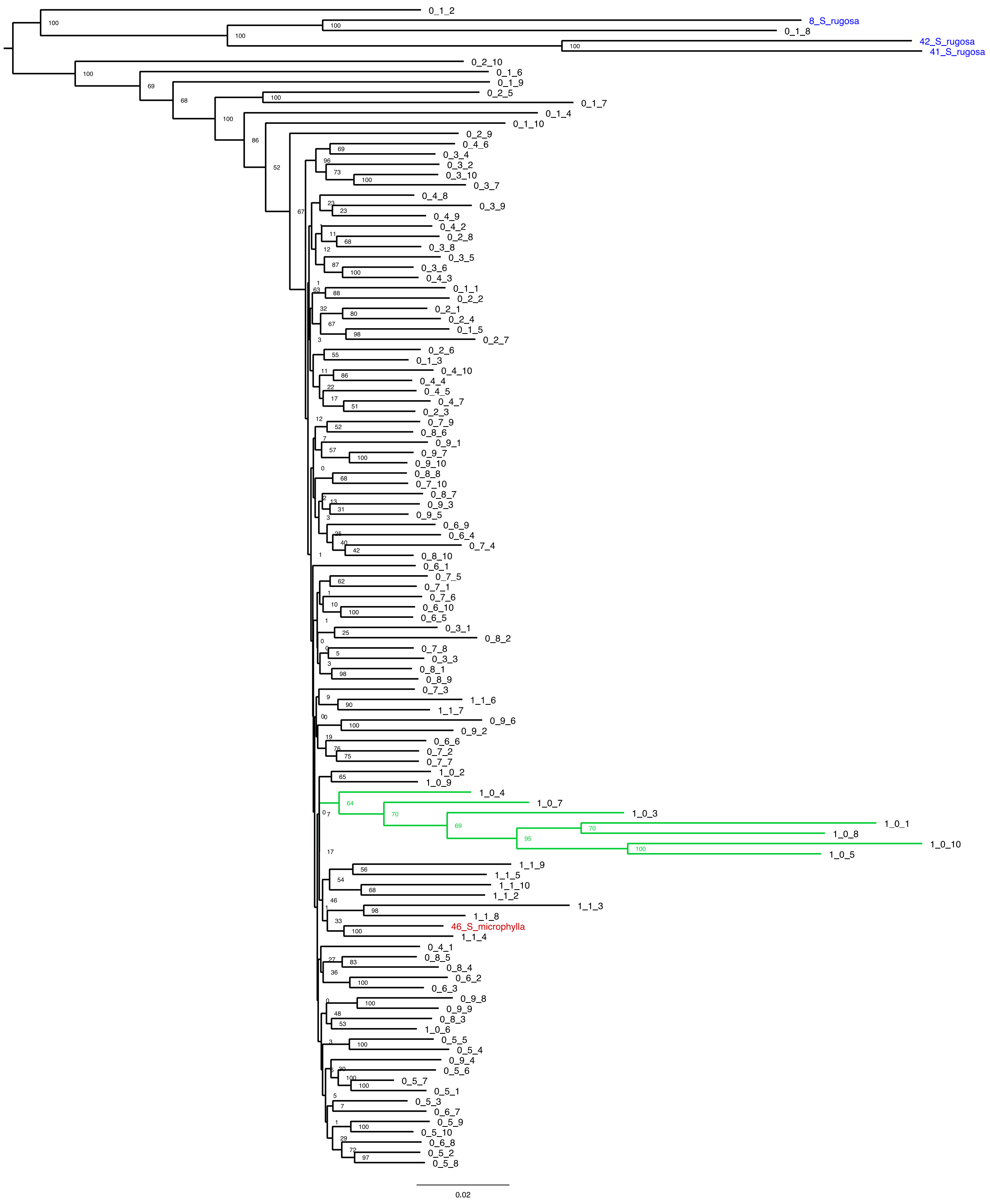

Supplementary Fig. 26: Phylogenetic tree based on SNPs of *Stenogyne microphylla* and *S. rugosa* samples and their putative hybrids along the Kaaliali trail on Mauna Kea (dataset HM1). Presumed unadmixed *S. rugosa* individuals are in blue color and the unadmixed *S. microphylla* is in red. Branches of the lineage with putative ghost admixture are shown in green.

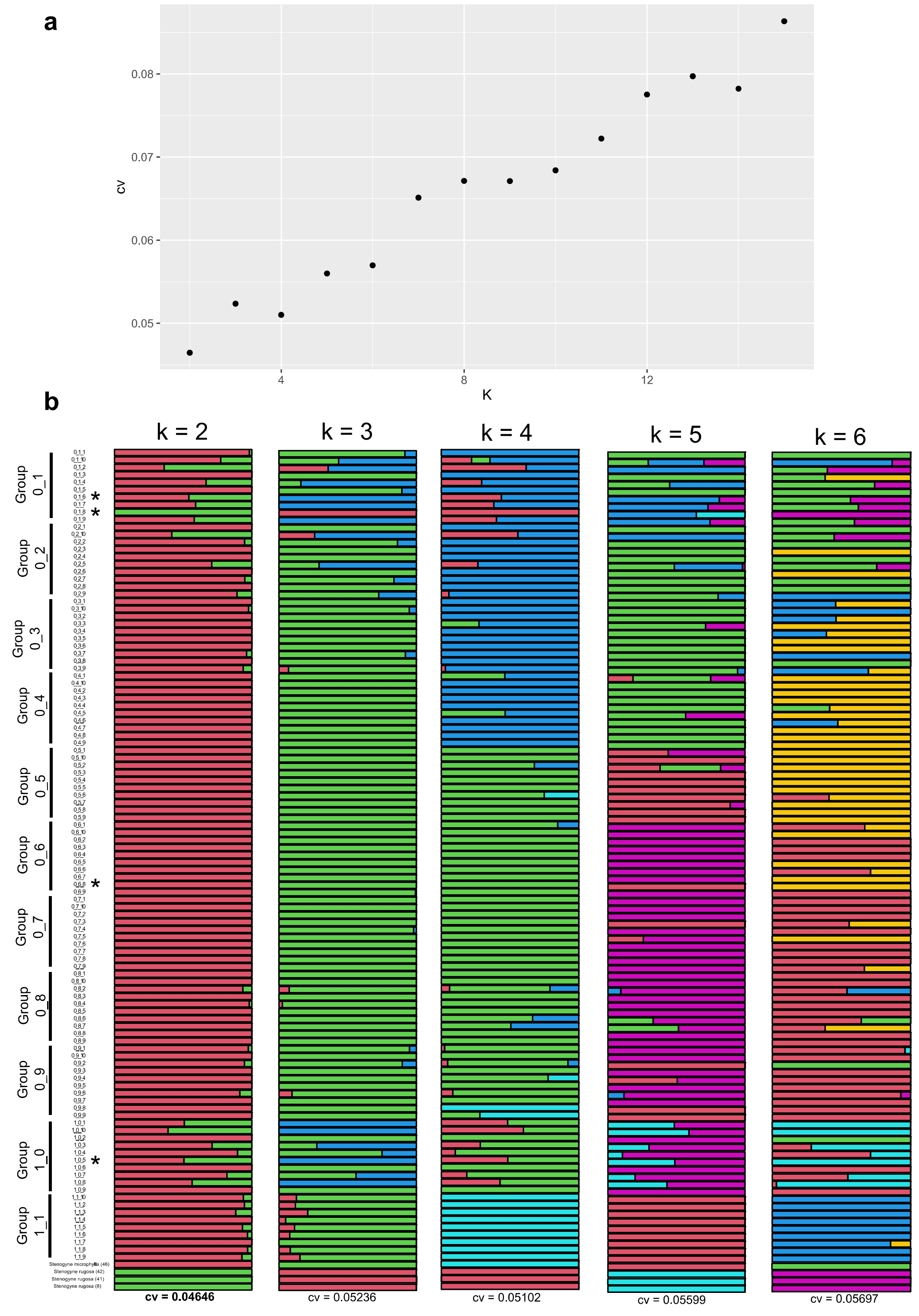

Supplementary Fig. 27: ADMIXTURE analyses based on SNP data from collected samples of *Stenogyne microphylla* and *S. rugosa* and their putative hybrids along the Kaaliali trail on Mauna (dataset HM1). **a** Scatterplot of cross validation (cv) values for K =1 to K = 15. **b** ADMIXTURE plots showing K =2 to K = 6, with cv values below the plot and the best value in bold. *Individuals selected for f_3-statistics analyses (Supplementary Fig. 28).

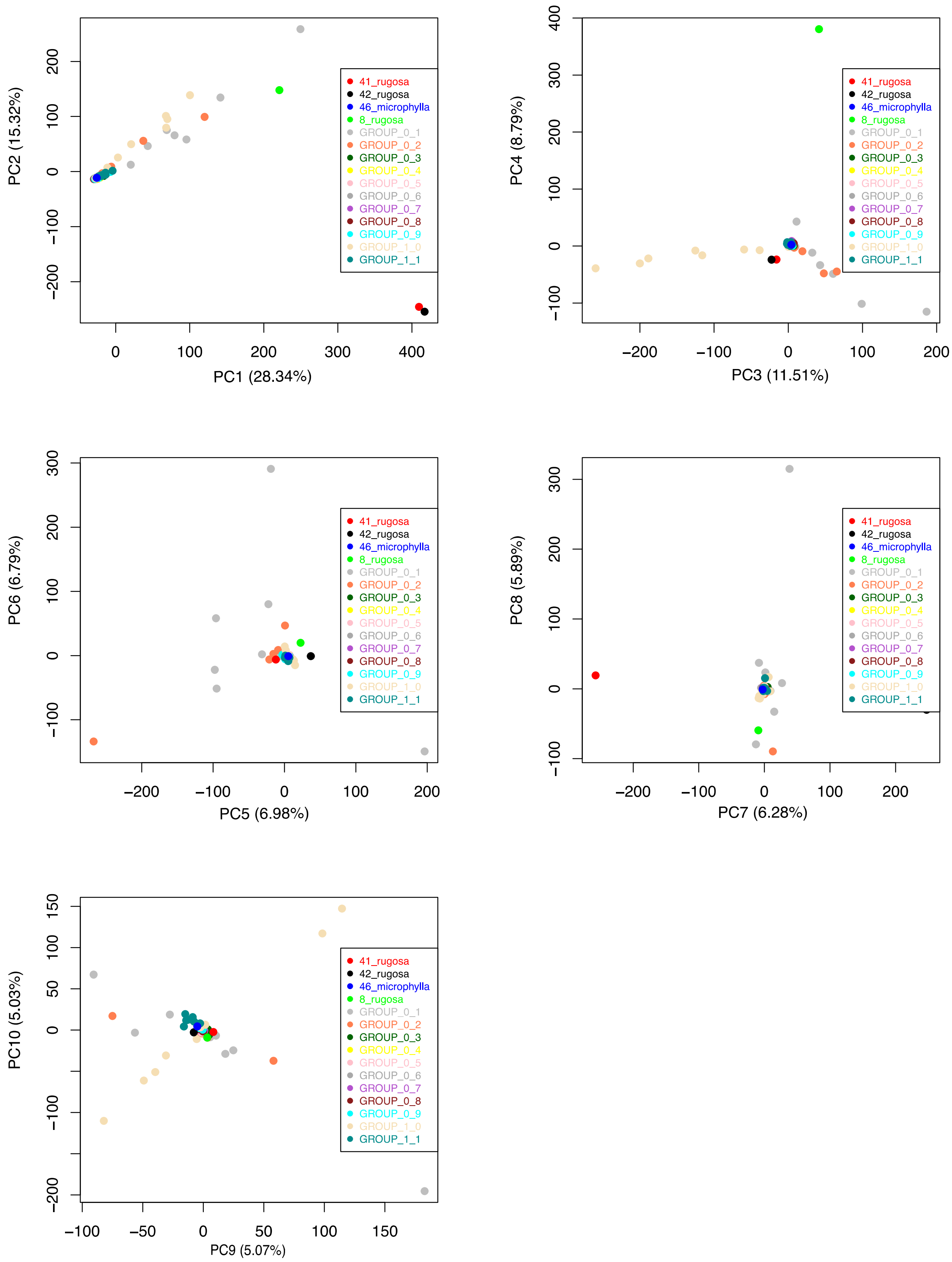

Supplementary Fig. 28: PCA plots for PC1 through PC10 for all collected samples of *Stenogyne microphylla* and *S. rugosa* and their putative hybrids (color coded by group; dataset HM1). Percent variation for each PC is shown in parentheses along axes.

Fig. S29

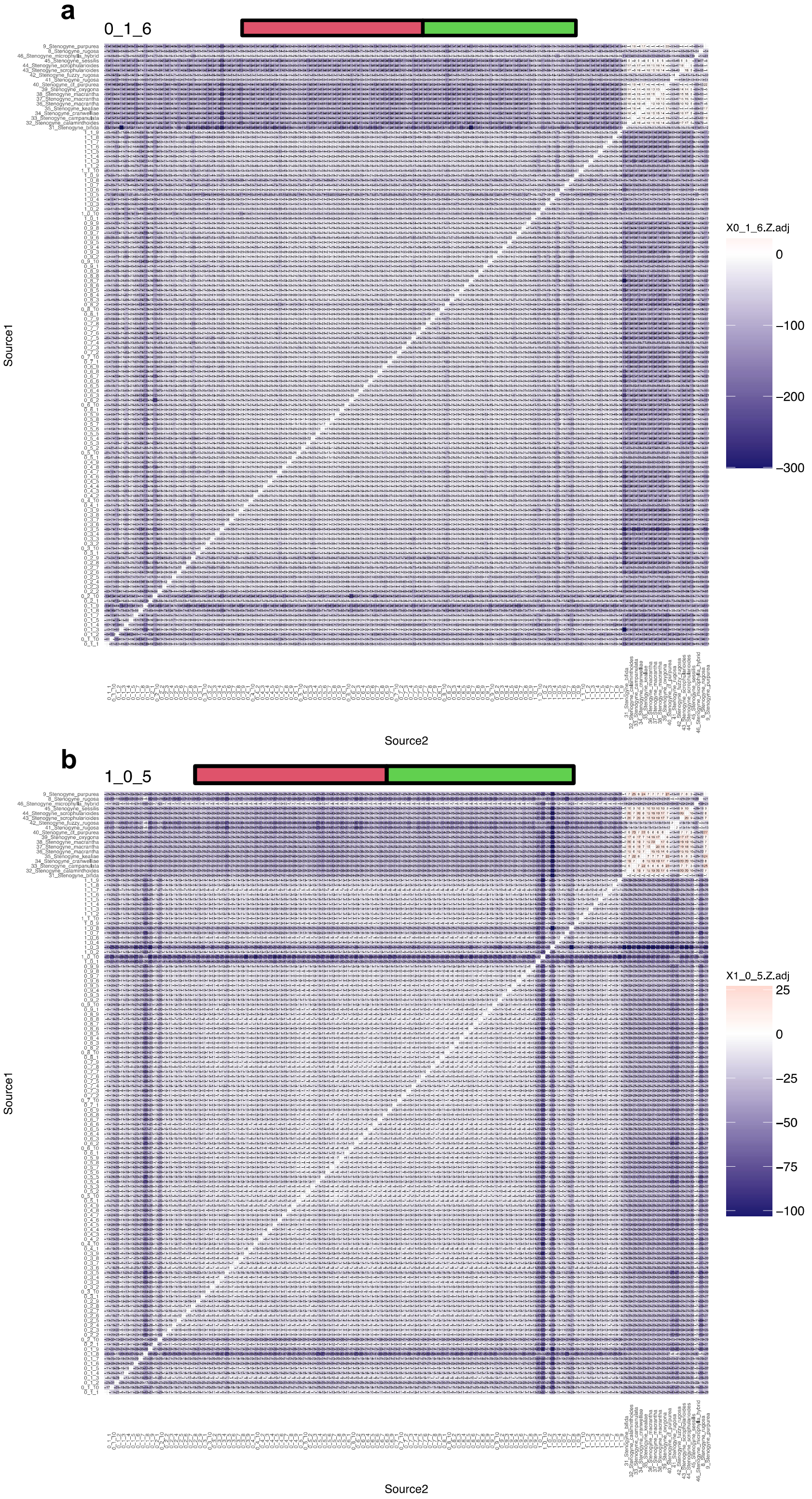
a
0_1_6
Source1
Source2
X0_1_6.Z.adj
0
−100
−200
−300
b
1_0_5
Source1
Source2
X1_0_5.Z.adj
25
0
−25
−50
−75
−100

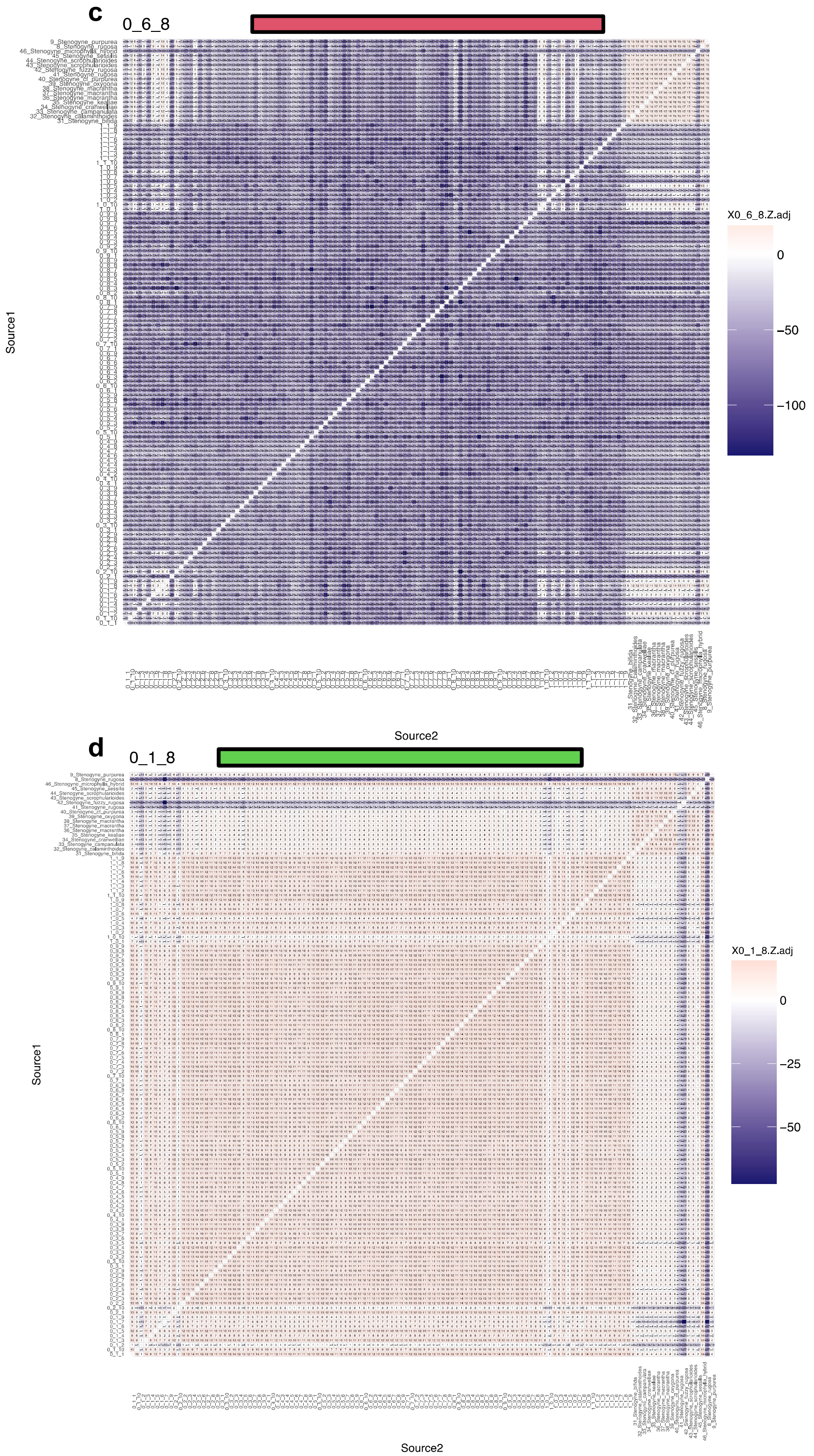

Supplementary Fig. 29: f_3-statistics heatmaps based on SNP data (dataset HM4) from selected samples of *Stenogyne microphylla* and *S. rugosa* and their putative hybrids that appeared admixed and unadmixed in ADMIXTURE. ADMIXTURE bars for the samples based on $K = 2$ are shown for reference above the plots. **a** Sample 0.1.6. **b** Sample 1.0.5. **c** Sample 0.6.8. **d** Sample 0.1.8.

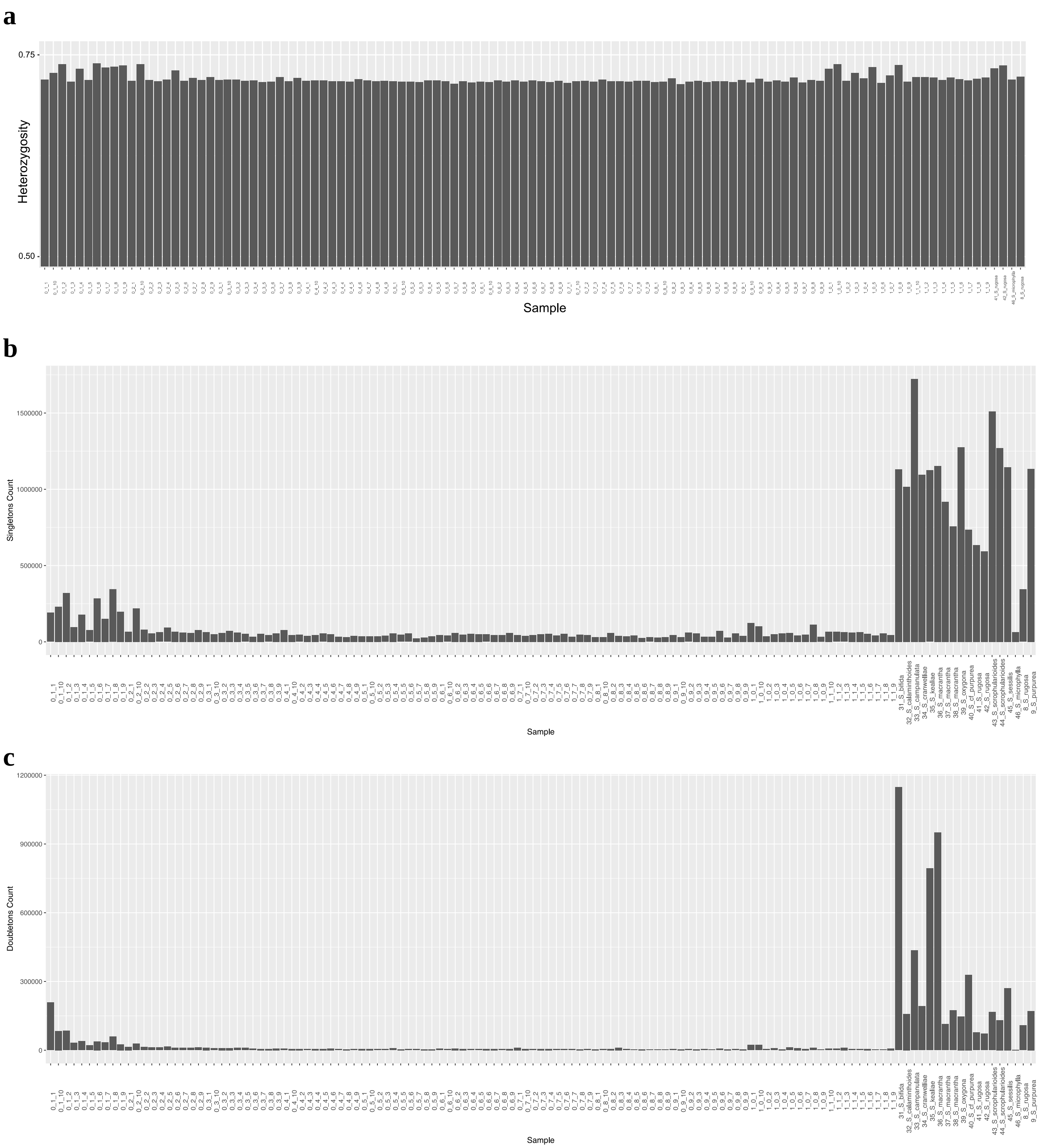

Supplementary Fig. 30: a Heterozygosity of *Stenogyne* species and samples of *S. microphylla* and *S. rugosa* and their putative hybrids on Mauna Kea. **b** Singletons in the hybrid swarm. **c** Doubletons in the hybrid swarm.

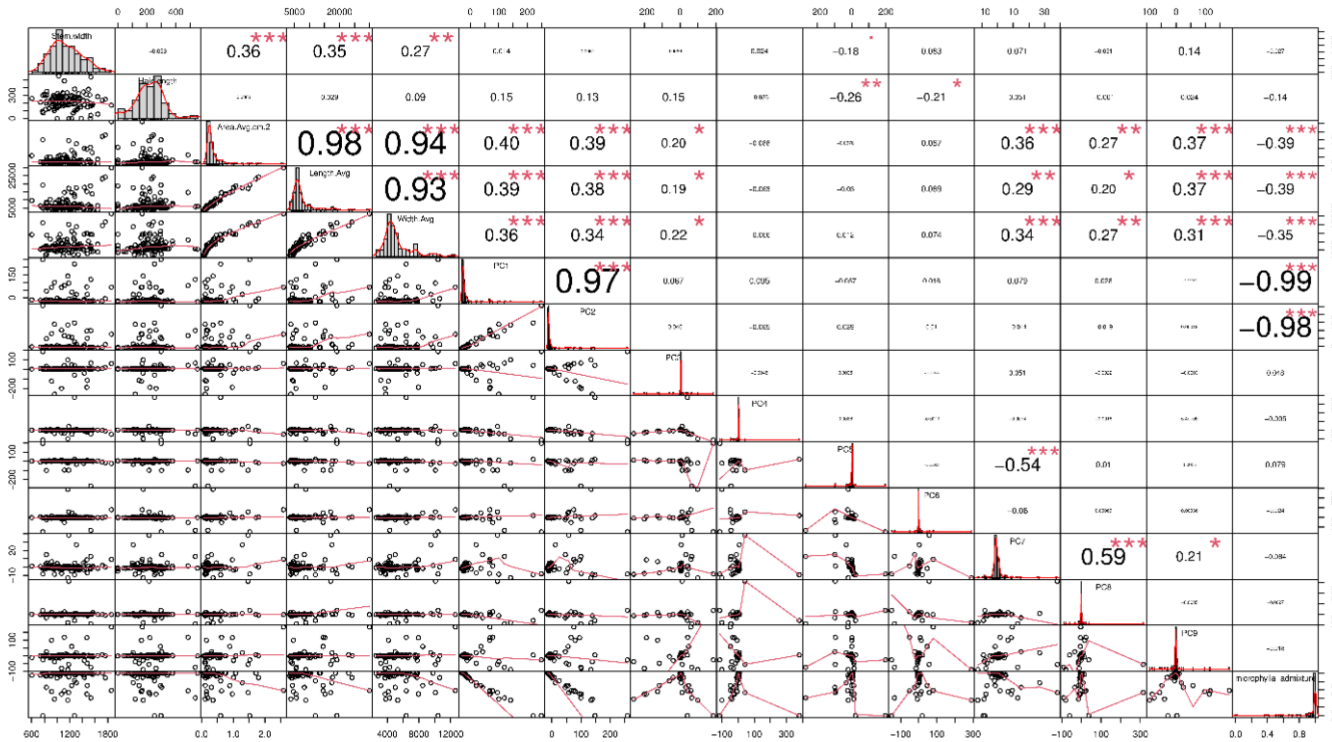

Supplementary Fig. 31: Correlation plot of morphological variables and SNP based PCs for samples of *Stenogyne microphylla* and *S. rugosa* and their putative hybrids on Mauna Kea.

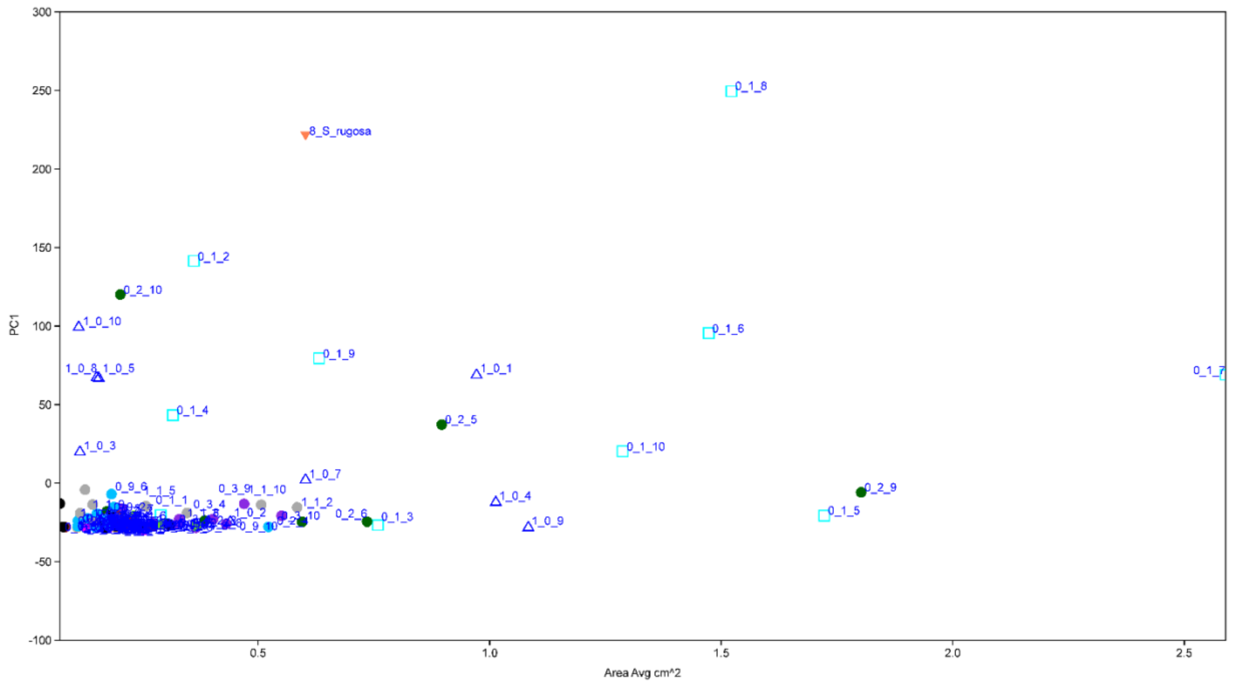

Supplementary Fig. 32: Scatterplot of PC1 and average leaf area for samples of *Stenogyne microphylla* and *S. rugosa* and their putative hybrids on Mauna Kea.
