# Supplementary material for: Allopolyploid origin and diversification of the Hawaiian endemic mints
Source: Nat Commun. 2024 Apr 10;15:3109. doi: 10.1038/s41467-024-47247-y (PMC11006916; doi:10.1038/s41467-024-47247-y)
Supplement: Supplementary file 3 — Description of Additional Supplementary Files [file 41467_2024_47247_MOESM3_ESM.pdf]

Description of Additional Supplementary Files

File Name: Supplementary Data 1
Description: Repeat characterization and statistics of the Stenogyne calaminthoides reference, as reported by the EDTA annotation pipeline.

File Name: Supplementary Data 2
Description: Assembly statistics for Stenogyne calaminthoides reference.

File Name: Supplementary Data 3
Description: BUSCO statistics and N50 for the reference assembly and corresponding gene models.

File Name: Supplementary Data 4
Description: Information for the samples used in this dataset, including sources and geographic distribution. UB = University at Buffalo, UNA = University of Alabama.

File Name: Supplementary Data 5
Description: Reference mapping statistics for each sample in this study.

File Name: Supplementary Data 6
Description: Single nucleotide polymorphisms (SNP) datasets used in this study, their corresponding filtering and downstream analyses employed.

File Name: Supplementary Data 7
Description: Plastid mapping depth and width statistics.

File Name: Supplementary Data 8
Description: Genome size estimates and various quality assessment statistics for each Illumina based de novo MaSuRCA assembly.

File Name: Supplementary Data 9
Description: Chromosome based mapping depth for each sample mapped to Stenogyne calaminthoides.

File Name: Supplementary Data 10
Description: Syntenic links downloaded from CoGe and reformatted for Circos.

File Name: Supplementary Data 11
Description: Reference-based genome mapping statistics for the putative hybrid swarm samples on Mauna Kea.

File Name: Supplementary Data 12
Description: Morphological characters from samples of the putative hybrid swarm.
