# Supplementary material for: Allopolyploid origin and diversification of the Hawaiian endemic mints
Source: Nat Commun. 2024 Apr 10;15:3109. doi: 10.1038/s41467-024-47247-y (PMC11006916; doi:10.1038/s41467-024-47247-y)
Supplement: Supplementary file 16 — Reporting Summary [file 41467_2024_47247_MOESM16_ESM.pdf]

nature portfolio

Corresponding author(s): Charlotte Lindqvist

Last updated by author(s): March 14, 2024

Reporting Summary

Nature Portfolio wishes to improve the reproducibility of the work that we publish. This form provides structure for consistency and transparency in reporting. For further information on Nature Portfolio policies, see our Editorial Policies and the Editorial Policy Checklist.

Statistics

For all statistical analyses, confirm that the following items are present in the figure legend, table legend, main text, or Methods section.

n/a	Confirmed	
☐	☒	The exact sample size (*n*) for each experimental group/condition, given as a discrete number and unit of measurement
☒	☐	A statement on whether measurements were taken from distinct samples or whether the same sample was measured repeatedly
☒	☐	The statistical test(s) used AND whether they are one- or two-sided<br>*Only common tests should be described solely by name; describe more complex techniques in the Methods section.*
☒	☐	A description of all covariates tested
☒	☐	A description of any assumptions or corrections, such as tests of normality and adjustment for multiple comparisons
☐	☒	A full description of the statistical parameters including central tendency (e.g. means) or other basic estimates (e.g. regression coefficient) AND variation (e.g. standard deviation) or associated estimates of uncertainty (e.g. confidence intervals)
☒	☐	For null hypothesis testing, the test statistic (e.g. *F*, *t*, *r*) with confidence intervals, effect sizes, degrees of freedom and *P* value noted<br>*Give P values as exact values whenever suitable.*
☒	☐	For Bayesian analysis, information on the choice of priors and Markov chain Monte Carlo settings
☒	☐	For hierarchical and complex designs, identification of the appropriate level for tests and full reporting of outcomes
☐	☒	Estimates of effect sizes (e.g. Cohen's *d*, Pearson's *r*), indicating how they were calculated

Our web collection on statistics for biologists contains articles on many of the points above.

Software and code

Policy information about availability of computer code

Data collection	No software was used for data collection
Data analysis	Jellyfish 2.2.10, KmerGenie v. 1.7048, NanoFilt 2.6.0, NanoStat v. 1.1.2,minimap2 v 2.16-r922, miniasm v. 0.3-r179, Bandage v. 0.8.1, RepeatModeler2, EDTA v 1.8.3, Racon v. 1.3.3, Pilon v. 1.2.3, purge_haplotigs v. 1.1.0, SAMtools 0.1.19, BUSCO, Juicer v. 1.5.7, 3D-DNA v. 5.0.2, Juicebox v. 1.11.08, QUAST v. 5.0.1, Trinity v. 2.6.6, Trans-ABySS v. 2.0.1, EvidentialGene v. 2017.12.21, PASA v. 2.3.3, genemark-es v4.38, BRAKER v. 2.1.2, STAR aligner v. 2.7.2b, AUGUSTUS v3.3.2, GeMoMa v. 1.6.1, EVidenceModeler v. 1.1.1, Circos v 0.69-9, circlize 4.2.1, Trimmomatic v. 0.38, bwa v. 0.7.17, Picard v. 2.7.1, BEDTools v. 2.23.0, NOVOplasty v. 3.0, FigTree v. 1.4.3, MaSuRCA v. 3.2.7, GATK v. 3.8, VCFtools 0.1.13, TranslatorX v 1.1, trimAL v. 1.4.rev22, DECIPHER 2.24.0, RAxML v. 8.0.0, ASTRAL-Pro (ASTER v. 1.15), GRAMPA v. 1.3, SplitsTree4 v. 4.16.2, ape 5.6-2, dendextend 1.17.1, TreeMix 1.13, OptM v. 0.1.3, ADMIXTURE v 1.3, ggplot2 v. 3.3.6, ggrepel v. 3.5.1, R 4.2.1, admixtools q3pop v. 410, Twisst v. 0.2, beagle v. 4.1, PAST v. 4.11

For manuscripts utilizing custom algorithms or software that are central to the research but not yet described in published literature, software must be made available to editors and reviewers. We strongly encourage code deposition in a community repository (e.g. GitHub). See the Nature Portfolio guidelines for submitting code & software for further information.

Data

Policy information about availability of data

All manuscripts must include a data availability statement. This statement should provide the following information, where applicable:

- Accession codes, unique identifiers, or web links for publicly available datasets
- A description of any restrictions on data availability
- For clinical datasets or third party data, please ensure that the statement adheres to our policy

The genome data generated in this study have been deposited in the NCBI database under BioProject accession code PRJNA924716 and BioSample ID SAMN32782865. For the reference genome of Stenogyne calaminthoides, Hi-C reads are under accession SRR23341345, RNA-seq data under accession SRR23341344, Illumina shotgun data under accession SRR23341343, and Oxford Nanopore reads under accession SRR23341342. This Whole Genome Shotgun project has been deposited at GenBank under the accession JBBCBC000000000. The Stenogyne calaminthoides genome assembly and annotation used for analyses in this study is available on CoGe [https://genomevolution.org/coge/GenomeInfo.pl?gid=58017].
Raw reads for resequenced samples can be found under accession numbers SAMN32767766-SAMN32767919. Additional processed data are available in Dryad [https://doi.org/10.5061/dryad.ghx3ffbwc]. Processed data generated in this study and used for main text figures are provided in source data files.

Research involving human participants, their data, or biological material

Policy information about studies with human participants or human data. See also policy information about sex, gender (identity/presentation), and sexual orientation and race, ethnicity and racism.

Reporting on sex and gender	N/A
Reporting on race, ethnicity, or other socially relevant groupings	N/A
Population characteristics	N/A
Recruitment	N/A
Ethics oversight	N/A

Note that full information on the approval of the study protocol must also be provided in the manuscript.

Field-specific reporting

Please select the one below that is the best fit for your research. If you are not sure, read the appropriate sections before making your selection.

☐ Life sciences ☐ Behavioural & social sciences ☒ Ecological, evolutionary & environmental sciences

For a reference copy of the document with all sections, see nature.com/documents/nr-reporting-summary-flat.pdf

Ecological, evolutionary & environmental sciences study design

All studies must disclose on these points even when the disclosure is negative.

Study description	We carried out DNA sequencing, assembly, and annotation and generated a chromosome-scale reference genome of one Hawaiian mint species, Stenogyne calaminthoides. In addition, we resequenced 45 relatives, representing 34 species, to uncover the continental origins of this group and their subsequent diversification. We further resequenced 109 individuals of two Stenogyne species, and their purported hybrids, from Hawai'i's Mauna Kea volcano to detail recent gene flow among Hawaiian mint species.
Research sample	Samples were selected for this study based on availability of material and special consideration following our previous studies . Young leaf tissue was removed from a cultivated individual of Stenogyne calaminthoides and immediately flash frozen for high-molecular-weight DNA extraction. Silica-dried leaf material of 45 individuals of Hawaiian mints and relatives, as well as 109 individuals of Stenogyne rugosa and S. microphylla and their purported hybrids were used for genome resequencing.
Sampling strategy	Plant samples were selected for this study based on availability of material, findings from previous studies, and special consideration to the range of the lineage in question in terms of both geography, habitat, phylogeny, and morphology.
Data collection	High-molecular weight DNA was obtained from flash frozen mature leaves and RNA from various tissues (root, stem, young leaves). DNA of resequenced samples were obtained from mature leaves preserved in silica gel. To obtain a chromosome-scale reference genome, DNA was sequenced on using both Oxford Nanopore Technology and Illumina sequencing. DNA resequencing and RNA sequencing was performed with NovaSeq Illumina sequencing.
Timing and spatial scale	This project was based on plant material collected over the past 20 years. Data collection and analyses were performed in the period 2018-2023.

Data exclusions	No exclusion of data
Reproducibility	Raw read data is publicly available for download. Using the software versions and running options as reported in the paper will reproduce our results.
Randomization	No clinical experimentation was done in the paper, therefore randomization was not needed.
Blinding	Blinding was not possible since the analyses did not include experimental vs. control groups.

Did the study involve field work? ☒ Yes ☐ No

Field work, collection and transport

Field conditions	Mesic forest to high-elevation, dry and cool conditions.
Location	Hawaiian Islands
Access & import/export	Permission for collecting were provided from the Division of Forestry and Wildlife, the National Tropical Botanical Garden, and the Volcano Rare Plant Facility
Disturbance	Only small leaf samples were collected with little to no disturbance

Reporting for specific materials, systems and methods

We require information from authors about some types of materials, experimental systems and methods used in many studies. Here, indicate whether each material, system or method listed is relevant to your study. If you are not sure if a list item applies to your research, read the appropriate section before selecting a response.

Materials & experimental systems

n/a	Involved in the study	
☒	☐	Antibodies
☒	☐	Eukaryotic cell lines
☒	☐	Palaeontology and archaeology
☒	☐	Animals and other organisms
☒	☐	Clinical data
☒	☐	Dual use research of concern
☐	☒	Plants

Methods

n/a	Involved in the study	
☒	☐	ChIP-seq
☒	☐	Flow cytometry
☒	☐	MRI-based neuroimaging
